# Transcription Control Pathways Decode Patterned Synaptic Inputs into Diverse mRNA Expression Profiles

**DOI:** 10.1371/journal.pone.0095154

**Published:** 2014-05-01

**Authors:** Pragati Jain, Upinder S. Bhalla

**Affiliations:** 1 National Centre for Biological Sciences, Tata Institute of Fundamental Research, Bangalore, India; 2 Manipal University, Manipal, India; Tokai University, Japan

## Abstract

Synaptic plasticity requires transcription and translation to establish long-term changes that form the basis for long term memory. Diverse stimuli, such as synaptic activity and growth factors, trigger synthesis of mRNA to regulate changes at the synapse. The palette of possible mRNAs is vast, and a key question is how the cell selects which mRNAs to synthesize. To address this molecular decision-making, we have developed a biochemically detailed model of synaptic-activity triggered mRNA synthesis. We find that there are distinct time-courses and amplitudes of different branches of the mRNA regulatory signaling pathways, which carry out pattern-selective combinatorial decoding of stimulus patterns into distinct mRNA subtypes. Distinct, simultaneously arriving input patterns that impinge on the transcriptional control network interact nonlinearly to generate novel mRNA combinations. Our model combines major regulatory pathways and their interactions connecting synaptic input to mRNA synthesis. We parameterized and validated the model by incorporating data from multiple published experiments. The model replicates outcomes of knockout experiments. We suggest that the pattern-selectivity mechanisms analyzed in this model may act in many cell types to confer the capability to decode temporal patterns into combinatorial mRNA expression.

## Introduction

Long-term memory formation involves plasticity at synapses, but its consolidation requires protein synthesis and typically involves the activation of the cellular transcription machinery. Several mechanisms for plasticity and their mechanistic models are restricted to one or a small subset of synapses. These include biochemical pathways [1], receptor trafficking [2], and protein synthesis [3]. Other forms of plasticity have a restricted cellular localization, such as excitability modulation in dendrites [4]. In contrast to these local mechanisms, activity-dependent transcription control of synaptic plasticity genes has a unique position in that it is cell-wide and in a position to integrate inputs from the entire cell, and possibly to control plasticity across the cell [5]. There is a strong evidence that cell-wide plasticity effects are important in behavioral and systems level measures of learning [6]. For example, silencing of cells expressing activity-regulated cytoskeleton-associated protein (Arc) following an aversive stimulus abolishes the memory of the stimulus [7], [8].

There is substantial overlap of transcription control regulatory mechanisms between neuronal and non-neuronal cell types such as insulin-producing β-cells [9] and melatonin-synthesizing cells [10]. Transcription mediated by the cAMP response element-binding protein (CREB) has been shown to affect a variety of non-neuronal responses such as hematopoiesis, cell proliferation, acute leukemias [11], differentiation of adipocytes [12], cardiac myocytes [13] and smooth muscle cells [14]. For neuronal responses, CREB-mediated transcription has been shown to be involved in the formation of long-term memory [15] and also critical for late-phase LTP. The genes implicated in LTP are regulated by CREB-mediated transcription. These include BDNF [16], Calcium-calmodulin dependent protein kinase IV (CaMKIV), synapsin I, somatostatin, voltage-gated potassium channels, Fos, and Jun [17]. A few of these genes products are themselves inputs (e.g., BDNF) or essential components of the transcriptional control system (zif268, c-fos, C/EBPβ) [18]. In neuronal as well as non-neuronal contexts, the temporal pattern of input is important in determining the transcriptional outcome [19], [20]. Thus it is important to develop a mechanistic understanding of how different inputs as well as the timing of inputs regulate transcription control pathways.

Processes at many levels come together to effect the temporal and spatial regulation of mRNA synthesis during learning. There is substantial convergence of inputs during synaptic plasticity. For example, calcium influx through N-methyl-D-aspartate (NMDA) receptor and L-type calcium channel [21], neurotrophins like Brain-derived neurotrophic factor (BDNF) [22], and cyclic adenosine monophosphate (cAMP) signaling [23] have been shown to modulate mRNA synthesis and thus participate in formation of long term potentiation (LTP) [24]. In addition to these distinct pathway inputs, gene expression is also differentially controlled by the temporal and spatial parameters of the inputs. The timing, amplitude, and duration of calcium stimuli are known to be important, as is the spatial arrangement of multiple synaptic inputs [25]. The output of these signaling events is equally complex. About 300 genes are affected in response to neuronal activity [26]. These are mainly ion channels, receptors for growth or neurotrophic factors, protein kinases, or components of the neurotransmitter synthesis or release machinery [16]. Interestingly, many have an unique time-course of varying amplitude and duration [26]. Several studies have reported that behavioral stimuli such as visual experience and fear conditioning, as well as different synaptic stimuli and BDNF lead to synthesis of different mRNA subtypes [26]. For example, the single or multiple electroconvulsive seizure (ECS) treatments change expression of multiple genes in the hippocampus [27]. In the perforant path-granule cell (pp-gc) synapse, the high-frequency stimulation affects expression of zif/268, c-fos, c-jun and jun-B mRNA whereas low-frequency stimulation have no affect on the expression of these mRNAs [28]. In addition, the short term (3–6 h) exposure of BDNF induces expression of synapse-associated proteins whereas BDNF exposure for long-term (6–12 h) induces expression of immediate-early genes in hippocampal cultures [29]. Overall, it is clear that mRNA undergoes specific regulation by a wide range of stimulus attributes. It is not clear how these stimulus attributes influence subtype-specific changes in expression of mRNA during plasticity. Our analysis is designed to understand how different stimuli or distinct stimulus patterns result in differential gene expression.

In our study, we simulate CREB-mediated gene expression. We used postsynaptic calcium patterns to replicate synaptic input, and BDNF as an additional input. We modeled key regulatory pathways which are activated in the dendrites, soma, and nucleus of hippocampal neurons in response to synaptic and BDNF input. The model uses CaMKIV, MAPK and PP1 pathways to transfer the signal to the nucleus. We have parameterized the model by using the data obtained from published literature. The model suggests that this network of pathways performs a transformation of temporal stimulus patterns into a combinatorial code of mRNA expression.

## Results

**Figure 1:**
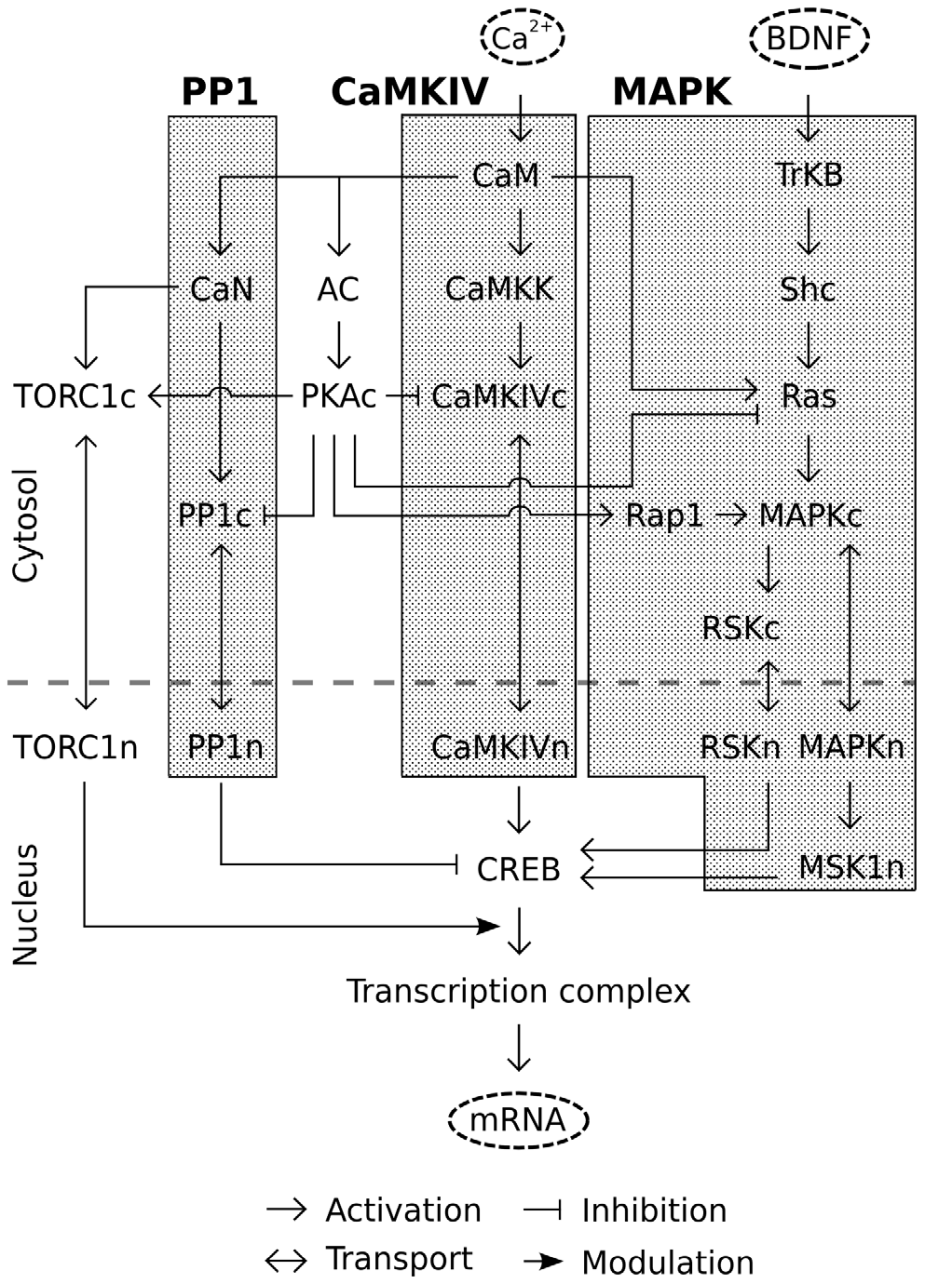
Block diagram of the model of pathways regulating mRNA synthesis. We incorporated three major pathways: Calcium-calmodulin dependent protein kinase IV (CaMKIV), Mitogen-activated protein kinase (MAPK) and Protein Phosphatase 1 (PP1). Each of these converged on CREB activation. We also modeled further interactions with Transducer of regulated CREB activity 1 (TORC1) and the protein kinase A (PKA) pathway. The inputs to the model were BDNF and Ca^2+^ waveforms configured to represent LTP- and LTD- inducing stimuli.

The major signaling pathways in our model were the CaMKIV pathway, MAPK pathway and PP1 pathway. The other pathways included in the model were PKA and TORC1 (Fig. 1). We first developed independent sub-models for the CaMKIV and mRNA synthesis portions of the model. These were independently parameterized. These models were merged with previously published models for CaM, BDNF input pathway (Fig. S1A in File S1), PP1 (Fig. S1B in File S1) and PKA (Fig. S1C in File S1) signaling inputs [30], [31]. We then validated the behaviour of the composite model. We deployed the composite model using simulated long term potentiation (LTP)- and long term depression (LTD)- induction protocols to predict the dependence of mRNA synthesis on CaMKIV, MAPK, CREB and TORC1. Finally, we postulated mechanisms for CaMKIV, MAPK and PP1 to regulate distinct subsets of mRNA synthesis, and used the model to predict the combinatorial regulation of mRNAs by different synaptic plasticity-inducing stimuli.

### CaMKIV Sub-model

**Figure 2:**
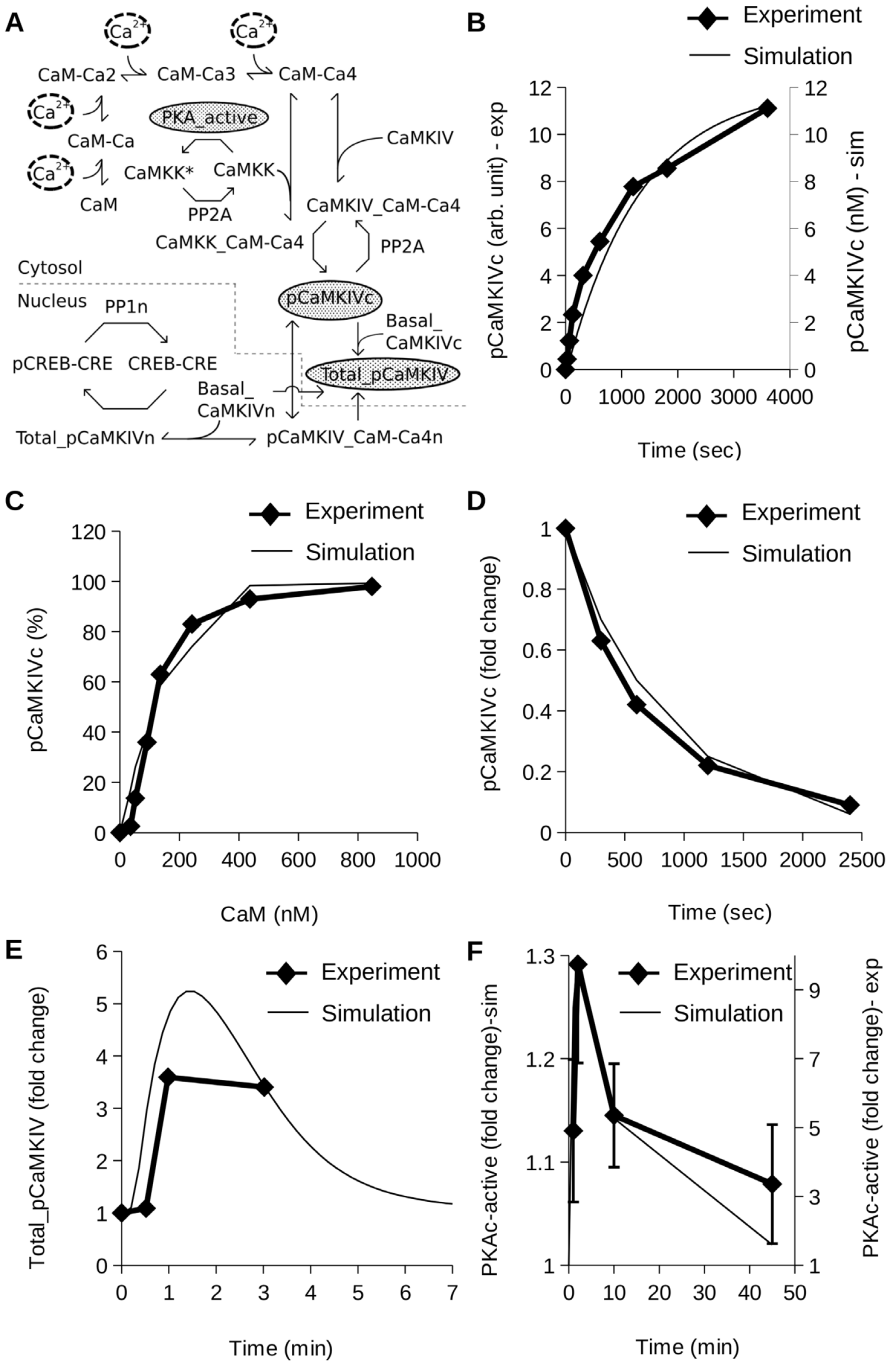
CaMKIV sub-model and parameterization. (A) Reaction diagram of CaMKIV sub-model. Shaded gray ovals highlight the molecules used for constraining the model. (B) Time course of activation of CaMKIV by CaMKK [38]. (C) Calmodulin dependence of CaMKIV activity [32]. (D) Time course of inactivation of phosphorylated CaMKIV by PP2A [33]. (E) Time course of Total_pCaMKIV in presence of 90 mM K^+^ solution for 1 min [39]. (F) Time course of active PKA in presence of LTP (three sets of stimuli at 10 min intervals, each set consisting of three 1 sec, 100 Hz tetani, 5 sec apart) [42].

The calcium-calmodulin-dependent protein kinase kinase (CaMKK) and Ca^2+^/calmodulin-dependent protein kinase IV (CaM-kinase IV) are both activated through the binding of Ca^2+^/CaM (Fig. 2A) [32]. Then, the Ca^2+^/CaM bound form of CaMKK phosphorylates and activates the Ca^2+^/CaM bound form of CaMKIV, leading to a substantial increase in its activity [33]. PP2A dephosphorylates and thus inactivates the phosphorylated form of CaMKIV [33]. There is a cross-inhibitory interaction between the PKA pathway and CaMKIV pathway. Active-PKA phosphorylates CaMKK to render it inactive, and hence the downstream CaMKIV is also inhibited [34]. Phosphorylated CaMKIV is transported to the nucleus [35]. It is known that there is a significant contribution of Ca^2+^/CaM independent CaMKIV activity [36] which is shown by a pool of basal CaMKIV in the model. The sum total of Ca^2+^/CaM independent and dependent CaMKIV contributes to the total activity of CaMKIV in the nucleus which then activates CREB at Ser-133 by phosphorylation [37] (Fig. 2A).

We have constrained the CaMKIV pathway model by simulating four previously published experiments, whose details we specify in order to compare against the simulated implementation of the experiment.

1. Time course of activation of CaMKIV by CaMKK: In the published experiment, an extract of CaMKIV (0.67 *µ*g/ml) from rat brain was mixed with CaMKIV kinase (0.07 *µ*g/ml), 0.2 mM CaCl2 and 2 *µ*M Calmodulin (CaM) [38]. The mixture was incubated for indicated times and the phosphorylated CaMKIV was measured using SDS-PAGE. In our model we used CaM-Ca4 (2 *µ*M) as the input and measured the levels of phosphorylated CaMKIV as a function of time (Fig. 2B).
2. Calmodulin dependence of CaMKIV activity: In the published experiment, CaMKK (36 nM) and CaMKIV (22 nM) were added to a mixture of 1 mM CaCl2 and the different concentrations of CaM [32]. The CaM dependence of activated CaMKIV was assayed by measuring the phosphorylation of substrate syntide-2 by activated CaMKIV. In the model we gave CaM-Ca4 as the input and measured the phosphorylated CaMKIV at 5 min as a measure of its activity (Fig. 2C).
3. Time course of inactivation of phosphorylated CaMKIV by PP2A: In the experiment, phosphorylated CaMKIV (152 nM) was incubated with PP2A for a range of durations between 0 and 2400 seconds, and then the CaMKIV activity was measured [33]. To simulate this experiment, we mixed phosphorylated CaMKIV and PP2A (150 nM) and compared experimental and model levels of phosphorylated CaMKIV as a function of time (Fig. 2D).
4. Parameters for CaMKIV activation by steady calcium stimuli: In the experiment, hippocampal culture neurons were stimulated by perfusing with 90 mM K^+^ solution for 1 min [39]. After the stimulus presentation, the level of phosphorylated CaMKIV (pCaMKIV) in crude cell lysates was monitored at different time-point (0, 30, 60 and 180 sec) by immunoblot. We have estimated the calcium generated by 90 mM K^+^ solution based on a previously published study [40]. Using this calcium stimulus as an input, we simulated the formation of active CaMKIV, measured as the sum of cytoplasmic and nuclear concentrations of active CaMKIV. The simulated time-course of Total_pCaMKIV matches the experimental time-course (Fig. 2E).

Thus, our CaMKIV activation model was able to semi-quantitatively reproduce each of these experiments.

### PKA Sub-model

The PKA model was based on previously published reaction schemes and parameters [41]. We selected only the CaM-mediated portion of the previous model, acting through ACI [30]. ACI synthesizes cAMP, which binds to and activates PKA. To fine-tune the PKA model parameters in the context of the composite model, we simulated the following previously published experiment:

LTP was induced in hippocampal slices using three sets of stimuli at 10 min intervals, each set consisting of three 1 sec, 100 Hz tetani, 5 sec apart [42]. After the final set of stimuli, the slices were frozen at different time points (0, 1, 2, 10 and 45 min). The CA1 area of the slice was micro-dissected, homogenized and incubated with PKA substrate for 5 min. Phosphate incorporation into substrate was measured to assay PKA-activity. To simulate the experiment, we presented a simulated LTP input to the composite model, with the same 3 stimuli every ten minutes. Each stimulus set in the simulation consisted of three calcium pulses of duration 1 sec and concentration 2 *µ*M, presented every 5 sec. The simulated PKA activity time course was smoothened by averaging the obtained trace over a 5 min sliding window point to account for the 5 min incubation time of the homogenized CA1 sample with the PKA substrate. The change in simulated PKA activity correlates with the experimental PKA activity (Fig. 2F).

### MAPK Sub-model

The MAPK model was an extension of a previously parameterized and published model from the DOQCS database [43] (Fig. S2 in File S1) [30]. We extended the previous model by incorporating activation of MAPK by B-Raf (Fig. S2 in File S1) in addition to the existing activation through C-Raf. This additional pathway was parameterized in a published modeling study [44]. The B-Raf activation pathway was modeled as follows: PKA activation induces activation of MAPK [45] by phosphorylating Src which then phosphorylates Cbl [46]. Active Cbl forms a complex with a bound form of CRK and C3G, and catalyse GDP/GTP exchange reaction of Rap1 [44], [47]. Rap1GAP activates intrinsic GTPase activity of Rap1 [48]. Rap1GTP interacts with B-Raf and activates it [49]. Active B-Raf leads to the activation of MAPK [50]. The B-Raf pathway contributes to the slow phase of active MAPK formation (Fig. S3A in File S1).

**Figure 3:**
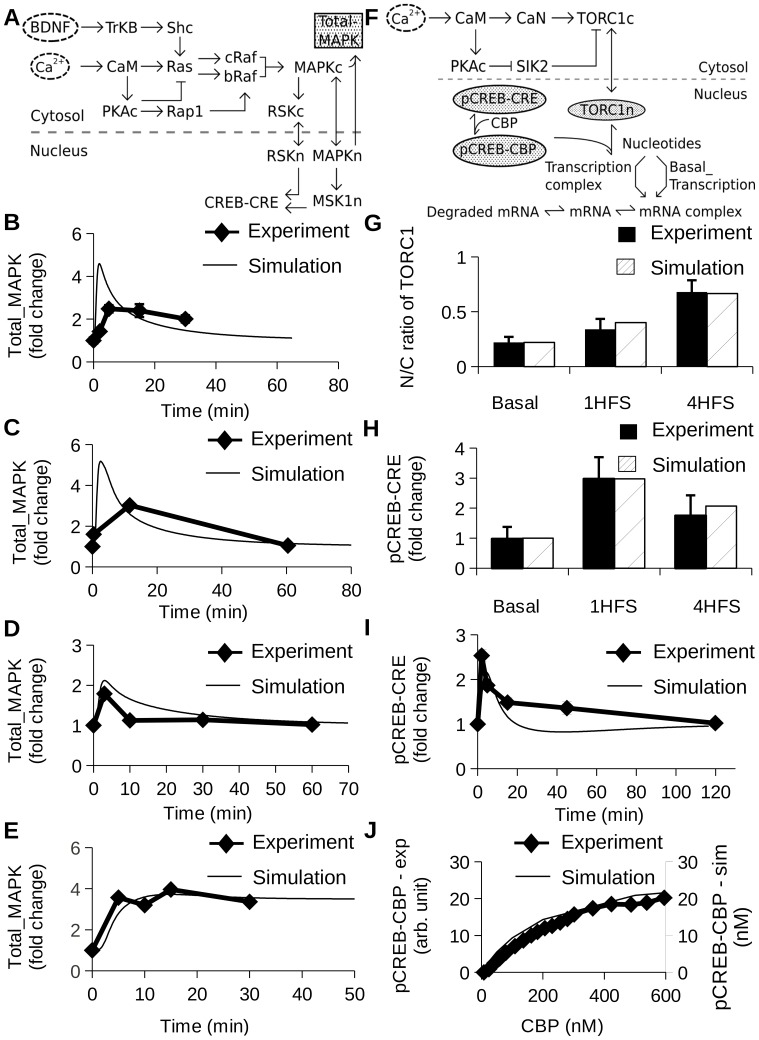
MAPK and TORC1/CREB sub-models and parameterization. Except for panel G and J, all graphs report how many fold the measured molecule changes with respect to baseline levels. (A) Block diagram of MAPK sub-model. The shaded gray oval highlights total phosphorylated MAPK, which was compared with experiments in panels B, C, D and E. (B) Time course of Total_MAPK in presence of LTP (100 Hz for 1 sec) [55]. (C) Time course of Total_MAPK in presence of 90 mM K^+^ solution for 3 min [40]. (D) Time course of Total_MAPK following an LTP stimulus [58]. A tetanus of 100 Hz frequency and 1 sec duration was applied two times with a 20 sec interval between tetani. (E) Time course of Total_MAPK [59] in presence of 2 nM BDNF stimulus for indicated time points. (F) Block diagram of sub-model including TORC1, CREB and mRNA synthesis (G) N/C fluorescence ratio of TORC1 in presence of L-LTP (four pulses of 100 Hz for 1 sec after every 5 min) [60]. (H) Relative increase in pCREB-CRE in presence of L-LTP (four pulses of 100 Hz for 1 sec after every 5 min) [60]. (I) Time course of pCREB-CRE in presence of 50 Hz for 18 sec [67]. (J) Dose-response for pCREB-CRE with change in CBP [68].

We further extended the earlier MAPK model by including downstream steps involving RSK, PDK1, and nuclear transport (Fig. S2 in File S1). Active MAPK phosphorylates RSK which then undergoes auto-phosphorylation. RSK is further phosphorylated by PDK1, which leads to its full activation [51]. Active RSK is transported to the nucleus where it phosphorylates CREB at Ser-133 [52], [53]. Another substrate for active MAPK is MSK which expresses exclusively in the nucleus. Active MAPK is transported from the cytosol to the nucleus, where it phosphorylates MSK1 which then activates CREB at Ser133 [53], [54]. We modeled all these steps in the MAPK pathway (Fig. 3A). The newly incorporated reactions in the model were parameterized as a part of the composite model by using published experiments. We simulated four previously published experiments to constrain the MAPK pathway model.

1. Parameters for MAPK activation by E-LTP stimuli: In the published experiments, the early phase of LTP (E-LTP) was induced at CA1 neurons in hippocampal slices [55]. The stimulus used to induce E-LTP was high-frequency stimulation HFS, a 1 sec train of 100 Hz. After the induction of E-LTP, slices were frozen at various time-points (0, 2, 5, 15 and 30 min) to measure phosphorylated forms of MAPK by using western blots. To simulate this experiment, we delivered a single calcium pulse of 2 *µ*M for 1 sec, to represent the HFS stimulus [56], [57]. As output we measured total MAPK (Total_MAPK), computed as the sum of cytoplasmic and nuclear concentration of active MAPK. The simulation gave a qualitative match to the experimental time-course of MAPK pathway (Fig. 3B).
2. Parameters for MAPK activation by steady calcium stimuli: In the published experiments, hippocampal culture neurons were perfused with 90 mM K^+^ solution for 3 min. The level of the phosphorylated MAPK (pMAPK) bound to the specific antibodies was measured by western blots [40]. The authors also measured intracellular calcium using fluorescence imaging generated by 90 mM K^+^ solution for 3 min. We have used the same calcium time-course measurement as a stimulus to the model. We computed Total_MAPK (Fig. 3C) to account for all the phosphorylated forms of MAPK. Again the match of simulation to experiment was qualitative.
3. Parameters for MAPK activation by LTP stimuli: In the published experiments, hippocampal slices were used to measure the time course of MAPK (pMAPK) [58]. LTP was induced in the CA1 region by electrical stimulation. A HFS of 100 Hz frequency and 1 sec duration was applied two times with a 20 sec interval. After stimulation, the slices were homogenized and the measurements were done by western blots using specific antibodies. We modeled the experiment by presenting the stimulus as two pulses of calcium separated by 20 sec. The duration of each calcium pulse was 1 sec [56] and concentration was 2 *µ*M [57]. The simulated time course of Total_MAPK (Fig. 3D) matches the experimental time-course of pMAPK with the exception of the third data point.
4. Parameters for BDNF activation of MAPK: In the experiment, cortical neurons were stimulated with steady application of 2 nM BDNF [59]. The tyrosine phosphorylation of MAPK was measured for various time points (0, 5, 10, 15 and 30 min) using western blots. We simulated the experiment by providing BDNF for the entire time-course and measured Total_MAPK (Fig. 3E). The simulated Total_MAPK closely matched the experiment data.

The MAPK model was based on numerous data sources and the current parameterizing runs were challenged by apparently incompatible time-courses of responses in some cases (Figures 3B, 3C, and 3D). We therefore regard the model as semi-quantitative, and rather than try to precisely match one or two experiments, we sought to approximate several. Later we carry out parameter sensitivity analyses to show that the resultant model behaves robustly to parameter changes.

### TORC1 Sub-model

TORC1 is the Transducer of regulated CREB activity 1, and is also called CRTC (CREB-regulated transcriptional co-activator). TORC1 is abundantly expressed in the brain and plays a role in the late phase of hippocampal long-term potentiation [60]. Under basal conditions, Salt-inducible Kinase 2 (SIK2) phosphorylates TORC1 at Ser171 and stimulates binding of 14-3-3 protein to phosphorylated TORC1 in the cytoplasm [61], [62]. We modeled it as a pool called pTORC1 which represents phosphorylated TORC1 in a complex form with 14-3-3 protein. Upon activation of calcium and cAMP pathways, TORC1 dissociates from 14-3-3 protein. It has been proposed that SIK2 serves as a negative feedback signal that prevents TORC1 dependent transcription [61]. Active PKA phosphorylates active SIK2 and reverses this effect by activating the translocation of TORC1 to the nucleus [61] (Fig. S3B in File S1). The phosphorylated TORC1 undergoes dephosphorylation in presence of calcium dependent phosphatase (PP2B, CaN) which leads to the translocation of TORC1 to the nucleus [63]. PKA and PP2B mediate synergistic effect on TORC1 dependent transcription by decreasing the level of phosphorylated TORC1 and increasing the level of unphosphorylated TORC1 in the nucleus. The nuclear TORC1 binds to the CREB and upregulates CRE-dependent transcription [64] (Fig. 3F). This sub model was parameterized as a part of the composite model by using published experiments, as follows.

In a previously published study, immunohistochemical experiments were performed in the hippocampal slice preparation to measure the ratio of nuclear to cytosol TORC1 (N/C fluorescence ratio) and the relative increase of phosphorylated CREB, in CA1 neurons. These measurements were made for basal stimulation, E-LTP and L-LTP stimuli [60]. Basal stimulation was induced at 0.033 Hz for 30 min. E-LTP was induced by a train of HFS at 100 Hz for 1 sec (1HFS) and L-LTP was induced by four trains of HFS (100 Hz for 1 sec) presented after every 5 min (4HFS). In the simulation, each HFS was represented by a 1 sec pulse of calcium at 2 *µ*M. We computed the ratio of nuclear TORC1 (TORC1n) to cytoplasmic TORC1 (TORC1c) (N/C ratio of TORC1) and pCREB-CRE as a readout for experimental N/C TORC1 and pCREB respectively. In the experiment, the level of TORC1 and pCREB for basal stimulation remained close to the level obtained from unstimulated slices. We have estimated a level of N/C TORC1 and pCREB-CRE for basal stimulation from the unstimulated model to measure the relative change following basal, 1HFS and 4HFS stimulation (Fig. 3G and 3H). We were able to match the simulation output to experimental result to within the error bars.

Thus, the TORC1 model is mechanistically reasonably well-defined but somewhat under-constrained with respect to detailed parameters. We have been able to simulate a couple of experiments that measure the overall input-output relationships of this pathway, but clearly there are other parameter sets that would achieve these outcomes. Since our composite model depended primarily on the input-output relationships, we proceeded with this as a sufficient semi-quantitative approximation.

### CREB Regulation and mRNA Synthesis Sub Model

In the basal state CREB is bound as a dimer to the CAMP response element (CRE) sites in the promoter regions of target DNA [65]. We modeled it as a pool where CREB is in a complex form with DNA. When activated, this bound form of CREB binds to the CREB-binding protein (CBP) [66]. This interaction allows CREB to bind to the transcriptional machinery and thus, promote transcription. In our model we simulated the transcriptional machinery as a monolithic complex which synthesizes mRNA. We have also incorporated the basal mRNA synthesis for account of synthesis of mRNA independent of CaMKIV and MAPK pathways (Fig. 3F).

We modeled the following published experiment to estimate parameters for CREB phosphorylation by Ca^2+^ stimuli: Cultured hippocampal neurons were subjected to field electrical stimulation (18 sec, 50 Hz) [67]. Immunoreactivity of phosphorylated CREB in the nucleus was measured using specific antibodies, at different time-points following the stimulus (0, 2, 5, 15, 45 and 120 min). The change in intracellular free calcium in response to stimulus was measured by Fura-2 imaging. The measured change in the level of intracellular calcium is presented as an input stimulus to the composite model. After the stimulus presentation to the model, we recorded the time-course of pCREB-CRE (phosphorylated form of CREB) and plotted against the experimental pCREB. The simulated time course approximated that of the experiment except at the 50 min data point (Fig. 3I).

We further constrained the CREB-CBP portion of the model using a published dose-response curve for pCREB-CBP formation as a function of CBP concentration. In this experiment, 30 nM pCREB was incubated with indicated concentrations of CBP for 2 min [68]. The binding of phosphorylated CREB to CBP was quantified using fluorescence anisotropy. To model this experiment, we set the concentration of pCREB-CRE to 30 nM and systematically varied the concentration of CBP in a range from 1 nM to 1 *µ*M. We then ran the simulation for 2 min and recorded pCREB-CBP, a complex form of phospho-CREB and CBP. The simulation closely matched the experiment result (Fig. 3J).

Thus we were able to simulate two distinct experiments leading to the phosphorylation of CREB.

### Parameterizing the Composite Model

The composite model was constructed by merging the individual sub-models: CaMKIV pathway model, MAPK pathway model, PP1 pathway model, PKA pathway model and mRNA synthesis model. In each of these individual sub-models, we have included basal activation of the output molecules to account the effect of other signaling pathways on the activation of these molecules. Later the sub-models are merged by consolidating common molecules in different sub-models, such that the ‘output’ molecules of one sub-model, and the ‘input’ molecules of the next, are now one entity. In a few cases ‘output’ molecules act as enzymes, for example, as upstream kinases, that act on molecules of the next sub-model. In these cases the enzyme rates are estimated as before from literature sources, and refined in the composite model as below. The underlying sub-model remains unchanged.

**Figure 4:**
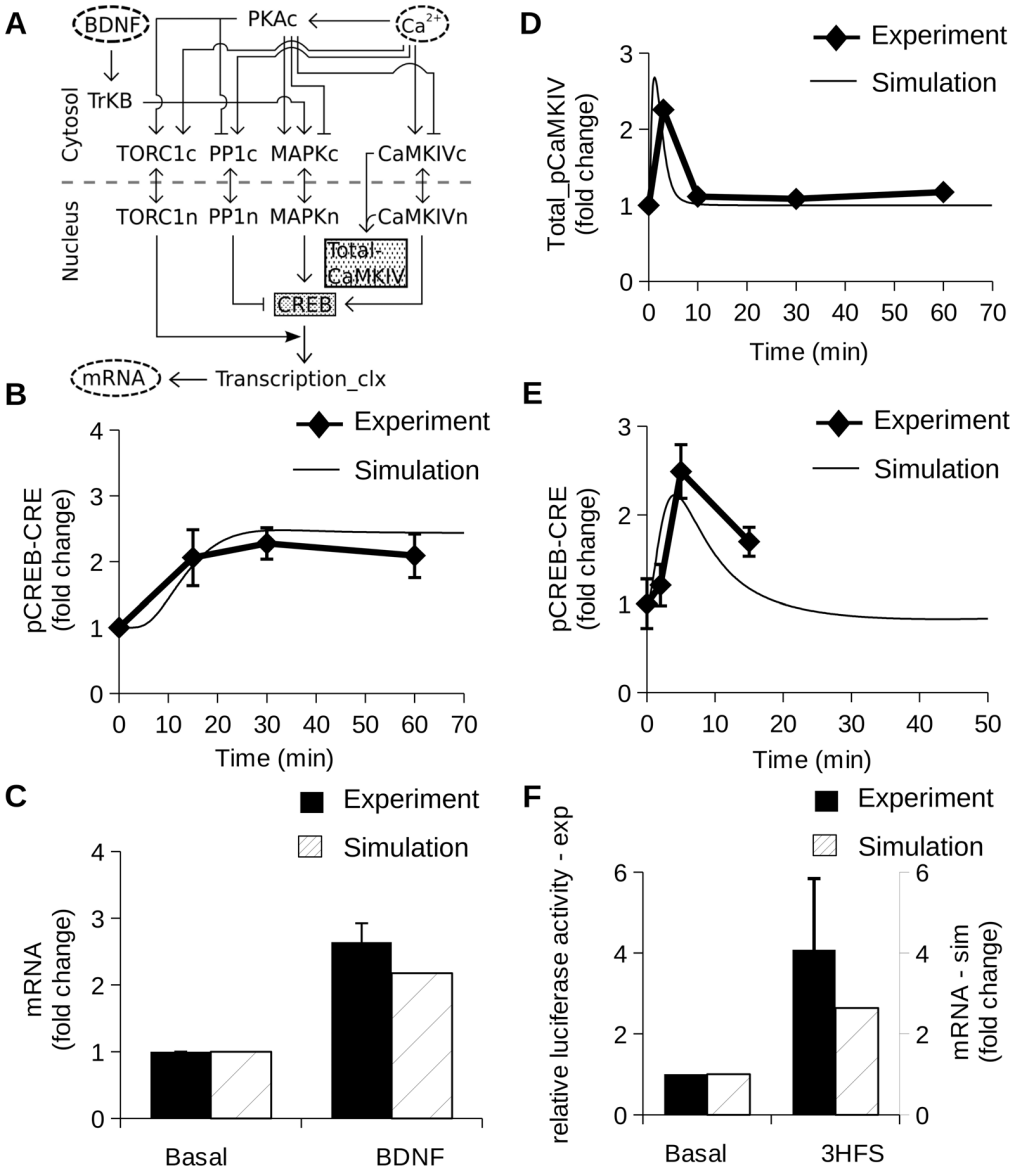
Composite model and parameterization. All the graphs show relative increase in level of read-outs with respect to baseline level. (A) Block Diagram of composite model with calcium and BDNF as an input. The shaded gray boxes represent the molecules measured for constraining the model. (B) Time course of pCREB-CRE [69] in presence of 2 nM BDNF stimulus for indicated time points. (C) Relative increase in mRNA synthesis rate following 4 nM BDNF stimulation [70]. (D) Time course of Total_pCaMKIV following an LTP stimulus [58]. A tetanus of 100 Hz frequency and 1 sec duration was applied two times with a 20 sec interval between tetani. (E) Time course of pCREB-CRE in presence of E-LTP (100 Hz tetanus for 1 sec) [55]. (F) Relative increase in mRNA synthesis after LTP induction (three pulses of 100 Hz for 1 sec separated by 5 min) [71].

Above, we had parameterized separate sections of this model. To constrain the interactions between the sub-models and also to monitor the flow of signals as they propagate through the composite model, we simulated five stimulus-response experiments. Some of the interactions between the sub-models were already parameterized in an earlier study, for example the interaction between CaM and Ras [30]. The parameters were not adjusted in the composite model and remain the same as they were used while constraining the sub-models. We monitored activation of CaMKIV, CREB, and mRNA synthesis rate to assess the quality of the parameterization in the composite model (Fig. 4A).

1. Parameters for BDNF activation of CREB: In the published experiment, cortical neurons were stimulated with BDNF for indicated time points (0, 15, 30 and 60 min) and phospho-Ser^133^ CREB (p-CREB) was measured using western blots [69]. To simulate this, we presented a steady 2 nM BDNF stimulus and measured the levels of pCREB-CRE (Fig. 4B). The simulated pCREB-CRE closely matched the experiment data.
2. Parameters for BDNF activation of mRNA synthesis: In this published experiment, the relative increase in mRNA synthesis following BDNF stimulation was measured [70]. The experiment was carried out in cultured hippocampal neurons where a steady stimulus of 100 ng/ml (4 nM) BDNF was delivered and the CRE mediated luciferase activity was measured as a readout for CREB-dependent transcription. We simulated the experiment by measuring the relative increase in mRNA synthesis following steady BDNF stimulation (Fig. 4C). The simulated increase in mRNA synthesis was close to the experimental value.
3. Parameters for CaMKIV by LTP stimuli: In the experimental study, LTP was induced in hippocampal CA1 region by electrical stimulation to measure the time course of phosphorylated CaMKIV (pCaMKIV) [58]. The stimulus used was a HFS of 100 Hz frequency and 1 sec duration was applied two times with a 20 sec interval. After stimulation, the slices were homogenized and the measurements were done by western blots using specific antibodies. We modeled the experiment by presenting the stimulus as two pulses of calcium separated by 20 sec. The duration of each calcium pulse was 1 sec [56] and concentration was 2 *µ*M [57]. We measured Total_pCaMKIV (Fig. 4D) to account for all the phosphorylated forms of CaMKIV. We were able to match the time-course of pCaMKIV.
4. Parameters for CREB phosphorylation by E-LTP stimuli: In the published experiment, E-LTP was induced at CA1 neurons in hippocampal slices [55]. The stimulus used was HFS (100 Hz for 1 sec). The level of the phosphorylated form of CREB was measured at different time-points (0, 2, 5 and 15 min) using western blots. In the simulation, we presented the stimulus as a pulse of calcium (2 *µ*M concentration and 1 sec wide). The levels of pCREB-CRE were monitored after the stimulus presentation. The simulated time course of pCREB-CRE was close to that of experiment (Fig. 4E).
5. Parameters for mRNA synthesis following L-LTP stimulus: In the experiment, LTP was induced in cultured hippocampal neurons by tetanic stimulation [71]. The stimulus consisted of three trains of HFS (100 Hz for 1 sec) (3 HFS) presented at an interval of 5 min. CRE-mediated luciferase activity was measured as a correlate of activity-induced gene expression. We simulated the experiment by presenting the stimulus as three pulses of calcium (1 sec wide and 2 *µ*M amplitude) to the composite model and measured the relative increase in mRNA synthesis at 5 min after the stimulus presentation. The simulated increase in mRNA synthesis was compared with the CRE-mediated luciferase activity and was within the error bars of experimental data (Fig. 4F).

At this point we had concluded the first part of our model development, that is, constraining and parameterizing the model using a range of published experiments.

### Parameter Sensitivity Analysis Demonstrates Robustness of Model

**Figure 5:**
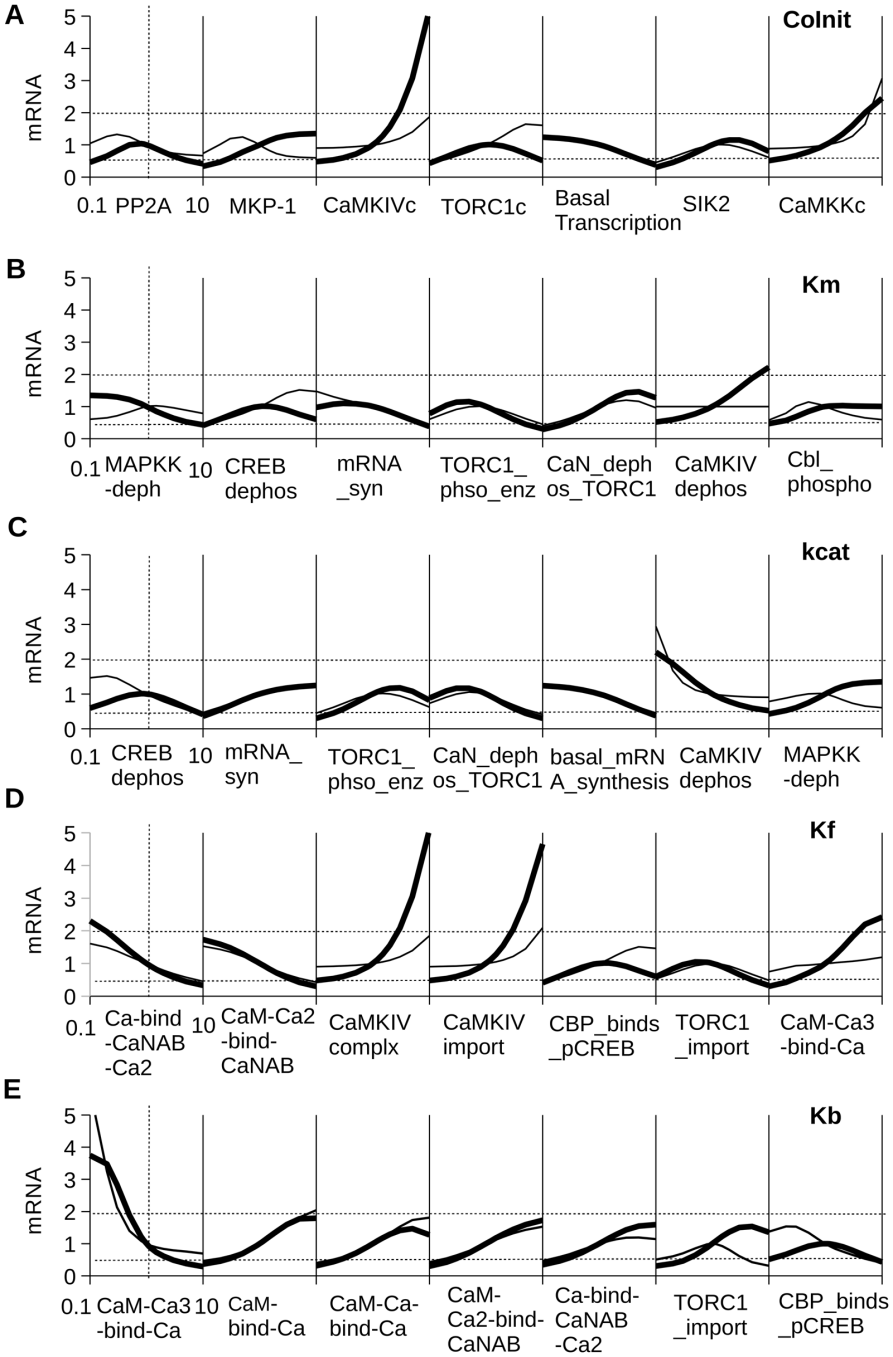
Parameter sensitivity analysis. The reference model parameter was varied by 0.1 to 10 fold to measure mRNA synthesis rate. The measured value was divided by the reference parameter model value to obtain the fold change. The parameters which show greater than two fold change in the response are plotted. The measurements done at 1000(A) Sensitivity Analysis for intial concentration (CoInit) (B) Sensitivity Analysis for Michaelis constant for enzymatic reactions (Km). (C) Sensitivity Analysis for turn over number for enzymatic reactions (kcat). (D) Sensitivity Analysis for forward rate of binding reations (Kf). (E) Sensitivity Analysis for backward rate for binding reactions (Kb).

In order to test the robustness of the model, we performed a parameter sensitivity analysis. If the model is robust there will not be significant change in intermediate molecules and mRNA synthesis over a wide range of parameters, thus mimicking the in vivo condition where a small change in the system does not lead to much change in its behaviour. Here we altered each parameter over a 100-fold range (0.1 to 10× reference values). For molecular pools, we varied the initial concentration (CoInit). For enzymes we varied Michaelis Constant of enzyme (Km) and turnover number of an enzyme (kcat). For non-enzymatic reactions we varied the forward (Kf) and backward rate constants (Kb). As a readout of the effect of these parameter variations, we monitored the simulated concentrations of Total_pCaMKIV (Fig. S4 in File S1), Total_MAPK (Fig. S5 in File S1), pCREB-CRE (Fig. S6 in File S1) and also the mRNA synthesis rate (Fig. 5). Almost all (360/418) parameters elicited a smaller than two-fold effect on these responses. The parameters which have a greater than two-fold effect on responses are plotted in Fig. 5. Most of these sensitive parameters correspond to two pathways of the model: CaMKIV pathway and MAPK pathway. In addition, the other sensitive parameters were the basal synthesis of mRNA, and SIK2 which prevents the translocation of TORC1 by phosphorylating TORC1 (Fig. 3F). We have tested the sensitivity of mRNA synthesis rate for various calcium-input patterns (Fig. S7 in File S1). In each case, the stimulus consists of three pulses of Ca^2+^ presented with a 5 min spacing (Fig. S7A in File S1). To test for sensitivity we varied the waveform of the Ca^2+^ pulse. In addition to the reference pulse of 2 uM amplitude and 1 second duration (Fig. S7B in File S1), we delivered a pulse of 1 *µ*M amplitude and 2 sec wide (Fig. S7C in File S1), and a time-varying pulse having a peak of 2 *µ*M amplitude near the end of the pulse which then decayed with a half-time of ∼5 sec to the baseline level (Fig. S7D in File S1) [72], [73]. Overall, the time-course of simulated mRNA synthesis was not sensitive to the Ca^2+^ waveform, but the peak of the mRNA synthesis rate was moderately sensitive to the peak Ca^2+^ levels.

### Different Pathways Mediate Calcium Dependent mRNA Synthesis

**Figure 6:**
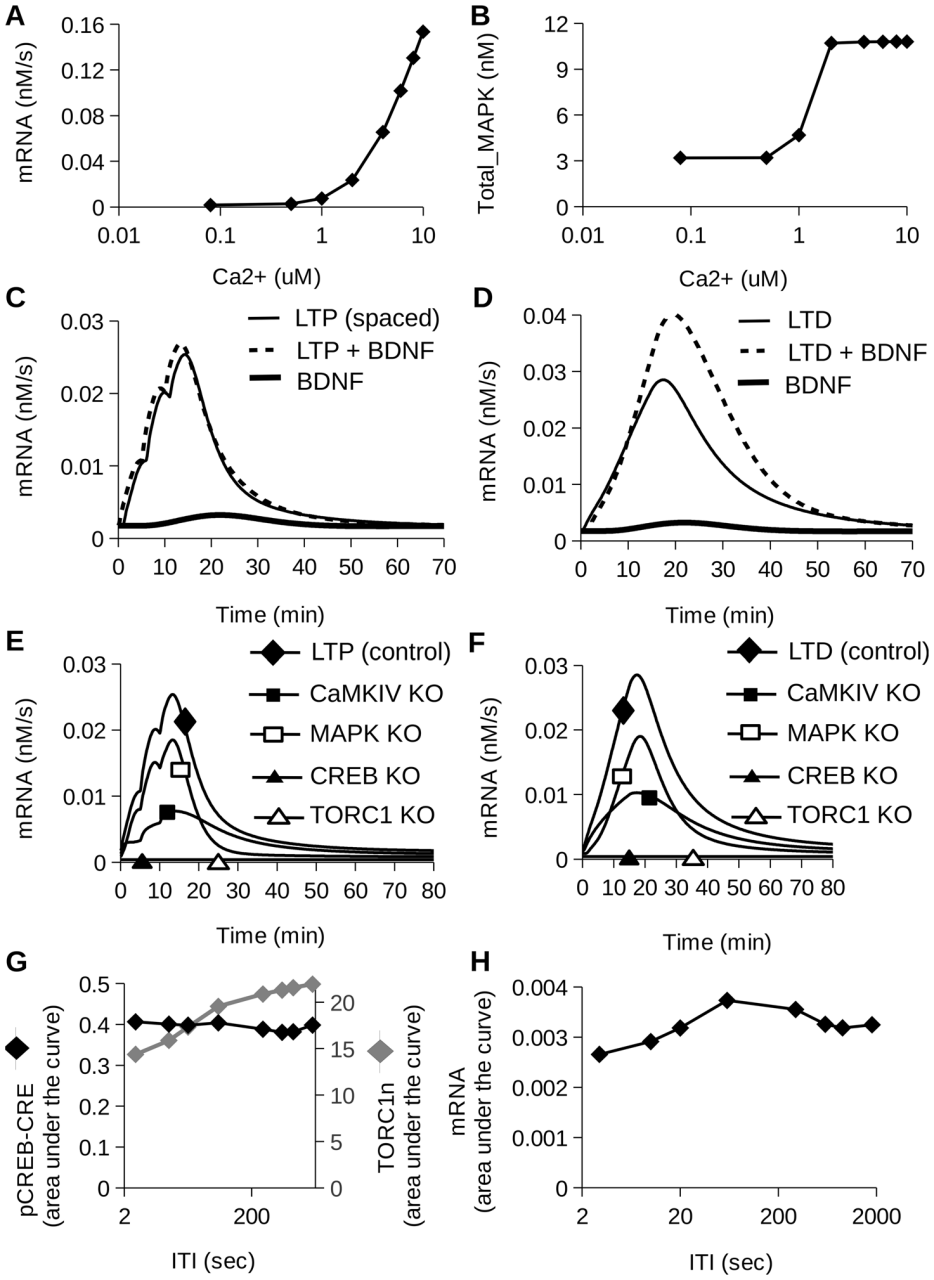
Effect of inputs and pathway knockouts on mRNA synthesis. Calcium dose-response relationship for mRNA synthesis rate (A) and Total_MAPK (B). (C) The rise in mRNA synthesis is negligible when BDNF input is combined with LTP stimulus (D) mRNA synthesis responds more strongly when BDNF was combined with LTD stimulus. (E) Control and knockout responses to LTP stimulus. (F) Control and knockout responses to LTD stimulus. CREB and TORC1 knockouts completely abolished the mRNA response as they are immediate upstream regulators. CaMKIV and MAPK knockouts had intermediate effects on mRNA. (G) Temporal tuning of pCREB-CRE and TORC1n. (H) Temporal tuning of mRNA synthesis rate. We plotted area under the curve after the stimulus presentation for Fig. 6G and 6H.

We next performed a characterization of model responses to patterned Ca^2+^ stimuli. The stimulus used was three pulses of calcium separated by 300 sec, each pulse of 1 sec duration. We measured the response to varied levels of calcium pulses (i.e. 0.08, 0.5, 1, 2, 4, 6, 8 and 10 *µ*M). We measured the readouts at 15 min after the stimulus presentation. As expected, the mRNA synthesis rate increased with an increase in calcium level (Fig. 6A). Upstream of the mRNA synthesis, we observed a calcium-dependent increase in Total_pCaMKIV (Fig. S8A in File S1) and pCREB-CRE (Fig. S8B in File S1) whereas Total_MAPK level saturated at around 2 *µ*M of Ca^2+^ stimulus amplitude (Fig. 6B). This divergence in response profiles and calcium dependence suggests that different pathways may selectively activate different temporal or molecular components of the overall mRNA-synthesis response.

We repeated the analysis for BDNF stimulation. We ran the model to steady state and then applied a steady stimulus of BDNF at the indicated concentration. Ca^2+^ was maintained at basal levels for first set of simulations. For a second set of simulations we presented BDNF along with a Ca^2+^ stimulus (three pulses of calcium of 2 µM amplitude given for 1 sec after every five min). We measured the readouts at 18 min after the stimulus presentation. When the BDNF stimulus was given at basal calcium (0.08 µM) the mRNA synthesis responded in a sigmoid manner to increasing levels of BDNF. When the BDNF stimulus was delivered along with the Ca^2+^ stimulus, the baseline due to Ca^2+^ was already high and the addition of BDNF had little effect on the response. Hence, BDNF effects were occluded by high calcium (Fig. S9A in File S1). We observed a sigmoid dependence of phosphorylated MAPK on BDNF (Fig. S9B) but there was no effect of BDNF on CaMKIV responses (Fig. S9C in File S1). This is expected, because there are no downstream interactions of BDNF leading to the CaMKIV pathway in our model. Hence, we attributed the effect of BDNF on the mRNA response to the MAPK pathway.

### BDNF Raises mRNA Synthesis When Combined with LTD, but not LTP, Stimulus

The above simulations suggested that distinct synaptic input patterns might have differential sensitivity to BDNF, because the amplitude and duration of calcium influx is pattern dependent [74]. We therefore presented LTP- and LTD- inducing stimuli along with BDNF. The LTP stimulus was delivered as three pulses of Ca^2+^ influx, each 1 sec wide separated by 300 sec plus a BDNF input of 2 nM for 600 sec. The LTD stimulus was presented as a single 900 sec Ca^2+^ pulse along with a BDNF input to 2 nM for 900 sec. We chose this duration for BDNF input based on our earlier study [31] where we measured protein synthesis as a function of time after the delivery of stimulus. The contribution of BDNF to model response was negligible when presented with LTP (Fig. 6C), but significant with LTD (Fig. 6D). This is might be due to the occlusion of the BDNF effect on mRNA synthesis rate at high levels of calcium as observed above and in Fig. S9 in File S1. This is in agreement to a published study where BDNF LTP is occluded by HFS-LTP [75].

### The Model Predicts the Outcome of Pathway Knockout Experiments

Knockout experiments have demonstrated the role of CaMKIV [76], ERK [15], CREB [77], [78] and TORC1 [60] in activity-dependent gene transcription. We analyzed the dependence of mRNA synthesis on these key molecules, for LTP and LTD inputs. To simulate knockout, we individually set CaMKIV, MAPK, CREB and TORC1 to zero and measured mRNA synthesis rate following different kinds of stimuli. The LTP stimulus caused an increase in mRNA level through the combined action of different pathways (Fig. 6E). The mRNA level was sharply attenuated when CaMKIV, CREB, or TORC1 was knocked out. The MAPK-knockout had an approximately 25% reduction on mRNA response. This panel of effects matches well with previously published experiments. For example, in brain-slice experiments on CaMKIV/Gr KO mice stimulated with glutamate, c-Fos staining of hippocampal CA1 neurons is much reduced compared to controls [76]. Further, dominant negative-TORC1 hippocampal neurons show no increase in KCl-induced gene expression in contrast to wild-type neurons [60]. The modest MAPK reduction is comparable to an experimental study showing around 35% decrease in gene expression when MEK inhibitor was used [15]. For LTD, we found that CaMKIV-knockout substantially reduced mRNA synthesis, and CREB- and TORC1-knockout almost abolished it. MAPK-knockout in our model had only a 30% reduction on mRNA synthesis during LTD (Fig. 6F). To our knowledge there have been no direct experimental readouts of mRNA synthesis under these conditions, hence these model predictions remain to be tested.

Overall, our model replicated a range of outcomes from knockout studies on key upstream pathways in translational regulation.

### mRNA and Upstream Pathways are Tuned to Interstimulus Interval

We next examined how mRNA and other pathways in the model responded to a sequence of strong synaptic inputs, at different temporal intervals. This set of simulations addresses tuning to massed as opposed to spaced inputs, which are known to elicit different forms of LTP [79]. Three pulses of 100 Hz for 1 sec were presented at varying inter-tetanus intervals (ITI), from 1 sec to 1800 sec (Fig. S8C in File S1). Each individual pulse was 1 sec long and had an amplitude of 2 *µ*M. We considered three readouts of temporal tuning: the activation of phosphorylated CREB, nuclear level of TORC1 (TORC1n) (Fig. 6G) and mRNA synthesis rate (Fig. 6H). In each case we considered the time-integral of the response after the stimulus presentation (measured as area under the time-series curve). CREB exhibited little tuning, TORC1 increased 50% with longer intervals, and mRNA synthesis peaked at 60 seconds. This 60-sec tuning is consistent with an earlier experimental study which showed an increase in activation of CREB-dependent gene expression with widely spaced training as opposed to massed training [80].

### mRNA Transcripts are Differentially and Combinatorially Synthesized Depending on Stimulus Pattern

**Figure 7:**
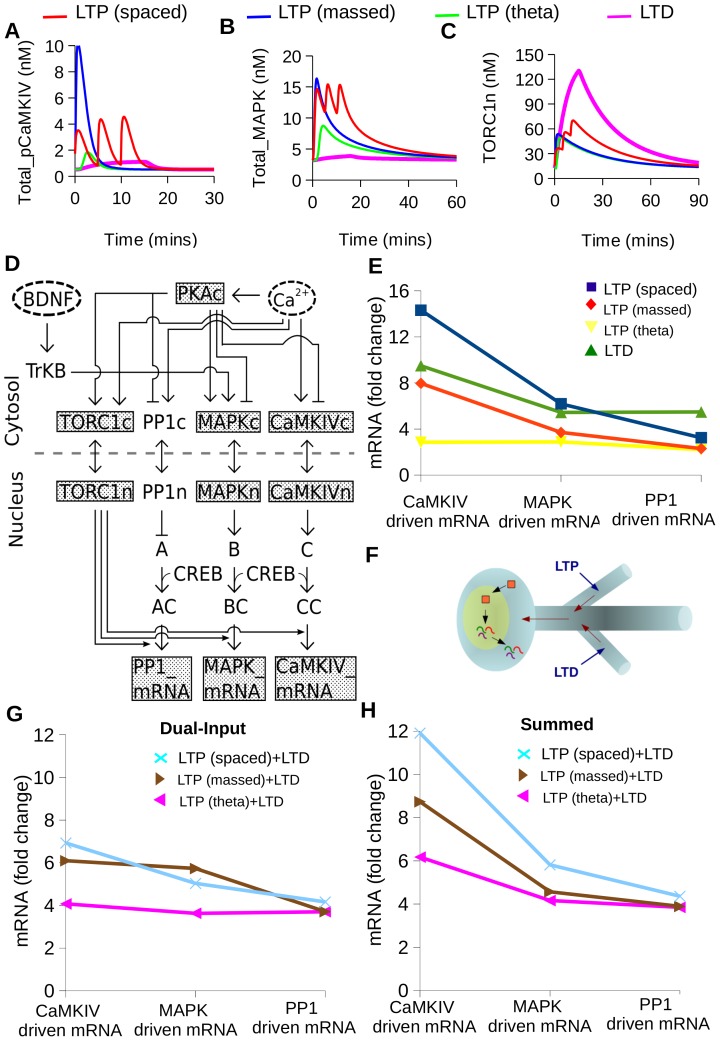
Differential synthesis of mRNA. Distinct activation profiles of (A) Total_pCaMKIV, (B) Total_MAPK and (C) TORC1n to four different temporal patterns of stimulation (spaced-LTP (ITI - 300 sec), massed-LTP (ITI - 10 sec), theta burst, and LTD). (D) Block diagram of an alternate model where CaMKIV, MAPK and PP1 pathways each leading to synthesis of different mRNA transcripts. (E) Distinct expression profiles of different mRNA transcripts for each of the four stimulus patterns. (F) Schematic showing two different branches of a neuron where one branch receives LTP stimulus and the other branch receives LTD stimulus. The LTP and LTD inputs are represented by blue arrows and the direction of information flow is shown by red arrows. The red blocks represent signaling molecules and the wavy colored lines represent mRNA (G) mRNA expression profiles for combinations of stimulus patterns. The stimulus combination presented were LTD plus different variants of LTP stimuli. (H) Expression profile for combined input computed by the arithmetic sum of the contributions from individual inputs. This is strikingly different from the simulated response in Fig. 7G.

As a key prediction of the model, we next asked whether the mRNA transcriptional control network might lead to differential stimulus dependent transcription of distinct mRNA sequences. In order to test this prediction of pattern selectivity in the transcriptional control network, we delivered four different stimulus patterns to the model: spaced-LTP (ITI - 300 sec), massed-LTP (ITI - 10 sec), theta burst, and LTD. We observed distinct activity patterns of CaMKIV, MAPK and TORC1 to these four stimuli (Fig. 7A, 7B and 7C). While these were complex temporal patterns of activity in their own right, we hypothesized that the mRNA synthesis reactions might act as a temporal integrator and thus transform these activity patterns into expression-level readouts.

To test this hypothesis, we extended our model with the assumption that the key transcription regulatory pathways couple to distinct promoters in addition to the common CREB activation pathway. While the details of this separate coupling are not known, there is considerable evidence for such a mechanism, in the form of unique subsets of transcripts generated upon activation of different upstream pathways [26]–[29]. We represented this mechanism as an alternate model (Fig. 7D), where for simplicity CaMKIV, MAPK and PP1 each activated a distinct promoter, leading to mRNA synthesis. Using this model, we found that the different plasticity stimuli were transformed into distinct combinations of expression of different mRNA transcripts (Fig. 7E). Interestingly, the theta-burst stimulus which elicits robust LTP, caused relatively little activation of the subset of pathways in our model. Relatively low theta-burst activation of the CaMKII and PKA pathway has also been reported in another simulation study [56], and we suggest that pathways outside the scope of our model may be involved in the transcriptional response to theta-burst stimuli [81]. In the cell we expect considerably more complex promoter control signaling and hence still more varied mRNA outcomes [82]–[84]. Thus this set of simulations supports our hypothesis that the mRNA synthesis reactions, in conjunction with the different time-courses of stimulus-triggered pathways, may transform stimulus activity patterns into distinct profiles of mRNA expression.

### Simultaneous Input Patterns Sum Non-linearly to Give Rise to Novel Combinatorial mRNA Expression

The branching of a dendrite tree is complex and neurons receive numerous inputs from different synapses simultaneously or in succession. This input information must funnel into the soma to control transcription. This raises two questions: First, can the transcription control pathways distinguish between simultaneous inputs? Second, is the summation of inputs linear or nonlinear? To address these questions, we compared the mRNA outcome to the delivery of simultaneous, mutually opposed LTP and LTD stimuli (Fig. 7F). We modeled the combinatorial mRNA response to LTD plus different variants of LTP stimuli. We found that the predicted mRNA response pattern was different both from the individual responses (Fig. 7G), and from the sum of the individual responses (Fig. 7H). Specifically, the PP1-driven mRNA profile was close to a simple sum, but CaMKIV and MAPK mRNAs responded differently. Thus, the outcome of simultaneous inputs is that the transcriptional outcomes are indeed distinguishable from either input on its own, and the resultant mRNA profile is distinct, rather than an overlay of the outcomes of the individual patterns.

Overall, our final set of simulations shows that the mRNA transcriptional control network decodes a wide range of temporal stimuli implicated in synaptic plasticity, and generates distinct combinations of mRNA transcripts in response.

## Discussion

Selective transcription control is a cell-wide determinant of plasticity, and underlies spatially localized processes such as biochemical signaling, protein traffic, and protein synthesis. Transcriptional control is also crucial in many other biological contexts but there have been few mechanistic models of the molecular information processing leading from cellular inputs to the synthesis of mRNA. Many studies suggest that CREB-mediated transcription is essential for formation of long-term memory, but the signaling involved in this process is not fully understood. Here we built a biochemically detailed model of some key pathways in this signaling and parameterized these based on published experiments. We replicated a number of knockout experiments. The model predicts that even this limited subset of the transcriptional control network acts like a versatile decoder of stimulus patterns, and can generate diverse combinatorial mRNA expression patterns.

### Somatic Pattern Integration

The soma has a unique role in plasticity: there is just one soma to manage production of proteins for many thousands of synapses. To some extent this problem is mitigated by synapse-local protein synthesis. However, this too requires that appropriate sets of mRNA transcripts be available to the local synthesis machinery. Many questions remain about which fraction of proteins are made at the soma, and which at the synapse; how proteins and mRNAs ‘know’ which synapse to go to; which signals are important in synaptic tagging; and how the soma decides which mRNAs to make [85]. The current study addresses the last question.

How does synaptic information reach the soma? One possibility is that stimulus mediated activation of biochemical molecules is followed by transport of these signals from the potentiated synapse to the nucleus. There are two problems with this. First, the diffusion/transport process is slow. It would take 10 minutes for a signal to be transported from a spine 600 micron down the dendrite, even with a fast motor [86]. In contrast, transcription commences relatively quickly (within 2 min [24], [87], [88]). Second, the signal decays as it spreads along the dendrite and thus, the signal reaching the nucleus might not be sufficient for activating transcription. Local synaptic activity triggered biochemistry might be reasonable in the case where the synapses are located very close to the soma.

Another possibility is that distal synapses rely on electrical signaling to trigger chemical signaling in the dendritic shaft and soma [89]. Although the effect of a single synapse at the soma is small (typically under one mV, [90], [91]), simultaneous activation of many synapses may trigger somatic calcium influx through voltage-gated ion channels. Many of the pathways in this model are calcium-dependent. It is likely that both these mechanisms operate, depending on the location of synapses and the function of the synthesized mRNA. In our model we do not distinguish between these kinds of input. We instead assume that all the soma has to work with are patterns in time and contextual information such as BDNF. Our model suggests that these are sufficient to generate considerable transcript diversity. This observation is supported by the results of previously published experiments demonstrating input-dependent synthesis of specific mRNA [27]–[29].

One specific issue which our model addresses is the possibility that the soma may have to deal with different, perhaps contradictory forms of input from different dendritic branches. For example, one branch may be subjected to an LTP-, and another to an LTD-inducing stimulus. Surprisingly, the predicted outcome of such a combination is an entirely distinct pattern of mRNA transcription. The implication is that while the sub-branches may indeed get the required molecules for their stimulus-specific remodeling program, the proportions may change and they may also get additional transcripts to modulate the outcome. This may lead to very wide-ranging heterosynaptic interactions between dendrites undergoing different kinds of plasticity.

Overall, we see that the soma is capable of quite sophisticated decision-making based on temporal pattern and signaling context. The cellular transcription control network response is therefore far more nuanced than a simple stress-response to strong activation [20], [92].

### Other Layers of Decoding

Having generated a suitable combination of transcripts and proteins, how does the cell know where to send them, and how do the target synapses know what to do with them? Newly synthesized mRNAs are translated either near the nucleus [93], [94] or in the dendrites [95], [96]. Delivery of mRNAs or proteins to the potentiated synapse introduces altogether a new level of regulation in neuronal gene expression. Frey and Morris suggest that synaptic tagging helps direct mRNA or protein to particular synaptic sites [3]. Recently active synaptic sites are ‘tagged’, presumably through some persistent biochemical activity, leading to capture of newly synthesized mRNA and proteins.

Synapses themselves are versatile pattern-decoding machines [56], [97], [98]. This decoding can happen in two stages. First, patterned synaptic input sets up activity in distinct combinations of signaling pathways [27]. This forms a layer of specificity to recruitment and incorporation of subsets of mRNA and proteins, though the mechanisms for this are yet to be experimentally defined. Second, there are further sets of synaptic signaling pathways that control activity-dependent protein synthesis once mRNA has reached the synapse. These too are pattern-selective [31]. It is interesting that the ∼15 minute round-trip time from the synapse, to the soma, and transport of mRNA back to the synapse, overlaps with the predicted activation time-scale of the synaptic translational machinery [31].

These additional layers of control provide local control with distinct possible outcomes over thousands of synapses. In contrast, the somatic control of transcription is central: it decides what the rest of the cell has to work with. Hundreds of genes are transcribed in response to neuronal stimulation [16]. The current study provides an insight into how even a small subset of known biochemical control pathways can orchestrate them to produce diversity in cellular responses.

## Materials and Methods

We used the Kinetikit interface of GENESIS, the General Neuronal Simulation System [99] and MOOSE, the Multiscale Object-Oriented Simulation Environment [100] for running the simulations. GENESIS uses an explicit exponential Euler method and MOOSE uses an adaptive Runge-Kutta method. The complete model consisted of 142 molecules, 68 molecule-molecule interactions and 70 enzymatic reactions. The biochemical reactions and the parameters (418) used in the model are shown in Supporting material (Dataset S1). The GENESIS version of our reference (Dataset S2) and alternate model (Dataset S3) are also available in our supporting material and on the DOQCS database [43]. All the production simulations were performed in MOOSE for greater accuracy and speed, however the results from the GENESIS simulator match closely. The parameters were estimated from published data from pharmacological, genetic, and molecular biological experiments [101]. Hand tuning of the parameters were done to fit data of multiple published experiments. Once the model was constrained and validated, parameter sensitivity analysis was performed. Parameterization was substantially on the basis of published time-series experiments, which were converted using a screen capture and analysis program (Engauge Digitizer) into numerical values. These were tabulated and re-plotted for comparison with our own graphs for Fig. 2, 3 and 4.

Parameter sensitivity analysis was done by scaling the reference model parameters one at a time by factors of 0.1, 0.2, 0.3, 0.5, 0.8, 0.9, 1.1, 1.2, 1.5, 2, 3, 5, and 10 fold. The stimulus used was three pulses of Ca^2+^ presented with a 5 min spacing. Each pulse was of 2 *µ*M amplitude and 1 sec wide. The stimulus was delivered after the model reached the steady-state at 7000 sec. The concentration of the readouts (Total_pCaMKIV, Total_MAPK, pCREB-CRE and mRNA) were recorded at 1000 sec and 2000 sec after the stimulus. The obtained concentration was normalized by the original parameter model. These normalized fold change were plotted against logarithmic value of the parameter scale factor.

### Pathway Inhibition Simulations

In all these simulations we first ran the model for 7000 sec to reach steady-state. We then presented the stimulus. For measuring LTP responses, the stimulus was three pulses of Ca^2+^ influx, each 1 sec wide and 2 *µ*M amplitude separated by 300 sec. To measure the outcome of LTD stimuli, we delivered a steady Ca^2+^ stimulus of 0.2 *µ*M amplitude for 900 sec. To measure the dependence of CaMKIV, MAPK, CREB and TORC1 on mRNA synthesis for different inputs, we performed knockout experiments. We have set the concentration of knockout molecule at zero and measured the read-outs.

### Model Tuning to Interstimulus Interval

We ran the model to steady-state at 7000 sec, and then presented the stimulus (3 pulses of Ca^2+^ influx, each 1 second wide and 2 *µ*M amplitude) at the indicated inter-tetanic intervals. The pulses of Ca^2+^ were separated by 3, 10, 20, 300, 600, 900 and 1800 sec. We measured the change in read-out for 2 hr after the stimulus presentation. The trapezoidal rule was used to calculate area under the curve after the stimulus presentation.

### Differential Synthesis of mRNA from Different Stimulus Patterns

We used four distinct input stimuli for analyzing pattern-dependent differential synthesis of mRNA. These were: 1: spaced-LTP (3 pulses of Ca^2+^ influx, each 1 sec wide and 2 *µ*M amplitude separated by 300 sec); 2: massed-LTP (3 pulses of Ca^2+^ influx, each 1 sec wide and 2 *µ*M amplitude separated by 10 sec); 3: theta-burst (4 sets of stimuli delivered after every 20 sec where each set consisted of 10 calcium pulses of duration 75 ms and concentration 2 *µ*M, presented every 2 sec [56]) and 4: LTD (a steady Ca^2+^ stimulus of 0.2 *µ*M amplitude for 900 sec). To see the effect of different input on differential synthesis of mRNA, we extended our model so that the existing PP1, MAPK and CaMKIV pathways individually regulated mRNA synthesis. They did so by a binding reaction to a downstream molecule A, B and C respectively. Each of these binding steps was assumed to have the same kinetics. These molecules are a minimal representation of the machinery with which PP1, CaMKIV and MAPK interact, in order to direct synthesis of specific mRNA. We ran the model to steady-state at 7000 sec, and then presented the stimulus. The mRNA response at 20 min time-point after presenting the stimulus was used for plotting Fig. 7E.

### Differential Synthesis of mRNA from Simultaneous Inputs

The calcium concentration of simultaneous inputs were added and then presented as stimulus to an alternate model at 7000 sec. The mRNA response at 20 min time-point after presenting the stimulus was used for plotting Fig. 7G and 7H.

## Supporting Information

Dataset S1 **Parameter sources and notes.**
(PDF)

Dataset S2 **Model equations and parameters of our reference model.**
(PDF)

Dataset S3 **Model equations and parameters of our alternate model.**
(PDF)

File S1 **Supporting figures.** Figure S1. Diagram of existed signaling models incorporated into the current model. Figure S2. Chemical reaction diagram of MAPK pathway showing activation of MAPK by bRaf and cRaf. Figure S3. Phosphorylated MAPK as a function of time and chemical reaction diagram of submodels. Figure S4. Parameter sensitivity analysis for Total_pCaMKIV. Figure S5. Parameter sensitivity analysis for Total_MAPK. Figure S6. Parameter sensitivity analysis for pCREB_CRE. Figure S7. Sensitivity of mRNA synthesis rate for various calcium-input patterns. Figure S8. Total_pCaMKIV and pCREB-CRE as a function of Ca^2+^ level. Figure S9. Response of model to combinations of Ca^2+^ and BDNF stimulation.
(PDF)
