# Supplementary material for: Transcription Control Pathways Decode Patterned Synaptic Inputs into Diverse mRNA Expression Profiles
Source: PLoS One. 2014 May 1;9(5):e95154. doi: 10.1371/journal.pone.0095154 (PMC4006808; doi:10.1371/journal.pone.0095154)
Supplement: Dataset S1 — Parameter sources and notes. (PDF) [file pone.0095154.s001.pdf]

Parameters source and notes

CaMKIV signaling

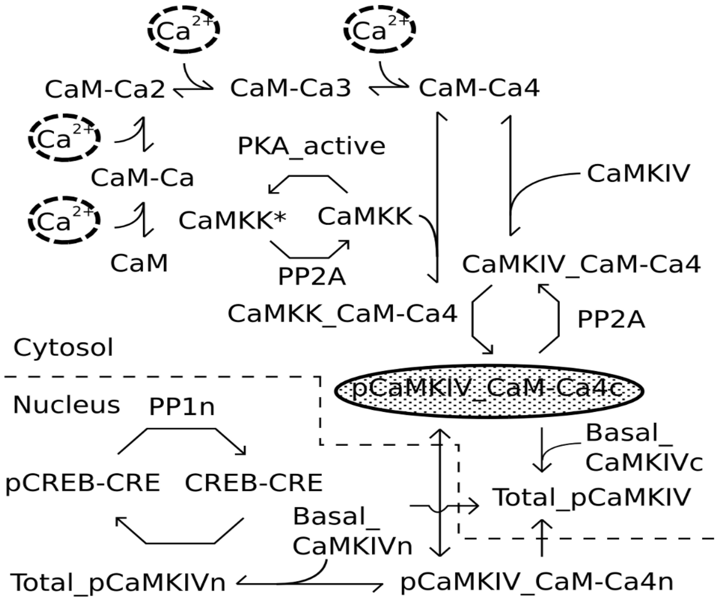

Molecule	Concentration (μM)	Buffered	Notes
CaMKK_cytosol	0.5	No	Calculated from purification data assuming 7x10^7 neurons/g (1).
PP2Ac	0.15	Yes	DOQCS
CaMKIVn	1	No	calculated from purification data assuming 7x10^7 neurons/g (2)
CREB-CRE	0.5	No	(3)

Basal_CaMKIVc	0.0005	No	It was set to obtain significant Ca2/CaM-independent CaM kinase IV activity (4).
Basal_CaMKIVn	0.00005	No	It was set to obtain significant Ca2/CaM-independent CaM kinase IV activity (4).

	Specification		Binding/Enzymatic Reaction	Rates	Notes
1)	Binding of Calmodulin-Calcium to CaMKIII		CaMKK_c + CaM-Ca4 <=> CaMKK_CaM-Ca4	Kf=4.05 /sec/µM, Kb=0.02 /sec	(5), (6)
2)	Binding of Calmodulin-Calcium to CaMKIV		CaMKIV + CaM-Ca4 <=> CaMKIV_CaM-Ca4	Kf=0.01332 /sec/µM, Kb=0.01 /sec	(5), (6)
3)	Phosphorylation of CaMKIV by CaMKK		CaMKIV_CaM-Ca4 + CaMKK_CaM-Ca4 -> pCaMKIV_CaM-Ca4c + CaMKK_CaM-Ca4	Km=1.3 µM, kcat=1.1 /sec	Assumed
4)	Dephosphorylation of pCaMKIV		pCaMKIV_CaM-Ca4c + PP2A -> CaMKIV_CaM-Ca4 + PP2A	Km=8.8 µM, kcat=2 /sec	The rates was used by PP2A for other substrates (DOQCS) so we tried the same.
5)	Transport of CaMKIV from cytosol to nucleus and vice versa		pCaMKIV_CaM-Ca4c <=> pCaMKIV_CaM-Ca4n	Kf=0.0009 /sec, Kb=0.007 /sec	It was set to obtain a slow transport.
6)	Transport of active PKA from cytosol to nucleus and vice versa		PKA-active <-> PKA-nucleus	Kf=0.000305 /sec, Kb=0.00125 /sec	(7)
7)	Phosphorylation of CaMKK by PKA-active		CaMKK + PKA-active -> CaMKK*	Km=4.699 µM, kcat=0.6833 /sec	BRENDA
8)	Dephosphorylation of CaMKK* by PKA-active		CaMKK* + PP2A -> CaMKK	Km=4.99 µM, kcat=0.7 /sec	BRENDA

9)	Sum total of nuclear and cytosolic CaMKIV		Basal_CaMKIVc + pCaMKIV_CaM-Ca4c + pCaMKIV_CaM-Ca4n + Basal_CaMKIVn -> Total_pCaMKIV		This reaction sums the level of Basal_CaMKIVc, pCaMKIV_CaM-Ca4c, pCaMKIV_CaM-Ca4n and Basal_CaMKIVn
10)	Sum total of CaMKIVn		pCaMKIV_CaM-Ca4n + Basal_CaMKIVn -> Total_pCaMKIVn		This reaction sums the level of pCaMKIV_CaM-Ca4n and Basal_CaMKIVn.
11)	Phosphorylation of CREB-CRE by Total_pCaMKIV		CREB-CRE + Total_CaMKIVn -> pCREB-CRE + Total_pCaMKIVn	Km=5.5 µM, kcat=0.7 /sec	(8)
12)	Dephosphorylation of pCREB-CRE by PP1n		pCREB-CRE + PP1n -> pCREB-CRE +PP1n	Km=5 µM, kcat=1 /sec	BRENDA

MAPK signaling

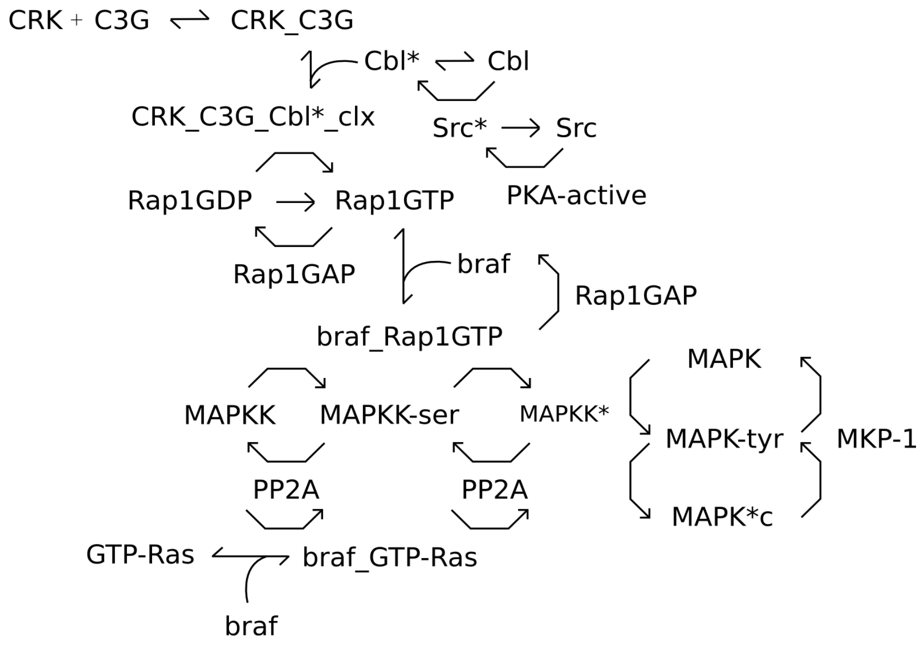

Molecule	Concentration (μM)	Buffered	Notes
CRK	1	No	(9)
C3G	0.5	No	(9)
Cbl	0.5	No	(9)
Src	0.02	No	Assumed
Rap-1GDP	0.2	No	(9)
Rap-1GAP	0.012	No	(9)
braf	0.2	No	(9)
MAPK	0.36	No	DOQCS
MKP-1	0.015	No	DOQCS

	Specification		Binding/Enzymatic Reaction	Rates	Notes
1)	Phosphorylation of Src by PKA_active		Src + PKA_active -> Src* + PKA_active	Km=0.049 μM, kcat=20 /sec	BRENDA
2)	Converstion of Src* to Src		Src* <=> Src	Kf=100 /sec , Kb=0.1 /sec	Assumed
3)	Phosphorylation of Cbl by Src*		Cbl + Src* -> Cbl* + Src*	Km=0.5 μM, kcat=40 /sec	BRENDA
4)	Converstion of Cbl* to Cbl		Cbl* <=> Cbl	Kf=10 /sec, Kb=0.01 /sec	Assumed
5)	Binding of CRK to C3G		CRK + C3G <=> CRK_C3G	Kf=1 /sec/μM, Kb=0.002 /sec	(9)
6)	Binding of CRK_C3G to Cbl*		CRK_C3G + Cbl* <=> CRK_C3G_Cbl*_clx	Kf=1 /sec/μM, Kb=0.2 /sec	CRK_C3G binds to other substrate with this affinity so we tried the same (9).
7)	Converstion of Rap1GDP to Rap1GTP by CRK_C3G_Cbl*_clx		Rap1GDP + CRK_C3G_Cbl*_clx -> Rap1GTP + CRK_C3G_Cbl*_clx	Km=0.0099 μM, kcat=0.2 /sec	(9)
8)	Converstion of Rap1GDP by Intrinsic GTPase		Rap1GTP <=> Rap1GDP	Kf=0.0001 /sec, Kb=0	(9)

9)	Hydrolysis of Rap1GTP by Rap1GAP		Rap1GTP + Rap1GAP -> Rap1GDP + Rap1GAP	Km=0.999 µM, kcat=2 /sec	(9)
10)	Binding of braf to Rap1GTP		Rap1GTP + braf <=> braf_Rap1GTP	Kf=60 /sec/µM, kb=0.5 /sec	(9)
11)	Binding of braf to GTP-Ras		GTP-Ras + braf <=> braf_GTP-Ras	Kf=60 /sec/µM, kb=0.5 /sec	(9)
12)	Degradation of braf_Rap1GTP by Rap1GAP		braf_Raf1GTP + Rap1GAP -> braf + Rap1GTP + Rap1GAP	Km=0.999 µM, kcat=2 /sec	(9)
13)	Phosphorylation of MAPKK by braf_Rap1GTP		MAPKK + braf_Rap1GTP -> MAPKK-ser + braf_Rap1GTP	Km=0.16 µM, kcat=0.3 /sec	(9)
14)	Phosphorylation of MAPKK-ser by braf_Rap1GTP		MAPKK-ser + braf_Rap1GTP -> MAPKK* + braf_Rap1GTP	Km=0.16 µM, kcat=0.3 /sec	(9)
15)	Phosphorylation of MAPKK by braf_GTP-Ras		MAPKK + braf_GTP-Ras -> MAPKK-ser + braf_GTP-Ras	Km=0.16 µM, kcat=0.2 /sec	(9)
16)	Phosphorylation of MAPKK-ser by braf_GTP-Ras		MAPKK-ser + braf_GTP-Ras -> MAPKK* + braf_GTP-Ras	Km=0.16 µM, kcat=0.2 /sec	(9)
17)	Dephosphorylation of MAPKK by PP2A		MAPKK-ser + PP2A -> MAPKK + PP2A	Km=15.657 µM, kcat=6 /sec	(9)
18)	Dephosphorylation of MAPKK by PP2A		MAPKK* + PP2A -> MAPKK-ser + PP2A	Km=15.657 µM, kcat=6 /sec	(9)
19)	Phosphorylation of MAPK by MAPKK*		MAPK + MAPKK* -> MAPK-tyr + MAPKK*	Km=0.046 µM, kcat=0.3 /sec	DOQCS
20)	Phosphorylation of MAPK-tyr by MAPKK*		MAPK-tyr + MAPKK* -> MAPK*C + MAPKK*	Km=0.046 µM, kcat=0.3 /sec	DOQCS
21)	Dephosphorylation of MAPK-tyr by MKP-1	.	MAPK-tyr + MKP-1 -> MAPK + MKP-1	Km=0.133 µM, kcat=4 /sec	DOQCS
22)	Dephosphorylation of MAPK-tyr by MKP-1		MAPK*c + MKP-1 -> MAPK-tyr + MKP-1	Km=0.133 µM, kcat=4 /sec	DOQCS

MAPKn signaling

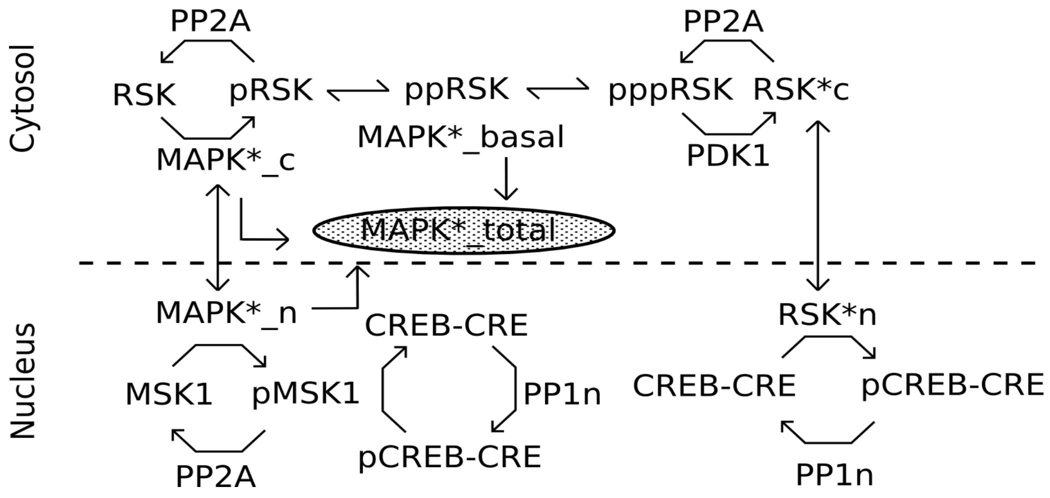

Molecule	Concentration (μM)	Buffered	Notes
RSK	0.2	No	Biomodels
PDK1	1	No	(10)
MSK1	0.2	No	Literature reports high expression of MSK1 in hippocampus (20).
CREB-CRE	0.5	No	In the basal state CREB is bound in a dimer form to the CAMP response element (CRE) sites of DNA (21) (3).
PP2An	0.1	No	Calculated from given N/C ratio (22). The concentarion of PP2Ac is taken from DOQCS.

	Specification		Binding/Enzymatic Reaction	Rates	Notes
1)	Phosphorylation of RSK by MAPK*_c		RSK + MAPK*_c -> pRSK + MAPK*_c	Km=5.3 µM, kcat=1.7 /sec	Biomodels
2)	Autophosphorylation of RSK		pRSK <=> ppRSK	Kf=0.1 /sec, Kb=10 /sec	Assumed
3)	Phosphorylation of ppRSK by PDK1		ppRSK + PDK1 -> RSK*c + PDK1	Km=10 µM, kcat=1 /sec	PDK1 have assigned this Km and kcat for different substrates but I tried for this too (10).
4)	Dephosphorylation of pRSK by PP2A		pRSK + PP2A -> RSK + PP2A	Km=8.8 µM, kcat=1 /sec	(10)
5)	Dephosphorylation of RSK*c by PP2A		RSK*c + PP2A -> ppRSK + PP2A	Km=8.8 µM, kcat=1 /sec	(10)
6)	Transport of MAPK*_c from cytosol to nucleus and vice versa		MAPK*_c <-> MAPK*n	Kf=0.0001 /sec, Kb=0.003 /sec	(11), (12)
7)	Phosphorylation of MSK1 by MAPK*_n		MSK1 + MAPK*_n -> pMSK1 + MAPK*_n	Km=5.3 µM, kcat=0.1 /sec	We used the same Km for MAPK as it is used by MAPK for RSK
8)	Dephosphorylation of pMSK1 by PP2A		pMSK1 + PP2A -> MSK1 + PP2A	Km=8.8 µM, kcat=1 /sec	(10)
9)	Phosphorylation of CREB-CRE by pMSK1		CREB-CRE + pMSK1 -> pCREB-CRE + pMSK1	Km=2 µM, kcat=0.1 /sec	MSK1 phosphorylates CREB with a Km lower than RSK1 and PKA (Km= 5 uM) (13), (14).
10)	Dephosphorylation of pCREB-CRE by PP1n		pCREB-CRE + PP1n -> pCREB-CRE +PP1n	Km=5 µM, kcat=1 /sec	DOQCS

11)	Transport of RSK*_c from cytosol to nucleus and vice versa		RSK*c <-> RSK*n	Kf = 0.001 /sec, Kb = 0.005 /sec	The rates are set to assign a slow transport.
12)	Phosphorylation of CREB-CRE by RSK*n		CREB-CRE + RSK*n -> pCREB-CRE + RSK*n	Km=5 µM, kcat=0.1 /sec	CREB is a poor substrate for RSK in compare to MSK. So, we have set the Km higher (14)
13)	Dephosphorylation of pCREB-CRE by PP1n		pCREB-CRE + PP1n -> pCREB-CRE +PP1n	Km=5 µM, kcat=1 /sec	DOQCS
14)	Sum total of active_MAPK		MAPK*_basal + MAPK*_c + MAPK*_n -> MAPK*_total		This reaction sums the level of MAPK*_basal, MAPK*_c and MAPK*_n

mRNA synthesis model

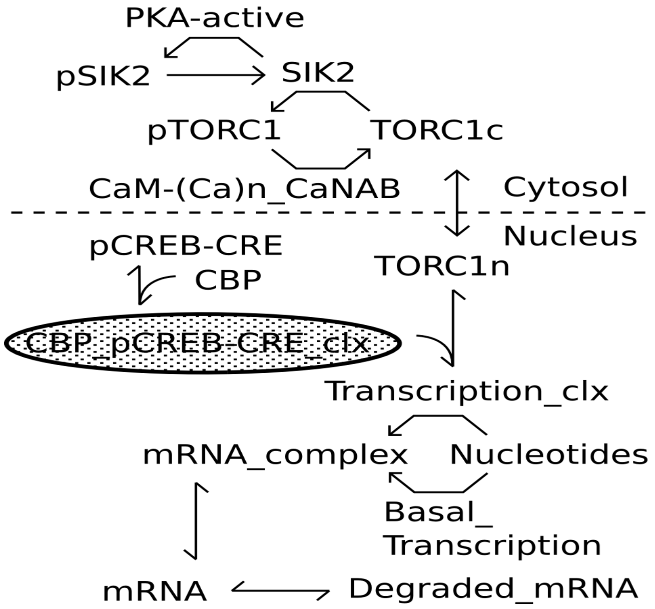

Molecule	Concentration (µM)	Buffered	Notes
SIK2	0.5	No	SIK2 is present in abundance in neurons (15).
TORC1c	0.1	No	TORC1 mRNA and protein is highly expressed in hippocampus (16)
CBP	0.5	No	Biomodels (2 =BIOMD0000000395)
Nucleotides	0.2	Yes	DOQCS
Basal_Transcription	0.00005	No	This is set on account of CREB independent mRNA synthesis.

	Specification		Binding/Enzymatic Reaction	Rates	Notes
1)	Phosphorylation of SIK2 by PKA-active		SIK2 + PKA-active -> pSIK2 + PKA-active	Km=4.56 µM, kcat=0.1 /sec	BRENDA
2)	Converstion of pSIK2		pSIK2 <=> SIK2	Kf=0.1 /sec, Kb=0	Assumed
3)	Phosphorylation of TORC1c by SIK2		TORC1c + SIK2 -> pTORC1 + SIK2	Km=4 µM, kcat=0.4 /sec	Assumed
4)	Dephosphorylation of pTORC1 by CaM-(Ca)n_CaNAB		pTORC1 + CaM-(Ca)n_CaNAB -> TORC1c + CaM-(Ca)n_CaNAB	Km=0.4 µM, kcat=0.1 /sec	BRENDA
5)	Transport of TORC1c from cytosol to nucleus and vice versa		TORC1c <-> TORC1n	Kf = 0.01 /sec, Kb= 0.001 /sec	Assumed but set in such a way to give a slow transport

6)	Binding of pCREB-CRE and CBP		pCREB-CRE + CBP <=> CBP_pCREB-CRE_clx	Kf = 0.114 /sec/μM, Kb= 0.025 /sec	constrained on the basis of this publication (17)
7)	Binding of CBP_pCREB_CRE_clx and TORC1n		CBP_pCREB_CRE_clx + TORC1n -> Transcription_clx	Kf = 1 /sec/μM, Kb =0.1 /sec	(18)
8)	mRNA synthesis		Nucleotides + Transcription_clx -> mRNA complex	Km=1.08 μM, kcat=0.05 /sec	We have estimated the parameters to obtain around 17 molecules/copies of mRNA per cell (19).
9)	Basal mRNA synthesis		Nucleotides + Basal_Transcription -> mRNA complex	Km=1.08 μM, kcat=0.05 /sec	We have estimated the parameters to obtain around 17 molecules/copies of mRNA per cell (19).
10)	mRNA elongation		mRNA_complex <=> mRNA	Kf = 1.44 /sec, Kb =0.0001 /sec	Assumed
11)	Degradation of mRNA		mRNA <=> Degraded_mRNA	Kf = 1 /sec, Kb =0	We assigned a generic degradation rate of 1/sec.

Supporting References

1. Okuno, S., T. Kitani, and H. Fujisawa. 1994. Purification and Characterization of Ca2+/Calmodulin-Dependent Protein Kinase IV Kinase from Rat Brain. J. Biochem. (Tokyo). 116: 923 –930.

2. Ohmstede, C.A., K.F. Jensen, and N.E. Sahyoun. 1989. Ca2+/calmodulin-dependent protein kinase enriched in cerebellar granule cells. Identification of a novel neuronal calmodulin-dependent protein kinase. J. Biol. Chem. 264: 5866–5875.

3. Mayr, B., and M. Montminy. 2001. Transcriptional regulation by the phosphorylation-dependent factor CREB. Nat. Rev. Mol. Cell Biol. 2: 599–609.

4. Selbert, M.A., K.A. Anderson, Q.-H. Huang, E.G. Goldstein, A.R. Means, et al. 1995. Phosphorylation and Activation of Ca-Calmodulin-dependent Protein Kinase IV by Ca-Calmodulin-dependent Protein Kinase Ia Kinase. J. Biol. Chem. 270: 17616 –17621.

5. Matsushita, M., and A.C. Nairn. 1998. Characterization of the mechanism of regulation of Ca2+/ calmodulin-dependent protein kinase I by calmodulin and by Ca2+/calmodulin-dependent protein kinase kinase. J. Biol. Chem. 273: 21473–21481.

6. Tokumitsu, H., and T.R. Soderling. 1996. Requirements for calcium and calmodulin in the calmodulin kinase activation cascade. J. Biol. Chem. 271: 5617–5622.

7. Harootunian, A.T., S.R. Adams, W. Wen, J.L. Meinkoth, S.S. Taylor, et al. 1993. Movement of the free catalytic subunit of cAMP-dependent protein kinase into and out of the nucleus can be explained by diffusion. Mol. Biol. Cell. 4: 993–1002.

8. Enslen, H., H. Tokumitsu, and T.R. Soderling. 1995. Phosphorylation of CREB by CaM-kinase IV activated by CaM-kinase IV kinase. Biochem. Biophys. Res. Commun. 207: 1038–1043.

9. Sasagawa, S., Y. Ozaki, K. Fujita, and S. Kuroda. 2005. Prediction and validation of the distinct dynamics of transient and sustained ERK activation. Nat. Cell Biol. 7: 365–373.

10. Jain, P., and U.S. Bhalla. 2009. Signaling logic of activity-triggered dendritic protein synthesis: an mTOR gate but not a feedback switch. Plos Comput. Biol. 5: e1000287.

11. Adachi, M., M. Fukuda, and E. Nishida. 1999. Two co-existing mechanisms for nuclear import of MAP kinase: passive diffusion of a monomer and active transport of a dimer. Embo J. 18: 5347–5358.

12. Fujioka, A., K. Terai, R.E. Itoh, K. Aoki, T. Nakamura, et al. 2006. Dynamics of the Ras/ERK MAPK cascade as monitored by fluorescent probes. J. Biol. Chem. 281: 8917–8926.

13. Roux, P.P., and J. Blenis. 2004. ERK and p38 MAPK-activated protein kinases: a family of protein kinases with diverse biological functions. Microbiol. Mol. Biol. Rev. Mmbr. 68: 320–344.

14. Deak, M., A.D. Clifton, L.M. Lucocq, and D.R. Alessi. 1998. Mitogen- and stress-activated protein kinase-1 (MSK1) is directly activated by MAPK and SAPK2/p38, and may mediate activation of CREB. Embo J. 17: 4426–4441.

15. Sasaki, T., H. Takemori, Y. Yagita, Y. Terasaki, T. Uebi, et al. 2011. SIK2 is a key regulator for neuronal survival after ischemia via TORC1-CREB. Neuron. 69: 106–119.

16. Zhou, Y., H. Wu, S. Li, Q. Chen, X.-W. Cheng, et al. 2006. Requirement of TORC1 for late-phase long-term potentiation in the hippocampus. Plos One. 1: e16.

17. Bito, H., K. Deisseroth, and R.W. Tsien. 1996. CREB phosphorylation and dephosphorylation: a Ca(2+)- and stimulus duration-dependent switch for hippocampal gene expression. Cell. 87: 1203–1214.

18. Luo, Q., K. Viste, J.C. Urday-Zaa, G. Senthil Kumar, W.-W. Tsai, et al. 2012. Mechanism of CREB recognition and coactivation by the CREB-regulated transcriptional coactivator CRTC2. Proc. Natl. Acad. Sci. U. S. A. 109: 20865–20870.

19. Schwanhäusser, B., D. Busse, N. Li, G. Dittmar, J. Schuchhardt, et al. 2011. Global quantification of mammalian gene expression control. Nature. 473: 337–342.

20. Chwang, W.B., J.S. Arthur, A. Schumacher, and J.D. Sweatt. 2007. The nuclear kinase mitogen- and stress-activated protein kinase 1 regulates hippocampal chromatin remodeling in memory formation. J. Neurosci. Off. J. Soc. Neurosci. 27: 12732–12742.

21. Riedel, G., and B. Platt. 2004. From Messengers to Molecules: Memories are Made of These. Springer.

22. Turowski, P., A. Fernandez, B. Favre, N.J. Lamb, and B.A. Hemmings. 1995. Differential methylation and altered conformation of cytoplasmic and nuclear forms of protein phosphatase 2A during cell cycle progression. J. Cell Biol. 129: 397–410.

Databases referred:

DOQCS	htttp://doqcs.ncbs.res.in
Biomodels	http://www.ebi.ac.uk/biomodels/
BRENDA	http://www.brenda-enzymes.info/
REACTOME	http://www.reactome.org/
