# Supplementary material for: Transcription Control Pathways Decode Patterned Synaptic Inputs into Diverse mRNA Expression Profiles
Source: PLoS One. 2014 May 1;9(5):e95154. doi: 10.1371/journal.pone.0095154 (PMC4006808; doi:10.1371/journal.pone.0095154)
Supplement: Dataset S3 — Model equations and parameters of our alternate model. (PDF) [file pone.0095154.s003.pdf]

Alternate model of mRNA synthesis model

The entire model was divided into sub-modules.The major signaling pathways in our model were the CaMKIV pathway, MAPK pathway and PP1 pathway. We constructed an alternate model in which each of PP1, MAPK and CaMKIV bind to a downstream molecule A, B and C respectively. These molecules are a minimal representation of the machinery to which PP1, CaMKIV and MAPK bind, in order to direct synthesis of specific mRNA. These models were merged with previously published models for CaM, BDNF input pathway, PP1 and PKA signaling inputs.

The following is a list of parameters (two enzyme parameters Michaelis constant (Km) and turnover number (kcat)), two reaction parameters (forward rate (kf) and backward rate (kb)), and the total concentrations of each molecule (CoInit)) were used to build-up the model. Concentration of few molecules are set as buffered (fixed concentration) shown by 1 and some are not buffered shown by 0 in the list.
Kf and Kb are the scaled rates of kf and kb respectively which depend on cellular volume. The Km, kcat depends on k1, k2, k3. The relation is:
Km = (k2 + k3)/k1
kcat = k3
ratio = k2/k3
Volume of dendrite = 1e-15 m^3 and volume of nucleus 3.7e-16 m^3
Time units: Seconds (sec)
Concentration units: Micromolar (uM)
The modules are shown as group. Few reactions which are part of group and not included in the description of module are shown at bottom.

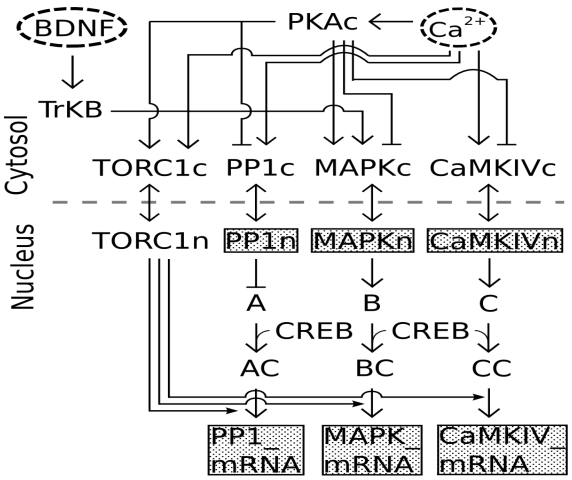

Concentration units: uM

Time units: sec

Default Volume (m^3) : 1e-15

Equations for group /kinetics

Reactions for group /kinetics

Reaction	kf	kb	Kf	Kb
Src_star <===> Src	100 s^-1	0.1 s^-1	100 s^-1	0.1 s^-1
Cbl_star <===> Cbl	10 s^-1	0.01 s^-1	10 s^-1	0.01 s^-1
C3G + CRK <===> CRK_C3G	1.6667e-06 #^-1.s^-1	0.002 s^-1	1 uM^-1.s^-1	0.002 s^-1
Rap1GTP <===> Rap1GDP	0.0001 s^-1	0 s^-1	0.0001 s^-1	0 s^-1
Rap1GTP + bRaf <===> bRaf_Rap1GTP	0.0001 #^-1.s^-1	0.5 s^-1	60 uM^-1.s^-1	0.5 s^-1
MAPK_star <===> MAPK_star_n	0.0001 s^-1	0.003 s^-1	0.0001 s^-1	0.003 s^-1
active_RSK2 <===> active_RSK2_n	0.001 s^-1	0.005 s^-1	0.001 s^-1	0.005 s^-1
pRSK <===> ppRSK	0.1 s^-1	10 s^-1	0.1 s^-1	10 s^-1
Ca_input <===> Ca	100 s^-1	100 s^-1	100 s^-1	100 s^-1
Grb2 + Sos <===> Sos.Grb2	4.1667e-07 #^-1.s^-1	0.0168 s^-1	0.25 uM^-1.s^-1	0.0168 s^-1

Sos.Grb2 + Shc_star <===> Shc_star.Sos.Grb2	8.3333e-06 #^-1.s^-1	0.1 s^-1	5 uM^-1.s^-1	0.1 s^-1
Sos_star <===> Sos	0.001 s^-1	0.1 s^-1	0.001 s^-1	0.1 s^-1
CBP + MAPKstar_CREB <===> CBP_pCREB_CRE	5.1351e-07 #^-1.s^-1	0.025 s^-1	0.114 uM^-1.s^-1	0.025 s^-1
mRNA_clx <===> mRNA	1.44 s^-1	0.0001 s^-1	1.44 s^-1	0.0001 s^-1
mRNA <===> degraded_mRNA	1 s^-1	0 s^-1	1 s^-1	0 s^-1
Cbl_star + CRK_C3G <===> CRK_C3G_Cbl_star_clx	1.6667e-06 #^-1.s^-1	0.2 s^-1	1 uM^-1.s^-1	0.2 s^-1
Grb2 + Sos_star <===> Sos_star.Grb2	4.1667e-08 #^-1.s^-1	0.0168 s^-1	0.025 uM^-1.s^-1	0.0168 s^-1
TORC1c <===> TORC1n	0.01 s^-1	0.001 s^-1	0.01 s^-1	0.001 s^-1
TORC1n + CBP_pCREB_CRE <===> Transcription_clx	4.5045e-06 #^-1.s^-1	0.1 s^-1	1 uM^-1.s^-1	0.1 s^-1
SIK2_star <===> SIK2	0.1 s^-1	0 s^-1	0.1 s^-1	0 s^-1
PP1-I1n <===> PP1_active_n + I1n	1 s^-1	9.009e-06 #^-1.s^-1	1 s^-1	2 uM^-1.s^-1
PP1-I1 <===> PP1-I1n	0.003 s^-1	0.003 s^-1	0.003 s^-1	0.003 s^-1
PP1_active_n + I1n <===> PP1-I1n	2.2521e-06 #^-1.s^-1	0.1 s^-1	0.49997 uM^-1.s^-1	0.1 s^-1
Shc_star <===> Shc	0.2 s^-1	0 s^-1	0.2 s^-1	0 s^-1
CBP + CaMKIVstar_CREB <===> CBP_pCREB_CaMKIV	5.1351e-07 #^-1.s^-1	0.025 s^-1	0.114 uM^-1.s^-1	0.025 s^-1
CBP_pCREB_CaMKIV + TORC1n <===> Transcription_Clx_	4.5045e-06 #^-1.s^-1	0.1 s^-1	1 uM^-1.s^-1	0.1 s^-1
mRNA_clx_CaMKIV <===> mRNA_CaMKIV	1.44 s^-1	0.0001 s^-1	1.44 s^-1	0.0001 s^-1
mRNA_CaMKIV <===> deg_mRNA_CaMKIV	1 s^-1	0 s^-1	1 s^-1	0 s^-1
A_star + CREB <===> PP1star_CREB	4.5045e-06 #^-1.s^-1	0.1 s^-1	1 uM^-1.s^-1	0.1 s^-1
CREB + B_star <===> MAPKstar_CREB	4.5045e-06 #^-1.s^-1	0.1 s^-1	1 uM^-1.s^-1	0.1 s^-1
C_star + CREB <===> CaMKIVstar_CREB	4.5045e-06 #^-1.s^-1	0.1 s^-1	1 uM^-1.s^-1	0.1 s^-1
CBP + PP1star_CREB <===> CBP_pCREB_PP1	5.1351e-07 #^-1.s^-1	0.025 s^-1	0.114 uM^-1.s^-1	0.025 s^-1
CBP_pCREB_PP1 + TORC1n <===> Transcr_clx_PP1	4.5047e-06 #^-1.s^-1	0.1 s^-1	1 uM^-1.s^-1	0.1 s^-1
PP1mRNA_clx <===> PP1_mRNA	1.44 s^-1	0.0001 s^-1	1.44 s^-1	0.0001 s^-1
PP1_mRNA <===> deg_PP1mRNA	1 s^-1	0 s^-1	1 s^-1	0 s^-1

Enzymes for group /kinetics

Enzyme-reaction	k1	k2	k3	Km	kcat	ratio
AC2 ---PKC-active--> AC2_star	1e-06 #^-1.s^-1	16 s^-1	4 s^-1	33.333 uM	4 s^-1	4
GAP ---PKC-active--> GAP_star	1e-05 #^-1.s^-1	16 s^-1	4 s^-1	3.3333 uM	4 s^-1	4
inact-GEF ---PKC-active--> GEF_star	1e-05 #^-1.s^-1	16 s^-1	4 s^-1	3.3333 uM	4 s^-1	4
craf-1 ---PKC-active--> craf-1_star	4.9999e-07 #^-1.s^-1	16 s^-1	4 s^-1	66.668 uM	4 s^-1	4
cAMP-PDE ---PKA-active--> cAMP-PDE_star	1e-05 #^-1.s^-1	36 s^-1	9 s^-1	7.5 uM	9 s^-1	4
Src ---PKA-active--> Src_star	0.0033334 #^-1.s^-1	80 s^-1	20 s^-1	0.049999 uM	20 s^-1	4
inact-GEF ---PKA-active--> inact-GEF_star	1e-05 #^-1.s^-1	36 s^-1	9 s^-1	7.5 uM	9 s^-1	4
I1 ---PKA-active--> I1_star	1e-05 #^-1.s^-1	36 s^-1	9 s^-1	7.5 uM	9 s^-1	4
CaMKK_CaM_Ca_c ---PKA-active--> CaM-Ca4 + CaMKKp	1.2116e-06 #^-1.s^-1	2.7333 s^-1	0.68333 s^-1	4.6999 uM	0.68333 s^-1	4
SIK2 ---PKA-active--> SIK2_star	1.8117e-07 #^-1.s^-1	0.4 s^-1	0.1 s^-1	4.5997 uM	0.1 s^-1	4
I1n ---PKA-active_n--> I1_star_n	2.7027e-05 #^-1.s^-1	36 s^-1	9 s^-1	7.5 uM	9 s^-1	4
bRaf_Rap1GTP ---Rap1GAP--> Rap1GDP + bRaf	0.00033667 #^-1.s^-1	200 s^-1	2 s^-1	0.99999 uM	2 s^-1	100
Rap1GTP ---Rap1GAP--> Rap1GDP	0.00033667 #^-1.s^-1	200 s^-1	2 s^-1	0.99999 uM	2 s^-1	100
MAPKK-ser ---bRaf_Rap1GTP--> MAPKK_star	1.5625e-05 #^-1.s^-1	1.2 s^-1	0.3 s^-1	0.16 uM	0.3 s^-1	4
MAPKK ---bRaf_Rap1GTP--> MAPKK-ser	1.5625e-05 #^-1.s^-1	1.2 s^-1	0.3 s^-1	0.16 uM	0.3 s^-1	4
MAPK-tyr ---MKP-1--> MAPK	0.00025 #^-1.s^-1	16 s^-1	4 s^-1	0.13333 uM	4 s^-1	4
MAPK_star ---MKP-1--> MAPK-tyr	0.00025 #^-1.s^-1	16 s^-1	4 s^-1	0.13333 uM	4 s^-1	4
craf-1_star ---PPhosphatase2A--> craf-1	3.1935e-06 #^-1.s^-1	24 s^-1	6 s^-1	15.657 uM	6 s^-1	4
MAPKK_star ---PPhosphatase2A--> MAPKK-ser	3.1935e-06 #^-1.s^-1	24 s^-1	6 s^-1	15.657 uM	6 s^-1	4
MAPKK-ser ---PPhosphatase2A--> MAPKK	3.1935e-06 #^-1.s^-1	24 s^-1	6 s^-1	15.657 uM	6 s^-1	4
craf-1_star_star ---PPhosphatase2A--> craf-1_star	3.1935e-06 #^-1.s^-1	24 s^-1	6 s^-1	15.657 uM	6 s^-1	4
ppRSK ---PDK1--> active_RSK2	8.3333e-07 #^-1.s^-1	4 s^-1	1 s^-1	10 uM	1 s^-1	4
active_RSK2 ---PP2A--> ppRSK	9.4692e-07 #^-1.s^-1	4 s^-1	1 s^-1	8.8005 uM	1 s^-1	4
I1_star ---PP2A--> I1	6.6e-06 #^-1.s^-1	25 s^-1	6 s^-1	7.8283 uM	6 s^-1	4.1667
CaMKKp ---PP2A--> CaMKK_c	1.1667e-06 #^-1.s^-1	2.8 s^-1	0.7 s^-1	4.9999 uM	0.7 s^-1	4
pCaMKIV_CaM_Ca_c ---PP2A--> CaMKIV_CaM_Ca_c	1.8939e-06 #^-1.s^-1	8 s^-1	2 s^-1	8.8002 uM	2 s^-1	4
pRSK ---PP2A--> RSK	9.4692e-07 #^-1.s^-1	4 s^-1	1 s^-1	8.8005 uM	1 s^-1	4

PP1-I1_star ---PP2A--> PP1-I1	6.6e-06 #^-1.s^-1	25 s^-1	6 s^-1	7.8283 uM	6 s^-1	4.1667
A_star ---PP1_active_n--> A	2.2523e-06 #^-1.s^-1	0.4 s^-1	0.1 s^-1	0.99998 uM	0.1 s^-1	4
B ---active_RSK2_n--> B_star	4.5045e-07 #^-1.s^-1	0.4 s^-1	0.1 s^-1	5 uM	0.1 s^-1	4
I1_star ---CaNAB-Ca4--> I1	5.7e-08 #^-1.s^-1	0.136 s^-1	0.034 s^-1	4.9708 uM	0.034 s^-1	4
B ---pMSK1_n--> B_star	1.1261e-06 #^-1.s^-1	0.4 s^-1	0.1 s^-1	2 uM	0.1 s^-1	4
pMSK1_n ---PP2An--> MSK1_n	2.5594e-06 #^-1.s^-1	4 s^-1	1 s^-1	8.7999 uM	1 s^-1	4
I1_star_n ---PP2An--> I1n	1.7262e-05 #^-1.s^-1	24 s^-1	6 s^-1	7.8285 uM	6 s^-1	4
PLC_g ---PLCg_basal--> PLC_g_star	1.3889e-05 #^-1.s^-1	2 s^-1	0.5 s^-1	0.3 uM	0.5 s^-1	4
Nucleotides ---Basal_transcription--> mRNA_clx	1.0416e-06 #^-1.s^-1	0.2 s^-1	0.05 s^-1	1.0812 uM	0.05 s^-1	4
Nucleotides ---Basal_transcription--> mRNA_clx_CaMKIV	1.0427e-06 #^-1.s^-1	0.2 s^-1	0.05 s^-1	1.08 uM	0.05 s^-1	4
Nucleotides ---Basal_transcription--> PP1mRNA_clx	1.0427e-06 #^-1.s^-1	0.2 s^-1	0.05 s^-1	1.08 uM	0.05 s^-1	4
C ---pCaMKIV_CaM_Ca_nuc--> C_star	2.8665e-06 #^-1.s^-1	2.8 s^-1	0.7 s^-1	5.5 uM	0.7 s^-1	4
GDP-Ras ---Shc_star.Sos.Grb2--> GTP-Ras	3.3e-05 #^-1.s^-1	0.8 s^-1	0.2 s^-1	0.050505 uM	0.2 s^-1	4
Rap1GDP ---CRK_C3G_Cbl_star_clx--> Rap1GTP	6.6668e-05 #^-1.s^-1	0.2 s^-1	0.2 s^-1	0.0099998 uM	0.2 s^-1	1
Cbl ---Src_star--> Cbl_star	0.00066665 #^-1.s^-1	160 s^-1	40 s^-1	0.50001 uM	40 s^-1	4
MSK1_n ---MAPK_star_n--> pMSK1_n	4.2496e-07 #^-1.s^-1	0.4 s^-1	0.1 s^-1	5.2999 uM	0.1 s^-1	4
Sos ---MAPK_star--> Sos_star	3.2552e-05 #^-1.s^-1	40 s^-1	10 s^-1	2.56 uM	10 s^-1	4
RSK ---MAPK_star--> pRSK	2.673e-06 #^-1.s^-1	6.8 s^-1	1.7 s^-1	5.2999 uM	1.7 s^-1	4
craf-1_star ---MAPK_star--> craf-1_star_star	3.25e-06 #^-1.s^-1	40 s^-1	10 s^-1	25.641 uM	10 s^-1	4
Nucleotides ---Transcription_clx--> mRNA_clx	1.0427e-06 #^-1.s^-1	0.2 s^-1	0.05 s^-1	1.08 uM	0.05 s^-1	4
TORC1c ---SIK2--> pTORC1	8.3333e-07 #^-1.s^-1	1.6 s^-1	0.4 s^-1	4 uM	0.4 s^-1	4
I1_star ---CaM_Ca_n-CaNAB--> I1	5.7e-07 #^-1.s^-1	1.36 s^-1	0.34 s^-1	4.9708 uM	0.34 s^-1	4
pTORC1 ---CaM_Ca_n-CaNAB--> TORC1c	2.0833e-06 #^-1.s^-1	0.4 s^-1	0.1 s^-1	0.40001 uM	0.1 s^-1	4
PP1-I1_star ---CaM_Ca_n-CaNAB--> PP1-I1	5.7e-07 #^-1.s^-1	1.36 s^-1	0.34 s^-1	4.9708 uM	0.34 s^-1	4
Nucleotides ---Transcription_Clx_C--> mRNA_clx_CaMKIV	1.0427e-06 #^-1.s^-1	0.2 s^-1	0.05 s^-1	1.08 uM	0.05 s^-1	4
A ---A_kinase--> A_star	2.2524e-06 #^-1.s^-1	0.4 s^-1	0.1 s^-1	0.99998 uM	0.1 s^-1	4
B_star ---B_phosphatase--> B	2.2523e-06 #^-1.s^-1	0.4 s^-1	0.1 s^-1	0.99998 uM	0.1 s^-1	4
C_star ---C_phospatase--> C	2.2524e-06 #^-1.s^-1	0.4 s^-1	0.1 s^-1	0.99998 uM	0.1 s^-1	4
Nucleotides ---Transcr_clx_PP1--> PP1mRNA_clx	1.0427e-06 #^-1.s^-1	0.2 s^-1	0.05 s^-1	1.08 uM	0.05 s^-1	4

Pools for group /kinetics

name	InitialConc	buffered	Volume
CaM-Ca4	0 uM	0	1000 fl
PKC-active	0.01 uM	1	1000 fl
PKA-active	0 uM	0	1000 fl
cAMP	0 uM	0	1000 fl
PKA-active_n	0 uM	0	370 fl
Ca	0.08 uM	0	1000 fl
Src	0.02 uM	0	1000 fl
Cbl	0.5 uM	0	1000 fl
C3G	0.5 uM	0	1000 fl
CRK	1 uM	0	1000 fl
CRK_C3G	0 uM	0	1000 fl
Rap1GTP	0 uM	0	1000 fl
Rap1GDP	0.2 uM	0	1000 fl
Rap1GAP	0.012 uM	0	1000 fl
bRaf_Rap1GTP	0 uM	0	1000 fl
bRaf	0.2 uM	0	1000 fl
MKP-1	0.015 uM	0	1000 fl
PPhosphatase2A	1 uM	0	1000 fl
MSK1_n	0.2 uM	0	370 fl
PDK1	1 uM	0	1000 fl
PP2A	0.15 uM	1	1000 fl
PP1_active_n	0 uM	0	370 fl
ppRSK	0 uM	0	1000 fl
active_RSK2	0 uM	0	1000 fl

active_RSK2_n	0 uM	0	370 fl
RSK	0.2 uM	0	1000 fl
pRSK	0 uM	0	1000 fl
BetaGamma	0 uM	0	1000 fl
CaNAB-Ca4	0 uM	0	1000 fl
PP1-active_c	1.8 uM	0	1000 fl
pMSK1_n	0 uM	0	370 fl
Ca_input	0 uM	0	1000 fl
PP2An	0.1 uM	0	370 fl
BDNF	5e-05 uM	1	999.97 fl
PLC_g	0.1 uM	0	1000 fl
PLCg_basal	0.0007 uM	0	1000 fl
Grb2	1 uM	0	1000 fl
Shc	0.5 uM	0	1000 fl
Sos	0.1 uM	0	1000 fl
Sos.Grb2	0 uM	0	1000 fl
CBP	0.5 uM	0	370 fl
mRNA_clx	0 uM	0	370 fl
Basal_transcription	5e-05 uM	0	370 fl
mRNA	0 uM	0	370 fl
degraded_mRNA	0 uM	1	370 fl
Basal_CaMKIV	0.0005 uM	0	1000 fl
pCaMKIV_CaM_Ca_nuc	0 uM	0	370 fl
pCaMKIV_CaM_Ca_n	0 uM	0	370 fl
Basal_CaMKIV_n	5e-05 uM	0	370 fl
Sum_total_CaMKIV	0 uM	0	1000 fl
MAPK_active_total	0 uM	0	1000 fl
Basal_MAPK_active	0.0001 uM	0	1000 fl
Shc_star	0 uM	0	1000 fl
PLC_g_star	0 uM	0	1000 fl
Sos_star	0 uM	0	1000 fl
Shc_star.Sos.Grb2	0 uM	0	1000 fl
Sos_star.Grb2	0 uM	0	1000 fl
CRK_C3G_Cbl_star_clx	0 uM	0	1000 fl
Cbl_star	0 uM	0	1000 fl
Src_star	0 uM	0	1000 fl
MAPK_star_n	0 uM	0	370 fl
MAPK_star	0 uM	0	1000 fl
pTORC1	0 uM	0	1000 fl
TORC1n	0 uM	0	370 fl
TORC1c	0.1 uM	0	1000 fl
CBP_pCREB_CRE	0 uM	0	370 fl
Transcription_clx	0 uM	0	370 fl
Nucleotides	0.2 uM	1	369.98 fl
SIK2	0.5 uM	0	1000 fl
SIK2_star	0 uM	0	1000 fl
PP1-I1n	0 uM	0	370 fl
I1n	0 uM	0	370 fl
I1_star_n	0 uM	0	369.98 fl
CaM_Ca_n-CaNAB	0 uM	0	1000 fl
CBP_pCREB_CaMKIV	0 uM	0	370 fl
Transcription_Clx_C	0 uM	0	370 fl
mRNA_clx_CaMKIV	0 uM	0	370 fl
mRNA_CaMKIV	0 uM	0	370 fl
deg_mRNA_CaMKIV	0 uM	1	370 fl
B	0.1 uM	0	370 fl

B_star	0 uM	0	370 fl
C	0.1 uM	0	370 fl
C_star	0 uM	0	370 fl
A_star	0 uM	0	370 fl
A	0.1 uM	0	369.98 fl
A_kinase	0.1 uM	0	369.98 fl
CREB	0.5 uM	0	370 fl
B_phosphatase	0.1 uM	0	370 fl
C_phospatase	0.1 uM	0	369.98 fl
MAPKstar_CREB	0 uM	0	370 fl
CaMKIVstar_CREB	0 uM	0	370 fl
PP1star_CREB	0 uM	0	370 fl
CBP_pCREB_PP1	0 uM	0	369.98 fl
Transcr_clx_PP1	0 uM	0	370 fl
PP1mRNA_clx	0 uM	0	370 fl
PP1_mRNA	0 uM	0	370 fl
deg_PP1mRNA	0 uM	1	370 fl
total_PP1_acive	0 uM	0	1000 fl

Equations for group /kinetics/PKA

Reactions for group /kinetics/PKA

Reaction	kf	kb	Kf	Kb
R2C2 + cAMP <===> R2C2-cAMP	9e-05 #^-1.s^-1	33 s^-1	54 uM^-1.s^-1	33 s^-1
R2C2-cAMP + cAMP <===> R2C2-cAMP2	9e-05 #^-1.s^-1	33 s^-1	54 uM^-1.s^-1	33 s^-1
R2C2-cAMP2 + cAMP <===> R2C2-cAMP3	0.000125 #^-1.s^-1	110 s^-1	75 uM^-1.s^-1	110 s^-1
cAMP + R2C2-cAMP3 <===> R2C2-cAMP4	0.000125 #^-1.s^-1	32.5 s^-1	75 uM^-1.s^-1	32.5 s^-1
R2C2-cAMP4 <===> PKA-active + R2C-cAMP4	60 s^-1	3e-05 #^-1.s^-1	60 s^-1	18 uM^-1.s^-1
R2C-cAMP4 <===> PKA-active + R2-cAMP4	60 s^-1	3e-05 #^-1.s^-1	60 s^-1	18 uM^-1.s^-1
R2-cAMP4 <===> R2 + cAMP	0.0005 s^-1	1.6667e-10 #^-1.s^	0.0005 s^-1	0.0001 uM^-1.s^-1
PKA-active + R2 <===> R2C1	0.0013317 #^-1.s^-1	0.186 s^-1	799.02 uM^-1.s^-1	0.186 s^-1
PKA-active + R2C1 <===> R2C2	0.0013317 #^-1.s^-1	0.186 s^-1	799.02 uM^-1.s^-1	0.186 s^-1
PKA-active + PKA-inhibitor <===> inhibited-PKA	0.0001 #^-1.s^-1	1 s^-1	60 uM^-1.s^-1	1 s^-1
PKA-active <===> PKA-active_n	0.000305 s^-1	0.00125 s^-1	0.000305 s^-1	0.00125 s^-1

Pools for group /kinetics/PKA

name	InitialConc	buffered	Volume
R2C2	0.5 uM	0	1000 fl
R2C2-cAMP	0 uM	0	1000 fl
R2C2-cAMP2	0 uM	0	1000 fl
R2C2-cAMP3	0 uM	0	1000 fl
R2C2-cAMP4	0 uM	0	1000 fl
R2C-cAMP4	0 uM	0	1000 fl
R2-cAMP4	0 uM	0	1000 fl
R2	0 uM	0	1000 fl
R2C1	0 uM	0	1000 fl
inhibited-PKA	0 uM	0	1000 fl
PKA-inhibitor	0.25 uM	0	1000 fl

Equations for group /kinetics/AC

Reactions for group /kinetics/AC

Reaction	kf	kb	Kf	Kb
CaM-Ca4 + AC1 <===> AC1-CaM	8.3333e-05 #^-1.s^-1	1 s^-1	50 uM^-1.s^-1	1 s^-1
AC2_star <===> AC2	0.1 s^-1	0 s^-1	0.1 s^-1	0 s^-1
cAMP-PDE_star <===> cAMP-PDE	0.1 s^-1	0 s^-1	0.1 s^-1	0 s^-1
PDE1 + CaM-Ca4 <===> CaM.PDE1	0.0012 #^-1.s^-1	5 s^-1	720 uM^-1.s^-1	5 s^-1

Enzymes for group /kinetics/AC

Enzyme-reaction	k1	k2	k3	Km	kcat	ratio
ATP ---AC1-CaM--> cAMP	7.5e-06 #^-1.s^-1	72 s^-1	18 s^-1	20 uM	18 s^-1	4
cAMP ---cAMP-PDE--> AMP	4.2e-06 #^-1.s^-1	40 s^-1	10 s^-1	19.841 uM	10 s^-1	4
cAMP ---PDE1--> AMP	3.5e-07 #^-1.s^-1	6.67 s^-1	1.667 s^-1	39.7 uM	1.667 s^-1	4.0012
cAMP ---CaM.PDE1--> AMP	2.1e-06 #^-1.s^-1	40 s^-1	10 s^-1	39.683 uM	10 s^-1	4
ATP ---AC2_star--> cAMP	2.9e-06 #^-1.s^-1	28 s^-1	7 s^-1	20.115 uM	7 s^-1	4
cAMP ---cAMP-PDE_star--> AMP	8.4e-06 #^-1.s^-1	80 s^-1	20 s^-1	19.841 uM	20 s^-1	4

Pools for group /kinetics/AC

name	InitialConc	buffered	Volume
ATP	5000 uM	1	1000 fl
AC1-CaM	0 uM	0	1000 fl
AC1	0.02 uM	0	1000 fl
AC2	0.015 uM	0	1000 fl
AMP	3.2549e+05 uM	0	0.0016667 fl
cAMP-PDE	0.45 uM	0	1000 fl
PDE1	2 uM	0	1000 fl
CaM.PDE1	0 uM	0	1000 fl
AC2_star	0 uM	0	1000 fl
cAMP-PDE_star	0 uM	0	1000 fl

Equations for group /kinetics/CaM

Reactions for group /kinetics/CaM

Reaction	kf	kb	Kf	Kb
CaM-Ca3 + Ca <===> CaM-Ca4	7.7501e-07 #^-1.s^-1	10 s^-1	0.46501 uM^-1.s^-1	10 s^-1
CaM + Ca <===> CaM-Ca	1.4141e-05 #^-1.s^-1	8.4853 s^-1	8.4846 uM^-1.s^-1	8.4853 s^-1
CaM-Ca2 + Ca <===> CaM-Ca3	6.0001e-06 #^-1.s^-1	10 s^-1	3.6001 uM^-1.s^-1	10 s^-1
CaM-Ca + Ca <===> CaM-Ca2	1.4141e-05 #^-1.s^-1	8.4853 s^-1	8.4846 uM^-1.s^-1	8.4853 s^-1

Pools for group /kinetics/CaM

name	InitialConc	buffered	Volume
CaM	20 uM	0	1000 fl
CaM-Ca3	0 uM	0	1000 fl
CaM-Ca2	0 uM	0	1000 fl
CaM-Ca	0 uM	0	1000 fl

Equations for group /kinetics/creb

Equations for group /kinetics/MAPK

Reactions for group /kinetics/MAPK

Reaction	kf	kb	Kf	Kb
craf-1 + GTP-Ras <===> Raf-GTP-Ras	1e-05 #^-1.s^-1	1 s^-1	6 uM^-1.s^-1	1 s^-1
craf-1_star + GTP-Ras <===> Raf_star-GTP-Ras	1.6666e-05 #^-1.s^-1	0.5 s^-1	9.9996 uM^-1.s^-1	0.5 s^-1
bRaf + GTP-Ras <===> braf-GTP-Ras	0.0001 #^-1.s^-1	0.5 s^-1	60 uM^-1.s^-1	0.5 s^-1

Enzymes for group /kinetics/MAPK

Enzyme-reaction	k1	k2	k3	Km	kcat	ratio
MAPKK ---Raf-GTP-Ras--> MAPKK-ser	1.5714e-05 #^-1.s^-1	1.2 s^-1	0.3 s^-1	0.15909 uM	0.3 s^-1	4
MAPKK-ser ---Raf-GTP-Ras--> MAPKK_star	1.5714e-05 #^-1.s^-1	1.2 s^-1	0.3 s^-1	0.15909 uM	0.3 s^-1	4
MAPKK-ser ---braf-GTP-Ras--> MAPKK_star	1.0417e-05 #^-1.s^-1	0.8 s^-1	0.2 s^-1	0.15999 uM	0.2 s^-1	4
MAPKK ---braf-GTP-Ras--> MAPKK-ser	1.0417e-05 #^-1.s^-1	0.8 s^-1	0.2 s^-1	0.15999 uM	0.2 s^-1	4
MAPKK-ser ---Raf_star-GTP-Ras--> MAPKK_star	1.5714e-05 #^-1.s^-1	1.2 s^-1	0.3 s^-1	0.15909 uM	0.3 s^-1	4
MAPKK ---Raf_star-GTP-Ras--> MAPKK-ser	1.5714e-05 #^-1.s^-1	1.2 s^-1	0.3 s^-1	0.15909 uM	0.3 s^-1	4
MAPK ---MAPKK_star--> MAPK-tyr	5.4e-05 #^-1.s^-1	1.2 s^-1	0.3 s^-1	0.046296 uM	0.3 s^-1	4
MAPK-tyr ---MAPKK_star--> MAPK_star	5.4e-05 #^-1.s^-1	1.2 s^-1	0.3 s^-1	0.046296 uM	0.3 s^-1	4

Pools for group /kinetics/MAPK

name	InitialConc	buffered	Volume
craf-1	0.2 uM	0	1000 fl
MAPKK	0.18 uM	0	1000 fl
MAPK	0.36 uM	0	1000 fl
MAPK-tyr	0 uM	0	1000 fl
MAPKK-ser	0 uM	0	1000 fl
Raf-GTP-Ras	0 uM	0	1000 fl
braf-GTP-Ras	0 uM	0	1000 fl
craf-1_star	0 uM	0	1000 fl
craf-1_star_star	0 uM	0	1000 fl
Raf_star-GTP-Ras	0 uM	0	1000 fl
MAPKK_star	0 uM	0	1000 fl

Equations for group /kinetics/Ras

Reactions for group /kinetics/Ras

Reaction	kf	kb	Kf	Kb
BetaGamma + inact-GEF <===> GEF-Gprot-bg	1e-05 #^-1.s^-1	1 s^-1	6 uM^-1.s^-1	1 s^-1
GEF_star <===> inact-GEF	1 s^-1	0 s^-1	1 s^-1	0 s^-1
GTP-Ras <===> GDP-Ras	0.0001 s^-1	0 s^-1	0.0001 s^-1	0 s^-1
GAP_star <===> GAP	0.1 s^-1	0 s^-1	0.1 s^-1	0 s^-1
inact-GEF + CaM-Ca4 <===> CaM-GEF	0.0001 #^-1.s^-1	1 s^-1	60 uM^-1.s^-1	1 s^-1
inact-GEF_star <===> inact-GEF	1 s^-1	0 s^-1	1 s^-1	0 s^-1

Enzymes for group /kinetics/Ras

Enzyme-reaction	k1	k2	k3	Km	kcat	ratio
GDP-Ras ---GEF-Gprot-bg--> GTP-Ras	3.3e-07 #^-1.s^-1	0.08 s^-1	0.02 s^-1	0.50505 uM	0.02 s^-1	4
GTP-Ras ---GAP--> GDP-Ras	8.2476e-05 #^-1.s^-1	40 s^-1	10 s^-1	1.0104 uM	10 s^-1	4
GDP-Ras ---CaM-GEF--> GTP-Ras	3.3e-07 #^-1.s^-1	0.08 s^-1	0.02 s^-1	0.50505 uM	0.02 s^-1	4
GDP-Ras ---GEF_star--> GTP-Ras	3.3e-07 #^-1.s^-1	0.08 s^-1	0.02 s^-1	0.50505 uM	0.02 s^-1	4

Pools for group /kinetics/Ras

name	InitialConc	buffered	Volume
GEF-Gprot-bg	0 uM	0	1000 fl
inact-GEF	0.1 uM	0	1000 fl
GTP-Ras	0 uM	0	1000 fl
GDP-Ras	0.5 uM	0	1000 fl
GAP	0.01 uM	0	1000 fl
CaM-GEF	0 uM	0	1000 fl
GEF_star	0 uM	0	1000 fl
inact-GEF_star	0 uM	0	1000 fl
GAP_star	0 uM	0	1000 fl

Equations for group /kinetics/PP1

Reactions for group /kinetics/PP1

Reaction	kf	kb	Kf	Kb
I1_star + PP1-active_c <===> PP1-I1_star	0.00083329 #^-1.s^-1	0.1 s^-1	499.97 uM^-1.s^-1	0.1 s^-1
PP1-I1 <===> PP1-active_c + I1	1 s^-1	0 #^-1.s^-1	1 s^-1	0 uM^-1.s^-1

Pools for group /kinetics/PP1

name	InitialConc	buffered	Volume
I1	1.8 uM	0	1000 fl
PP1-I1	0 uM	0	1000 fl
I1_star	0.001 uM	0	1000 fl
PP1-I1_star	0 uM	0	1000 fl

Equations for group /kinetics/PP2B

Reactions for group /kinetics/PP2B

Reaction	kf	kb	Kf	Kb
2 Ca + CaNAB-Ca2 <===> CaNAB-Ca4	9.9998e-12 #^-2.s^-1	1 s^-1	3.5999 uM^-2.s^-1	1 s^-1
CaNAB + 2 Ca <===> CaNAB-Ca2	2.7801e-08 #^-2.s^-1	1 s^-1	10008 uM^-2.s^-1	1 s^-1
CaNAB-Ca4 + CaM-Ca2 <===> CaMCa2-CANAB	4e-07 #^-1.s^-1	1 s^-1	0.24 uM^-1.s^-1	1 s^-1
CaNAB-Ca4 + CaM-Ca3 <===> CaMCa3-CaNAB	3.73e-06 #^-1.s^-1	1 s^-1	2.238 uM^-1.s^-1	1 s^-1
CaM-Ca4 + CaNAB-Ca4 <===> CaMCa4-CaNAB	0.001 #^-1.s^-1	1 s^-1	600 uM^-1.s^-1	1 s^-1

Pools for group /kinetics/PP2B

name	InitialConc	buffered	Volume
CaNAB	1 uM	0	1000 fl
CaNAB-Ca2	0 uM	0	1000 fl
CaMCa3-CaNAB	0 uM	0	1000 fl
CaMCa2-CANAB	0 uM	0	1000 fl
CaMCa4-CaNAB	0 uM	0	1000 fl

Equations for group /kinetics/camkiv

Reactions for group /kinetics/camkiv

Reaction	kf	kb	Kf	Kb

CaM-Ca4 + CaMKIVc <===> CaMKIV_CaM_Ca_c	2.22e-08 #^-1.s^-1	0.01 s^-1	0.01332 uM^-1.s^-1	0.01 s^-1
CaM-Ca4 + CaMKK_c <===> CaMKK_CaM_Ca_c	6.75e-06 #^-1.s^-1	0.02 s^-1	4.05 uM^-1.s^-1	0.02 s^-1
pCaMKIV_CaM_Ca_c <===> pCaMKIV_CaM_Ca_n	0.0009 s^-1	0.007 s^-1	0.0009 s^-1	0.007 s^-1

Enzymes for group /kinetics/camkiv

Enzyme-reaction	k1	k2	k3	Km	kcat	ratio
CaMKIV_CaM_Ca_c ---CaMKK_CaM_Ca_c--> pCaMKIV_Ca	7.0513e-06 #^-1.s^-1	4.4 s^-1	1.1 s^-1	1.3 uM	1.1 s^-1	4

Pools for group /kinetics/camkiv

name	InitialConc	buffered	Volume
pCaMKIV_CaM_Ca_c	0 uM	0	1000 fl
CaMKIVc	1 uM	0	1000 fl
CaMKKp	0 uM	0	1000 fl
CaMKK_c	0.5 uM	0	1000 fl
CaMKK_CaM_Ca_c	0 uM	0	1000 fl
CaMKIV_CaM_Ca_c	0 uM	0	1000 fl
pCaMKIV_CaM_Ca_c_tot	0 uM	0	1000 fl

Equations for group /kinetics/TrKB_mod

Reactions for group /kinetics/TrKB_mod

Reaction	kf	kb	Kf	Kb
BDNF_TrkB2_clx <===> BDNF_TrkB2_star_clx	0.02 s^-1	0 s^-1	0.02 s^-1	0 s^-1
BDNF_TrkB_clx + TrKB <===> BDNF_TrkB2_clx	1.6667e-06 #^-1.s^-1	0.02 s^-1	0.99999 uM^-1.s^-1	0.02 s^-1
TrKB + BDNF <===> BDNF_TrkB_clx	1.6668e-06 #^-1.s^-1	0.05 s^-1	1 uM^-1.s^-1	0.05 s^-1
BDNF_TrkB2_star_clx <===> 2 Int_BDNF_TrKB2_star_clx	0.01 s^-1	0 #^-1.s^-1	0.01 s^-1	0 uM^-1.s^-1
Int_BDNF_TrKB2_star_clx <===> TrKB	0.001 s^-1	0.001 s^-1	0.001 s^-1	0.001 s^-1
PLC_g_star <===> PLC_g	0.07 s^-1	0 s^-1	0.07 s^-1	0 s^-1

Enzymes for group /kinetics/TrKB_mod

Enzyme-reaction	k1	k2	k3	Km	kcat	ratio
PLC_g ---BDNF_TrkB2_star_clx--> PLC_g_star	1.3889e-05 #^-1.s^-1	2 s^-1	0.5 s^-1	0.30001 uM	0.5 s^-1	4
Shc ---BDNF_TrkB2_star_clx--> Shc_star	3.0003e-06 #^-1.s^-1	1.2 s^-1	0.3 s^-1	0.83328 uM	0.3 s^-1	4

Pools for group /kinetics/TrKB_mod

name	InitialConc	buffered	Volume
TrKB	0.25 uM	0	999.93 fl
BDNF_TrkB2_clx	0 uM	0	999.97 fl
BDNF_TrkB_clx	0 uM	0	999.93 fl
BDNF_TrkB2_star_clx	0 uM	0	999.97 fl
Int_BDNF_TrKB2_star_clx	0.25 uM	1	1000 fl

Same data sorting by data type:

Equations for group /##[]

Reactions for group /##[]

Reaction	kf	kb	Kf	Kb
Src_star <===> Src	100 s^-1	0.1 s^-1	100 s^-1	0.1 s^-1
Cbl_star <===> Cbl	10 s^-1	0.01 s^-1	10 s^-1	0.01 s^-1
C3G + CRK <===> CRK_C3G	1.6667e-06 #^-1.s^-1	0.002 s^-1	1 uM^-1.s^-1	0.002 s^-1
Rap1GTP <===> Rap1GDP	0.0001 s^-1	0 s^-1	0.0001 s^-1	0 s^-1
Rap1GTP + bRaf <===> bRaf_Rap1GTP	0.0001 #^-1.s^-1	0.5 s^-1	60 uM^-1.s^-1	0.5 s^-1
MAPK_star <===> MAPK_star_n	0.0001 s^-1	0.003 s^-1	0.0001 s^-1	0.003 s^-1
active_RSK2 <===> active_RSK2_n	0.001 s^-1	0.005 s^-1	0.001 s^-1	0.005 s^-1
pRSK <===> ppRSK	0.1 s^-1	10 s^-1	0.1 s^-1	10 s^-1
Ca_input <===> Ca	100 s^-1	100 s^-1	100 s^-1	100 s^-1
Grb2 + Sos <===> Sos.Grb2	4.1667e-07 #^-1.s^-1	0.0168 s^-1	0.25 uM^-1.s^-1	0.0168 s^-1
Sos.Grb2 + Shc_star <===> Shc_star.Sos.Grb2	8.3333e-06 #^-1.s^-1	0.1 s^-1	5 uM^-1.s^-1	0.1 s^-1
Sos_star <===> Sos	0.001 s^-1	0.1 s^-1	0.001 s^-1	0.1 s^-1
CBP + MAPKstar_CREB <===> CBP_pCREB_CRE	5.1351e-07 #^-1.s^-1	0.025 s^-1	0.114 uM^-1.s^-1	0.025 s^-1
mRNA_clx <===> mRNA	1.44 s^-1	0.0001 s^-1	1.44 s^-1	0.0001 s^-1
mRNA <===> degraded_mRNA	1 s^-1	0 s^-1	1 s^-1	0 s^-1
Cbl_star + CRK_C3G <===> CRK_C3G_Cbl_star_clx	1.6667e-06 #^-1.s^-1	0.2 s^-1	1 uM^-1.s^-1	0.2 s^-1
Grb2 + Sos_star <===> Sos_star.Grb2	4.1667e-08 #^-1.s^-1	0.0168 s^-1	0.025 uM^-1.s^-1	0.0168 s^-1
TORC1c <===> TORC1n	0.01 s^-1	0.001 s^-1	0.01 s^-1	0.001 s^-1
TORC1n + CBP_pCREB_CRE <===> Transcription_clx	4.5045e-06 #^-1.s^-1	0.1 s^-1	1 uM^-1.s^-1	0.1 s^-1
SIK2_star <===> SIK2	0.1 s^-1	0 s^-1	0.1 s^-1	0 s^-1
PP1-I1n <===> PP1_active_n + I1n	1 s^-1	9.009e-06 #^-1.s^-	1 s^-1	2 uM^-1.s^-1
PP1-I1 <===> PP1-I1n	0.003 s^-1	0.003 s^-1	0.003 s^-1	0.003 s^-1
PP1_active_n + I1n <===> PP1-I1n	2.2521e-06 #^-1.s^-1	0.1 s^-1	0.49997 uM^-1.s^-1	0.1 s^-1
Shc_star <===> Shc	0.2 s^-1	0 s^-1	0.2 s^-1	0 s^-1
CBP + CaMKIVstar_CREB <===> CBP_pCREB_CaMKIV	5.1351e-07 #^-1.s^-1	0.025 s^-1	0.114 uM^-1.s^-1	0.025 s^-1
CBP_pCREB_CaMKIV + TORC1n <===> Transcription_Clx_	4.5045e-06 #^-1.s^-1	0.1 s^-1	1 uM^-1.s^-1	0.1 s^-1
mRNA_clx_CaMKIV <===> mRNA_CaMKIV	1.44 s^-1	0.0001 s^-1	1.44 s^-1	0.0001 s^-1
mRNA_CaMKIV <===> deg_mRNA_CaMKIV	1 s^-1	0 s^-1	1 s^-1	0 s^-1
A_star + CREB <===> PP1star_CREB	4.5045e-06 #^-1.s^-1	0.1 s^-1	1 uM^-1.s^-1	0.1 s^-1
CREB + B_star <===> MAPKstar_CREB	4.5045e-06 #^-1.s^-1	0.1 s^-1	1 uM^-1.s^-1	0.1 s^-1
C_star + CREB <===> CaMKIVstar_CREB	4.5045e-06 #^-1.s^-1	0.1 s^-1	1 uM^-1.s^-1	0.1 s^-1
CBP + PP1star_CREB <===> CBP_pCREB_PP1	5.1351e-07 #^-1.s^-1	0.025 s^-1	0.114 uM^-1.s^-1	0.025 s^-1
CBP_pCREB_PP1 + TORC1n <===> Transcr_clx_PP1	4.5047e-06 #^-1.s^-1	0.1 s^-1	1 uM^-1.s^-1	0.1 s^-1
PP1mRNA_clx <===> PP1_mRNA	1.44 s^-1	0.0001 s^-1	1.44 s^-1	0.0001 s^-1
PP1_mRNA <===> deg_PP1mRNA	1 s^-1	0 s^-1	1 s^-1	0 s^-1
R2C2 + cAMP <===> R2C2-cAMP	9e-05 #^-1.s^-1	33 s^-1	54 uM^-1.s^-1	33 s^-1
R2C2-cAMP + cAMP <===> R2C2-cAMP2	9e-05 #^-1.s^-1	33 s^-1	54 uM^-1.s^-1	33 s^-1
R2C2-cAMP2 + cAMP <===> R2C2-cAMP3	0.000125 #^-1.s^-1	110 s^-1	75 uM^-1.s^-1	110 s^-1
cAMP + R2C2-cAMP3 <===> R2C2-cAMP4	0.000125 #^-1.s^-1	32.5 s^-1	75 uM^-1.s^-1	32.5 s^-1
R2C2-cAMP4 <===> PKA-active + R2C-cAMP4	60 s^-1	3e-05 #^-1.s^-1	60 s^-1	18 uM^-1.s^-1
R2C-cAMP4 <===> PKA-active + R2-cAMP4	60 s^-1	3e-05 #^-1.s^-1	60 s^-1	18 uM^-1.s^-1
R2-cAMP4 <===> R2 + cAMP	0.0005 s^-1	1.6667e-10 #^-1.s^	0.0005 s^-1	0.0001 uM^-1.s^-1
PKA-active + R2 <===> R2C1	0.0013317 #^-1.s^-1	0.186 s^-1	799.02 uM^-1.s^-1	0.186 s^-1
PKA-active + R2C1 <===> R2C2	0.0013317 #^-1.s^-1	0.186 s^-1	799.02 uM^-1.s^-1	0.186 s^-1
PKA-active + PKA-inhibitor <===> inhibited-PKA	0.0001 #^-1.s^-1	1 s^-1	60 uM^-1.s^-1	1 s^-1
PKA-active <===> PKA-active_n	0.000305 s^-1	0.00125 s^-1	0.000305 s^-1	0.00125 s^-1
CaM-Ca4 + AC1 <===> AC1-CaM	8.3333e-05 #^-1.s^-1	1 s^-1	50 uM^-1.s^-1	1 s^-1
AC2_star <===> AC2	0.1 s^-1	0 s^-1	0.1 s^-1	0 s^-1
cAMP-PDE_star <===> cAMP-PDE	0.1 s^-1	0 s^-1	0.1 s^-1	0 s^-1
PDE1 + CaM-Ca4 <===> CaM.PDE1	0.0012 #^-1.s^-1	5 s^-1	720 uM^-1.s^-1	5 s^-1
CaM-Ca3 + Ca <===> CaM-Ca4	7.7501e-07 #^-1.s^-1	10 s^-1	0.46501 uM^-1.s^-1	10 s^-1
CaM + Ca <===> CaM-Ca	1.4141e-05 #^-1.s^-1	8.4853 s^-1	8.4846 uM^-1.s^-1	8.4853 s^-1
CaM-Ca2 + Ca <===> CaM-Ca3	6.0001e-06 #^-1.s^-1	10 s^-1	3.6001 uM^-1.s^-1	10 s^-1
CaM-Ca + Ca <===> CaM-Ca2	1.4141e-05 #^-1.s^-1	8.4853 s^-1	8.4846 uM^-1.s^-1	8.4853 s^-1
craf-1 + GTP-Ras <===> Raf-GTP-Ras	1e-05 #^-1.s^-1	1 s^-1	6 uM^-1.s^-1	1 s^-1

craf-1_star + GTP-Ras <===> Raf_star-GTP-Ras	1.6666e-05 #^-1.s^-1	0.5 s^-1	9.9996 uM^-1.s^-1	0.5 s^-1
bRaf + GTP-Ras <===> braf-GTP-Ras	0.0001 #^-1.s^-1	0.5 s^-1	60 uM^-1.s^-1	0.5 s^-1
BetaGamma + inact-GEF <===> GEF-Gprot-bg	1e-05 #^-1.s^-1	1 s^-1	6 uM^-1.s^-1	1 s^-1
GEF_star <===> inact-GEF	1 s^-1	0 s^-1	1 s^-1	0 s^-1
GTP-Ras <===> GDP-Ras	0.0001 s^-1	0 s^-1	0.0001 s^-1	0 s^-1
GAP_star <===> GAP	0.1 s^-1	0 s^-1	0.1 s^-1	0 s^-1
inact-GEF + CaM-Ca4 <===> CaM-GEF	0.0001 #^-1.s^-1	1 s^-1	60 uM^-1.s^-1	1 s^-1
inact-GEF_star <===> inact-GEF	1 s^-1	0 s^-1	1 s^-1	0 s^-1
I1_star + PP1-active_c <===> PP1-I1_star	0.00083329 #^-1.s^-1	0.1 s^-1	499.97 uM^-1.s^-1	0.1 s^-1
PP1-I1 <===> PP1-active_c + I1	1 s^-1	0 #^-1.s^-1	1 s^-1	0 uM^-1.s^-1
2 Ca + CaNAB-Ca2 <===> CaNAB-Ca4	9.9998e-12 #^-2.s^-1	1 s^-1	3.5999 uM^-2.s^-1	1 s^-1
CaNAB + 2 Ca <===> CaNAB-Ca2	2.7801e-08 #^-2.s^-1	1 s^-1	10008 uM^-2.s^-1	1 s^-1
CaNAB-Ca4 + CaM-Ca2 <===> CaMCa2-CANAB	4e-07 #^-1.s^-1	1 s^-1	0.24 uM^-1.s^-1	1 s^-1
CaNAB-Ca4 + CaM-Ca3 <===> CaMCa3-CaNAB	3.73e-06 #^-1.s^-1	1 s^-1	2.238 uM^-1.s^-1	1 s^-1
CaM-Ca4 + CaNAB-Ca4 <===> CaMCa4-CaNAB	0.001 #^-1.s^-1	1 s^-1	600 uM^-1.s^-1	1 s^-1
CaM-Ca4 + CaMKIVc <===> CaMKIV_CaM_Ca_c	2.22e-08 #^-1.s^-1	0.01 s^-1	0.01332 uM^-1.s^-1	0.01 s^-1
CaM-Ca4 + CaMKK_c <===> CaMKK_CaM_Ca_c	6.75e-06 #^-1.s^-1	0.02 s^-1	4.05 uM^-1.s^-1	0.02 s^-1
pCaMKIV_CaM_Ca_c <===> pCaMKIV_CaM_Ca_n	0.0009 s^-1	0.007 s^-1	0.0009 s^-1	0.007 s^-1
BDNF_TrkB2_clx <===> BDNF_TrkB2_star_clx	0.02 s^-1	0 s^-1	0.02 s^-1	0 s^-1
BDNF_TrkB_clx + TrKB <===> BDNF_TrkB2_clx	1.6667e-06 #^-1.s^-1	0.02 s^-1	0.99999 uM^-1.s^-1	0.02 s^-1
TrKB + BDNF <===> BDNF_TrkB_clx	1.6668e-06 #^-1.s^-1	0.05 s^-1	1 uM^-1.s^-1	0.05 s^-1
BDNF_TrkB2_star_clx <===> 2 Int_BDNF_TrKB2_star_clx	0.01 s^-1	0 #^-1.s^-1	0.01 s^-1	0 uM^-1.s^-1
Int_BDNF_TrKB2_star_clx <===> TrKB	0.001 s^-1	0.001 s^-1	0.001 s^-1	0.001 s^-1
PLC_g_star <===> PLC_g	0.07 s^-1	0 s^-1	0.07 s^-1	0 s^-1

Enzymes for group /##[]

Enzyme-reaction	k1	k2	k3	Km	kcat	ratio
AC2 ---PKC-active--> AC2_star	1e-06 #^-1.s^-1	16 s^-1	4 s^-1	33.333 uM	4 s^-1	4
GAP ---PKC-active--> GAP_star	1e-05 #^-1.s^-1	16 s^-1	4 s^-1	3.3333 uM	4 s^-1	4
inact-GEF ---PKC-active--> GEF_star	1e-05 #^-1.s^-1	16 s^-1	4 s^-1	3.3333 uM	4 s^-1	4
craf-1 ---PKC-active--> craf-1_star	4.9999e-07 #^-1.s^-1	16 s^-1	4 s^-1	66.668 uM	4 s^-1	4
cAMP-PDE ---PKA-active--> cAMP-PDE_star	1e-05 #^-1.s^-1	36 s^-1	9 s^-1	7.5 uM	9 s^-1	4
Src ---PKA-active--> Src_star	0.0033334 #^-1.s^-1	80 s^-1	20 s^-1	0.049999 uM	20 s^-1	4
inact-GEF ---PKA-active--> inact-GEF_star	1e-05 #^-1.s^-1	36 s^-1	9 s^-1	7.5 uM	9 s^-1	4
I1 ---PKA-active--> I1_star	1e-05 #^-1.s^-1	36 s^-1	9 s^-1	7.5 uM	9 s^-1	4
CaMKK_CaM_Ca_c ---PKA-active--> CaM-Ca4 + CaMKKp	1.2116e-06 #^-1.s^-1	2.7333 s^-1	0.68333 s^-1	4.6999 uM	0.68333 s^-1	4
SIK2 ---PKA-active--> SIK2_star	1.8117e-07 #^-1.s^-1	0.4 s^-1	0.1 s^-1	4.5997 uM	0.1 s^-1	4
I1n ---PKA-active_n--> I1_star_n	2.7027e-05 #^-1.s^-1	36 s^-1	9 s^-1	7.5 uM	9 s^-1	4
bRaf_Rap1GTP ---Rap1GAP--> Rap1GDP + bRaf	0.00033667 #^-1.s^-1	200 s^-1	2 s^-1	0.99999 uM	2 s^-1	100
Rap1GTP ---Rap1GAP--> Rap1GDP	0.00033667 #^-1.s^-1	200 s^-1	2 s^-1	0.99999 uM	2 s^-1	100
MAPKK-ser ---bRaf_Rap1GTP--> MAPKK_star	1.5625e-05 #^-1.s^-1	1.2 s^-1	0.3 s^-1	0.16 uM	0.3 s^-1	4
MAPKK ---bRaf_Rap1GTP--> MAPKK-ser	1.5625e-05 #^-1.s^-1	1.2 s^-1	0.3 s^-1	0.16 uM	0.3 s^-1	4
MAPK-tyr ---MKP-1--> MAPK	0.00025 #^-1.s^-1	16 s^-1	4 s^-1	0.13333 uM	4 s^-1	4
MAPK_star ---MKP-1--> MAPK-tyr	0.00025 #^-1.s^-1	16 s^-1	4 s^-1	0.13333 uM	4 s^-1	4
craf-1_star ---PPhosphatase2A--> craf-1	3.1935e-06 #^-1.s^-1	24 s^-1	6 s^-1	15.657 uM	6 s^-1	4
MAPKK_star ---PPhosphatase2A--> MAPKK-ser	3.1935e-06 #^-1.s^-1	24 s^-1	6 s^-1	15.657 uM	6 s^-1	4
MAPKK-ser ---PPhosphatase2A--> MAPKK	3.1935e-06 #^-1.s^-1	24 s^-1	6 s^-1	15.657 uM	6 s^-1	4
craf-1_star_star ---PPhosphatase2A--> craf-1_star	3.1935e-06 #^-1.s^-1	24 s^-1	6 s^-1	15.657 uM	6 s^-1	4
ppRSK ---PDK1--> active_RSK2	8.3333e-07 #^-1.s^-1	4 s^-1	1 s^-1	10 uM	1 s^-1	4
active_RSK2 ---PP2A--> ppRSK	9.4692e-07 #^-1.s^-1	4 s^-1	1 s^-1	8.8005 uM	1 s^-1	4
I1_star ---PP2A--> I1	6.6e-06 #^-1.s^-1	25 s^-1	6 s^-1	7.8283 uM	6 s^-1	4.1667
CaMKKp ---PP2A--> CaMKK_c	1.1667e-06 #^-1.s^-1	2.8 s^-1	0.7 s^-1	4.9999 uM	0.7 s^-1	4
pCaMKIV_CaM_Ca_c ---PP2A--> CaMKIV_CaM_Ca_c	1.8939e-06 #^-1.s^-1	8 s^-1	2 s^-1	8.8002 uM	2 s^-1	4
pRSK ---PP2A--> RSK	9.4692e-07 #^-1.s^-1	4 s^-1	1 s^-1	8.8005 uM	1 s^-1	4
PP1-I1_star ---PP2A--> PP1-I1	6.6e-06 #^-1.s^-1	25 s^-1	6 s^-1	7.8283 uM	6 s^-1	4.1667
A_star ---PP1_active_n--> A	2.2523e-06 #^-1.s^-1	0.4 s^-1	0.1 s^-1	0.99998 uM	0.1 s^-1	4

B ---active_RSK2_n--> B_star	4.5045e-07 #^-1.s^-1	0.4 s^-1	0.1 s^-1	5 uM	0.1 s^-1	4
I1_star ---CaNAB-Ca4--> I1	5.7e-08 #^-1.s^-1	0.136 s^-1	0.034 s^-1	4.9708 uM	0.034 s^-1	4
B ---pMSK1_n--> B_star	1.1261e-06 #^-1.s^-1	0.4 s^-1	0.1 s^-1	2 uM	0.1 s^-1	4
pMSK1_n ---PP2An--> MSK1_n	2.5594e-06 #^-1.s^-1	4 s^-1	1 s^-1	8.7999 uM	1 s^-1	4
I1_star_n ---PP2An--> I1n	1.7262e-05 #^-1.s^-1	24 s^-1	6 s^-1	7.8285 uM	6 s^-1	4
PLC_g ---PLCg_basal--> PLC_g_star	1.3889e-05 #^-1.s^-1	2 s^-1	0.5 s^-1	0.3 uM	0.5 s^-1	4
Nucleotides ---Basal_transcription--> mRNA_clx	1.0416e-06 #^-1.s^-1	0.2 s^-1	0.05 s^-1	1.0812 uM	0.05 s^-1	4
Nucleotides ---Basal_transcription--> mRNA_clx_CaMKIV	1.0427e-06 #^-1.s^-1	0.2 s^-1	0.05 s^-1	1.08 uM	0.05 s^-1	4
Nucleotides ---Basal_transcription--> PP1mRNA_clx	1.0427e-06 #^-1.s^-1	0.2 s^-1	0.05 s^-1	1.08 uM	0.05 s^-1	4
C ---pCaMKIV_CaM_Ca_nuc--> C_star	2.8665e-06 #^-1.s^-1	2.8 s^-1	0.7 s^-1	5.5 uM	0.7 s^-1	4
GDP-Ras ---Shc_star.Sos.Grb2--> GTP-Ras	3.3e-05 #^-1.s^-1	0.8 s^-1	0.2 s^-1	0.050505 uM	0.2 s^-1	4
Rap1GDP ---CRK_C3G_Cbl_star_clx--> Rap1GTP	6.6668e-05 #^-1.s^-1	0.2 s^-1	0.2 s^-1	0.0099998 uM	0.2 s^-1	1
Cbl ---Src_star--> Cbl_star	0.00066665 #^-1.s^-1	160 s^-1	40 s^-1	0.50001 uM	40 s^-1	4
MSK1_n ---MAPK_star_n--> pMSK1_n	4.2496e-07 #^-1.s^-1	0.4 s^-1	0.1 s^-1	5.2999 uM	0.1 s^-1	4
Sos ---MAPK_star--> Sos_star	3.2552e-05 #^-1.s^-1	40 s^-1	10 s^-1	2.56 uM	10 s^-1	4
RSK ---MAPK_star--> pRSK	2.673e-06 #^-1.s^-1	6.8 s^-1	1.7 s^-1	5.2999 uM	1.7 s^-1	4
craf-1_star ---MAPK_star--> craf-1_star_star	3.25e-06 #^-1.s^-1	40 s^-1	10 s^-1	25.641 uM	10 s^-1	4
Nucleotides ---Transcription_clx--> mRNA_clx	1.0427e-06 #^-1.s^-1	0.2 s^-1	0.05 s^-1	1.08 uM	0.05 s^-1	4
TORC1c ---SIK2--> pTORC1	8.3333e-07 #^-1.s^-1	1.6 s^-1	0.4 s^-1	4 uM	0.4 s^-1	4
I1_star ---CaM_Ca_n-CaNAB--> I1	5.7e-07 #^-1.s^-1	1.36 s^-1	0.34 s^-1	4.9708 uM	0.34 s^-1	4
pTORC1 ---CaM_Ca_n-CaNAB--> TORC1c	2.0833e-06 #^-1.s^-1	0.4 s^-1	0.1 s^-1	0.40001 uM	0.1 s^-1	4
PP1-I1_star ---CaM_Ca_n-CaNAB--> PP1-I1	5.7e-07 #^-1.s^-1	1.36 s^-1	0.34 s^-1	4.9708 uM	0.34 s^-1	4
Nucleotides ---Transcription_Clx_C--> mRNA_clx_CaMKIV	1.0427e-06 #^-1.s^-1	0.2 s^-1	0.05 s^-1	1.08 uM	0.05 s^-1	4
A ---A_kinase--> A_star	2.2524e-06 #^-1.s^-1	0.4 s^-1	0.1 s^-1	0.99998 uM	0.1 s^-1	4
B_star ---B_phosphatase--> B	2.2523e-06 #^-1.s^-1	0.4 s^-1	0.1 s^-1	0.99998 uM	0.1 s^-1	4
C_star ---C_phospatase--> C	2.2524e-06 #^-1.s^-1	0.4 s^-1	0.1 s^-1	0.99998 uM	0.1 s^-1	4
Nucleotides ---Transcr_clx_PP1--> PP1mRNA_clx	1.0427e-06 #^-1.s^-1	0.2 s^-1	0.05 s^-1	1.08 uM	0.05 s^-1	4
ATP ---AC1-CaM--> cAMP	7.5e-06 #^-1.s^-1	72 s^-1	18 s^-1	20 uM	18 s^-1	4
cAMP ---cAMP-PDE--> AMP	4.2e-06 #^-1.s^-1	40 s^-1	10 s^-1	19.841 uM	10 s^-1	4
cAMP ---PDE1--> AMP	3.5e-07 #^-1.s^-1	6.67 s^-1	1.667 s^-1	39.7 uM	1.667 s^-1	4.0012
cAMP ---CaM.PDE1--> AMP	2.1e-06 #^-1.s^-1	40 s^-1	10 s^-1	39.683 uM	10 s^-1	4
ATP ---AC2_star--> cAMP	2.9e-06 #^-1.s^-1	28 s^-1	7 s^-1	20.115 uM	7 s^-1	4
cAMP ---cAMP-PDE_star--> AMP	8.4e-06 #^-1.s^-1	80 s^-1	20 s^-1	19.841 uM	20 s^-1	4
MAPKK ---Raf-GTP-Ras--> MAPKK-ser	1.5714e-05 #^-1.s^-1	1.2 s^-1	0.3 s^-1	0.15909 uM	0.3 s^-1	4
MAPKK-ser ---Raf-GTP-Ras--> MAPKK_star	1.5714e-05 #^-1.s^-1	1.2 s^-1	0.3 s^-1	0.15909 uM	0.3 s^-1	4
MAPKK-ser ---braf-GTP-Ras--> MAPKK_star	1.0417e-05 #^-1.s^-1	0.8 s^-1	0.2 s^-1	0.15999 uM	0.2 s^-1	4
MAPKK ---braf-GTP-Ras--> MAPKK-ser	1.0417e-05 #^-1.s^-1	0.8 s^-1	0.2 s^-1	0.15999 uM	0.2 s^-1	4
MAPKK-ser ---Raf_star-GTP-Ras--> MAPKK_star	1.5714e-05 #^-1.s^-1	1.2 s^-1	0.3 s^-1	0.15909 uM	0.3 s^-1	4
MAPKK ---Raf_star-GTP-Ras--> MAPKK-ser	1.5714e-05 #^-1.s^-1	1.2 s^-1	0.3 s^-1	0.15909 uM	0.3 s^-1	4
MAPK ---MAPKK_star--> MAPK-tyr	5.4e-05 #^-1.s^-1	1.2 s^-1	0.3 s^-1	0.046296 uM	0.3 s^-1	4
MAPK-tyr ---MAPKK_star--> MAPK_star	5.4e-05 #^-1.s^-1	1.2 s^-1	0.3 s^-1	0.046296 uM	0.3 s^-1	4
GDP-Ras ---GEF-Gprot-bg--> GTP-Ras	3.3e-07 #^-1.s^-1	0.08 s^-1	0.02 s^-1	0.50505 uM	0.02 s^-1	4
GTP-Ras ---GAP--> GDP-Ras	8.2476e-05 #^-1.s^-1	40 s^-1	10 s^-1	1.0104 uM	10 s^-1	4
GDP-Ras ---CaM-GEF--> GTP-Ras	3.3e-07 #^-1.s^-1	0.08 s^-1	0.02 s^-1	0.50505 uM	0.02 s^-1	4
GDP-Ras ---GEF_star--> GTP-Ras	3.3e-07 #^-1.s^-1	0.08 s^-1	0.02 s^-1	0.50505 uM	0.02 s^-1	4
CaMKIV_CaM_Ca_c ---CaMKK_CaM_Ca_c--> pCaMKIV_Ca	7.0513e-06 #^-1.s^-1	4.4 s^-1	1.1 s^-1	1.3 uM	1.1 s^-1	4
PLC_g ---BDNF_TrkB2_star_clx--> PLC_g_star	1.3889e-05 #^-1.s^-1	2 s^-1	0.5 s^-1	0.30001 uM	0.5 s^-1	4
Shc ---BDNF_TrkB2_star_clx--> Shc_star	3.0003e-06 #^-1.s^-1	1.2 s^-1	0.3 s^-1	0.83328 uM	0.3 s^-1	4

Pools for group /##[]

name	InitialConc	buffered	Volume
CaM-Ca4	0 uM	0	1000 fl
PKC-active	0.01 uM	1	1000 fl
PKA-active	0 uM	0	1000 fl
cAMP	0 uM	0	1000 fl
PKA-active_n	0 uM	0	370 fl

Ca	0.08 uM	0	1000 fl
Src	0.02 uM	0	1000 fl
Cbl	0.5 uM	0	1000 fl
C3G	0.5 uM	0	1000 fl
CRK	1 uM	0	1000 fl
CRK_C3G	0 uM	0	1000 fl
Rap1GTP	0 uM	0	1000 fl
Rap1GDP	0.2 uM	0	1000 fl
Rap1GAP	0.012 uM	0	1000 fl
bRaf_Rap1GTP	0 uM	0	1000 fl
bRaf	0.2 uM	0	1000 fl
MKP-1	0.015 uM	0	1000 fl
PPhosphatase2A	1 uM	0	1000 fl
MSK1_n	0.2 uM	0	370 fl
PDK1	1 uM	0	1000 fl
PP2A	0.15 uM	1	1000 fl
PP1_active_n	0 uM	0	370 fl
ppRSK	0 uM	0	1000 fl
active_RSK2	0 uM	0	1000 fl
active_RSK2_n	0 uM	0	370 fl
RSK	0.2 uM	0	1000 fl
pRSK	0 uM	0	1000 fl
BetaGamma	0 uM	0	1000 fl
CaNAB-Ca4	0 uM	0	1000 fl
PP1-active_c	1.8 uM	0	1000 fl
pMSK1_n	0 uM	0	370 fl
Ca_input	0 uM	0	1000 fl
PP2An	0.1 uM	0	370 fl
BDNF	5e-05 uM	1	999.97 fl
PLC_g	0.1 uM	0	1000 fl
PLCg_basal	0.0007 uM	0	1000 fl
Grb2	1 uM	0	1000 fl
Shc	0.5 uM	0	1000 fl
Sos	0.1 uM	0	1000 fl
Sos.Grb2	0 uM	0	1000 fl
CBP	0.5 uM	0	370 fl
mRNA_clx	0 uM	0	370 fl
Basal_transcription	5e-05 uM	0	370 fl
mRNA	0 uM	0	370 fl
degraded_mRNA	0 uM	1	370 fl
Basal_CaMKIV	0.0005 uM	0	1000 fl
pCaMKIV_CaM_Ca_nuc	0 uM	0	370 fl
pCaMKIV_CaM_Ca_n	0 uM	0	370 fl
Basal_CaMKIV_n	5e-05 uM	0	370 fl
Sum_total_CaMKIV	0 uM	0	1000 fl
MAPK_active_total	0 uM	0	1000 fl
Basal_MAPK_active	0.0001 uM	0	1000 fl
Shc_star	0 uM	0	1000 fl
PLC_g_star	0 uM	0	1000 fl
Sos_star	0 uM	0	1000 fl
Shc_star.Sos.Grb2	0 uM	0	1000 fl
Sos_star.Grb2	0 uM	0	1000 fl
CRK_C3G_Cbl_star_clx	0 uM	0	1000 fl
Cbl_star	0 uM	0	1000 fl
Src_star	0 uM	0	1000 fl
MAPK_star_n	0 uM	0	370 fl

MAPK_star	0 uM	0	1000 fl
pTORC1	0 uM	0	1000 fl
TORC1n	0 uM	0	370 fl
TORC1c	0.1 uM	0	1000 fl
CBP_pCREB_CRE	0 uM	0	370 fl
Transcription_clx	0 uM	0	370 fl
Nucleotides	0.2 uM	1	369.98 fl
SIK2	0.5 uM	0	1000 fl
SIK2_star	0 uM	0	1000 fl
PP1-I1n	0 uM	0	370 fl
I1n	0 uM	0	370 fl
I1_star_n	0 uM	0	369.98 fl
CaM_Ca_n-CaNAB	0 uM	0	1000 fl
CBP_pCREB_CaMKIV	0 uM	0	370 fl
Transcription_Clx_C	0 uM	0	370 fl
mRNA_clx_CaMKIV	0 uM	0	370 fl
mRNA_CaMKIV	0 uM	0	370 fl
deg_mRNA_CaMKIV	0 uM	1	370 fl
B	0.1 uM	0	370 fl
B_star	0 uM	0	370 fl
C	0.1 uM	0	370 fl
C_star	0 uM	0	370 fl
A_star	0 uM	0	370 fl
A	0.1 uM	0	369.98 fl
A_kinase	0.1 uM	0	369.98 fl
CREB	0.5 uM	0	370 fl
B_phosphatase	0.1 uM	0	370 fl
C_phospatase	0.1 uM	0	369.98 fl
MAPKstar_CREB	0 uM	0	370 fl
CaMKIVstar_CREB	0 uM	0	370 fl
PP1star_CREB	0 uM	0	370 fl
CBP_pCREB_PP1	0 uM	0	369.98 fl
Transcr_clx_PP1	0 uM	0	370 fl
PP1mRNA_clx	0 uM	0	370 fl
PP1_mRNA	0 uM	0	370 fl
deg_PP1mRNA	0 uM	1	370 fl
total_PP1_acive	0 uM	0	1000 fl
R2C2	0.5 uM	0	1000 fl
R2C2-cAMP	0 uM	0	1000 fl
R2C2-cAMP2	0 uM	0	1000 fl
R2C2-cAMP3	0 uM	0	1000 fl
R2C2-cAMP4	0 uM	0	1000 fl
R2C-cAMP4	0 uM	0	1000 fl
R2-cAMP4	0 uM	0	1000 fl
R2	0 uM	0	1000 fl
R2C1	0 uM	0	1000 fl
inhibited-PKA	0 uM	0	1000 fl
PKA-inhibitor	0.25 uM	0	1000 fl
ATP	5000 uM	1	1000 fl
AC1-CaM	0 uM	0	1000 fl
AC1	0.02 uM	0	1000 fl
AC2	0.015 uM	0	1000 fl
AMP	3.2549e+05 uM	0	0.0016667 fl
cAMP-PDE	0.45 uM	0	1000 fl
PDE1	2 uM	0	1000 fl
CaM.PDE1	0 uM	0	1000 fl

AC2_star	0 uM	0	1000 fl
cAMP-PDE_star	0 uM	0	1000 fl
CaM	20 uM	0	1000 fl
CaM-Ca3	0 uM	0	1000 fl
CaM-Ca2	0 uM	0	1000 fl
CaM-Ca	0 uM	0	1000 fl
craf-1	0.2 uM	0	1000 fl
MAPKK	0.18 uM	0	1000 fl
MAPK	0.36 uM	0	1000 fl
MAPK-tyr	0 uM	0	1000 fl
MAPKK-ser	0 uM	0	1000 fl
Raf-GTP-Ras	0 uM	0	1000 fl
braf-GTP-Ras	0 uM	0	1000 fl
craf-1_star	0 uM	0	1000 fl
craf-1_star_star	0 uM	0	1000 fl
Raf_star-GTP-Ras	0 uM	0	1000 fl
MAPKK_star	0 uM	0	1000 fl
GEF-Gprot-bg	0 uM	0	1000 fl
inact-GEF	0.1 uM	0	1000 fl
GTP-Ras	0 uM	0	1000 fl
GDP-Ras	0.5 uM	0	1000 fl
GAP	0.01 uM	0	1000 fl
CaM-GEF	0 uM	0	1000 fl
GEF_star	0 uM	0	1000 fl
inact-GEF_star	0 uM	0	1000 fl
GAP_star	0 uM	0	1000 fl
I1	1.8 uM	0	1000 fl
PP1-I1	0 uM	0	1000 fl
I1_star	0.001 uM	0	1000 fl
PP1-I1_star	0 uM	0	1000 fl
CaNAB	1 uM	0	1000 fl
CaNAB-Ca2	0 uM	0	1000 fl
CaMCa3-CaNAB	0 uM	0	1000 fl
CaMCa2-CANAB	0 uM	0	1000 fl
CaMCa4-CaNAB	0 uM	0	1000 fl
pCaMKIV_CaM_Ca_c	0 uM	0	1000 fl
CaMKIVc	1 uM	0	1000 fl
CaMKKp	0 uM	0	1000 fl
CaMKK_c	0.5 uM	0	1000 fl
CaMKK_CaM_Ca_c	0 uM	0	1000 fl
CaMKIV_CaM_Ca_c	0 uM	0	1000 fl
pCaMKIV_CaM_Ca_c_tot	0 uM	0	1000 fl
TrKB	0.25 uM	0	999.93 fl
BDNF_TrkB2_clx	0 uM	0	999.97 fl
BDNF_TrkB_clx	0 uM	0	999.93 fl
BDNF_TrkB2_star_clx	0 uM	0	999.97 fl
Int_BDNF_TrKB2_star_clx	0.25 uM	1	1000 fl
