# Supplementary material for: Transcription Control Pathways Decode Patterned Synaptic Inputs into Diverse mRNA Expression Profiles
Source: PLoS One. 2014 May 1;9(5):e95154. doi: 10.1371/journal.pone.0095154 (PMC4006808; doi:10.1371/journal.pone.0095154)
Supplement: File S1 — Supporting figures. Figure S1. Diagram of existed signaling models incorporated into the current model. Figure S2. Chemical reaction diagram of MAPK pathway showing activation of MAPK by bRaf and cRaf. Figure S3. Phosphorylated MAPK as a function of time and chemical reaction diagram of submodels. Figure S4. Parameter sensitivity analysis for Total_pCaMKIV. Figure S5. Parameter sensitivity analysis for Total_MAPK. Figure S6. Parameter sensitivity analysis for pCREB_CRE. Figure S7. Sensitivity of mRNA synthesis rate for various calcium-input patterns. Figure S8. Total_pCaMKIV and pCREB-CRE as a function of Ca2+ level. Figure S9. Response of model to combinations of Ca2+ and BDNF stimulation. (PDF) [file pone.0095154.s004.pdf]

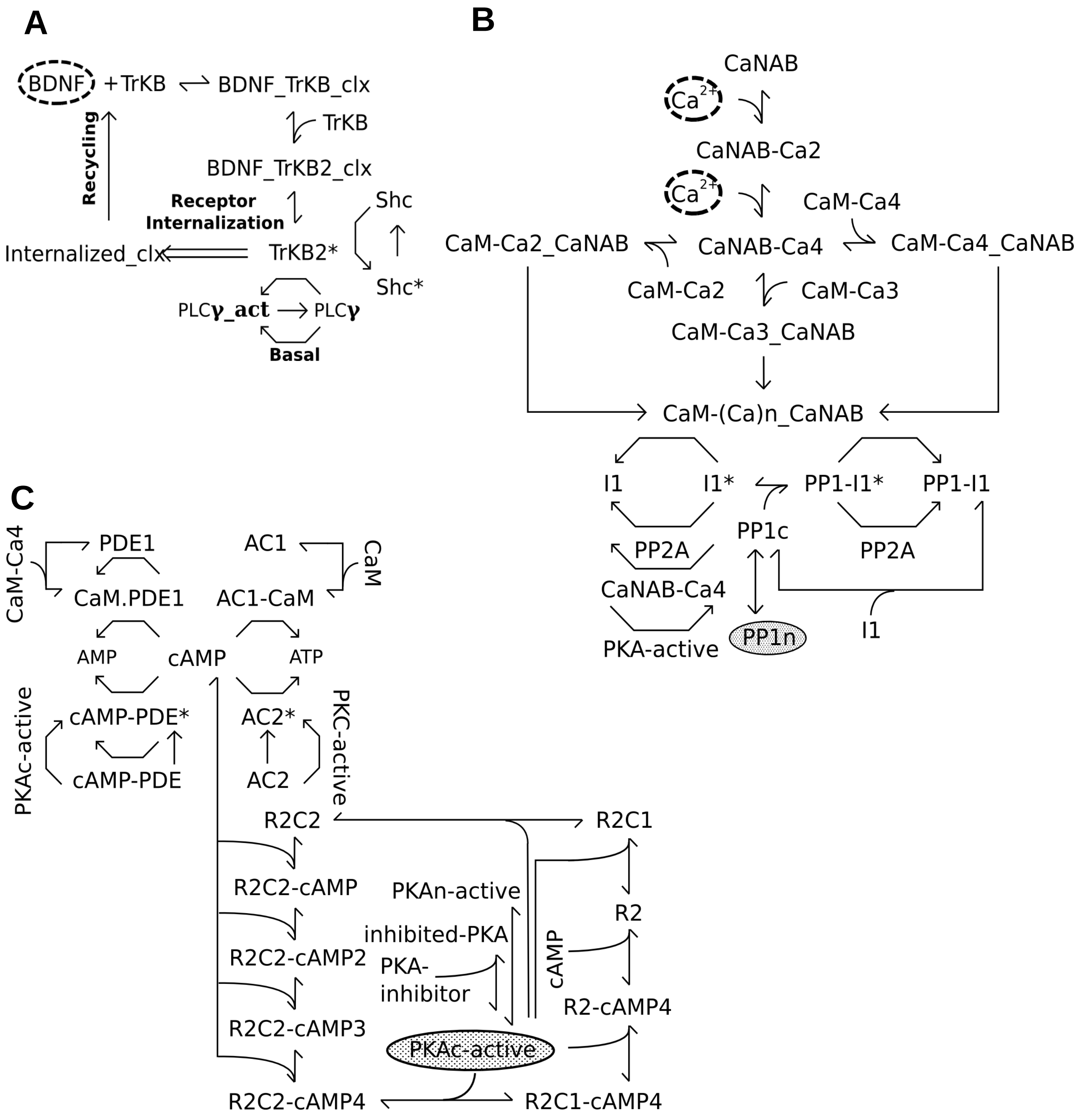

Figure S1. Diagram of existed signaling models incorporated into the current model.

(A) Chemical reaction diagram of published model of BDNF input pathway.

(B) Chemical reaction diagram of published model of PP1 pathway.

(C) Chemical reaction diagram of published model of PKA pathway.

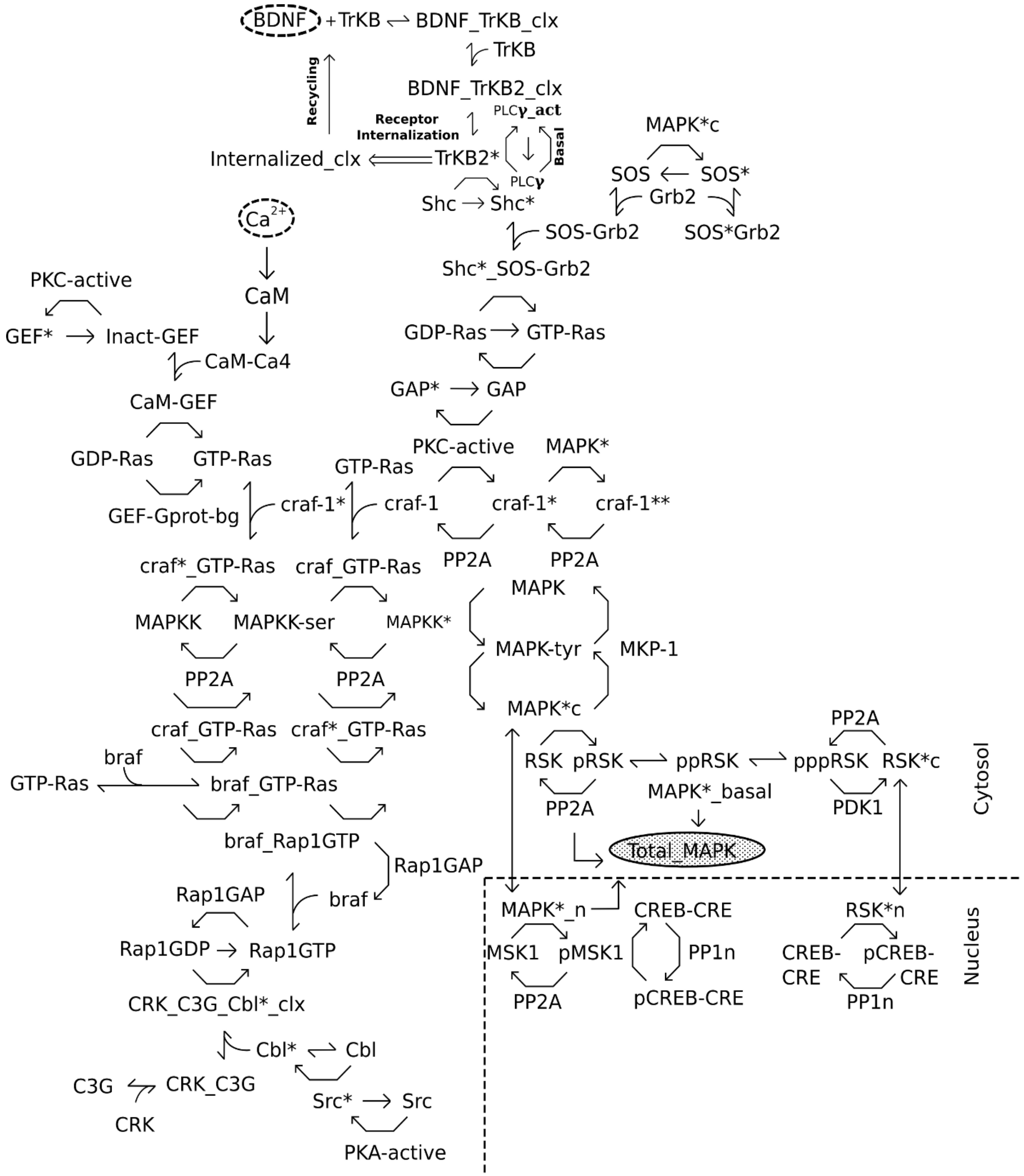

Figure S2. Chemical reaction diagram of MAPK pathway showing activation of MAPK by bRaf and cRaf. The model further includes downstream steps involving RSK, PDK1, and nuclear transport leading to activation of CREB-CRE. The inputs to the pathway are Ca^{2+} and BDNF.

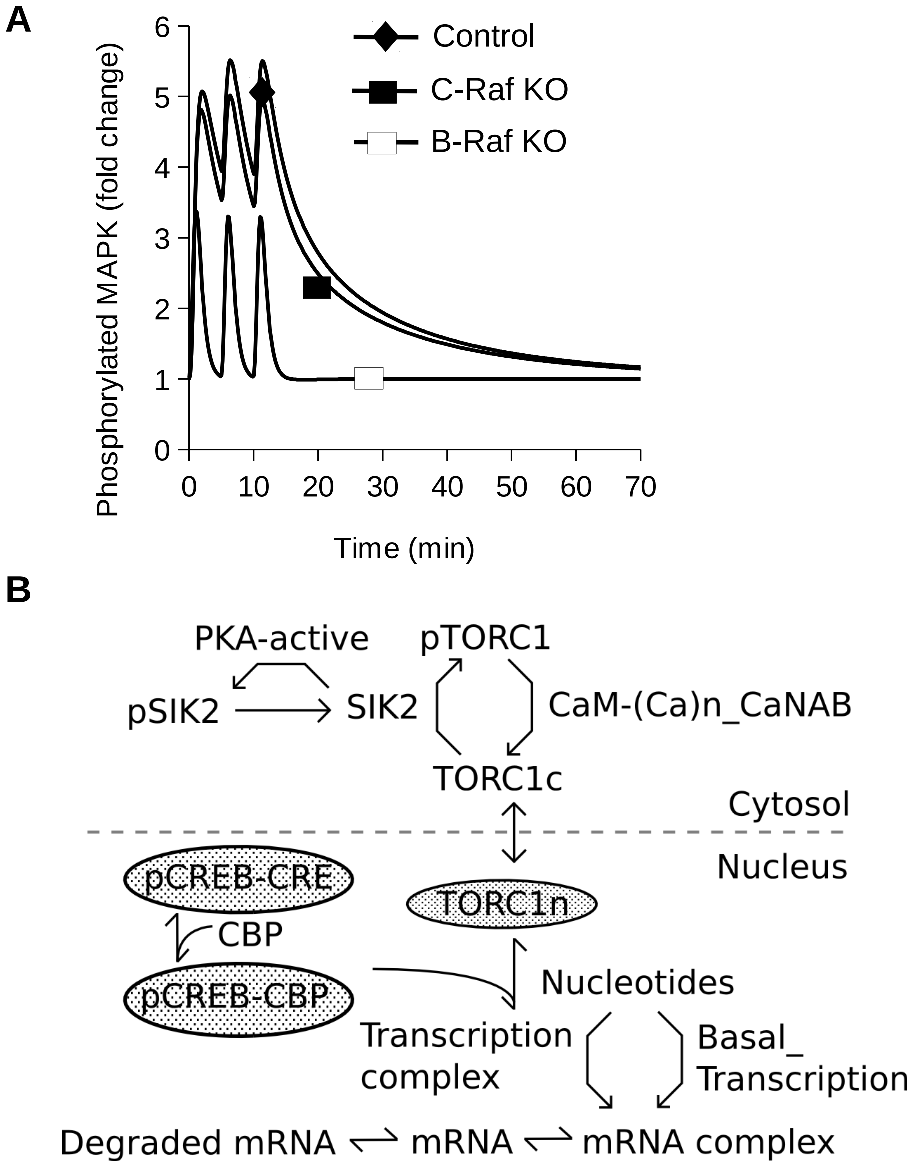

Figure S3. Phosphorylated MAPK as a function of time and chemical reaction diagram of submodels.

(A) The B-Raf pathway contributes to slow phase of active MAPK formation.

(B) Chemical reaction diagram of CREB, TORC1 and mRNA synthesis submodel.

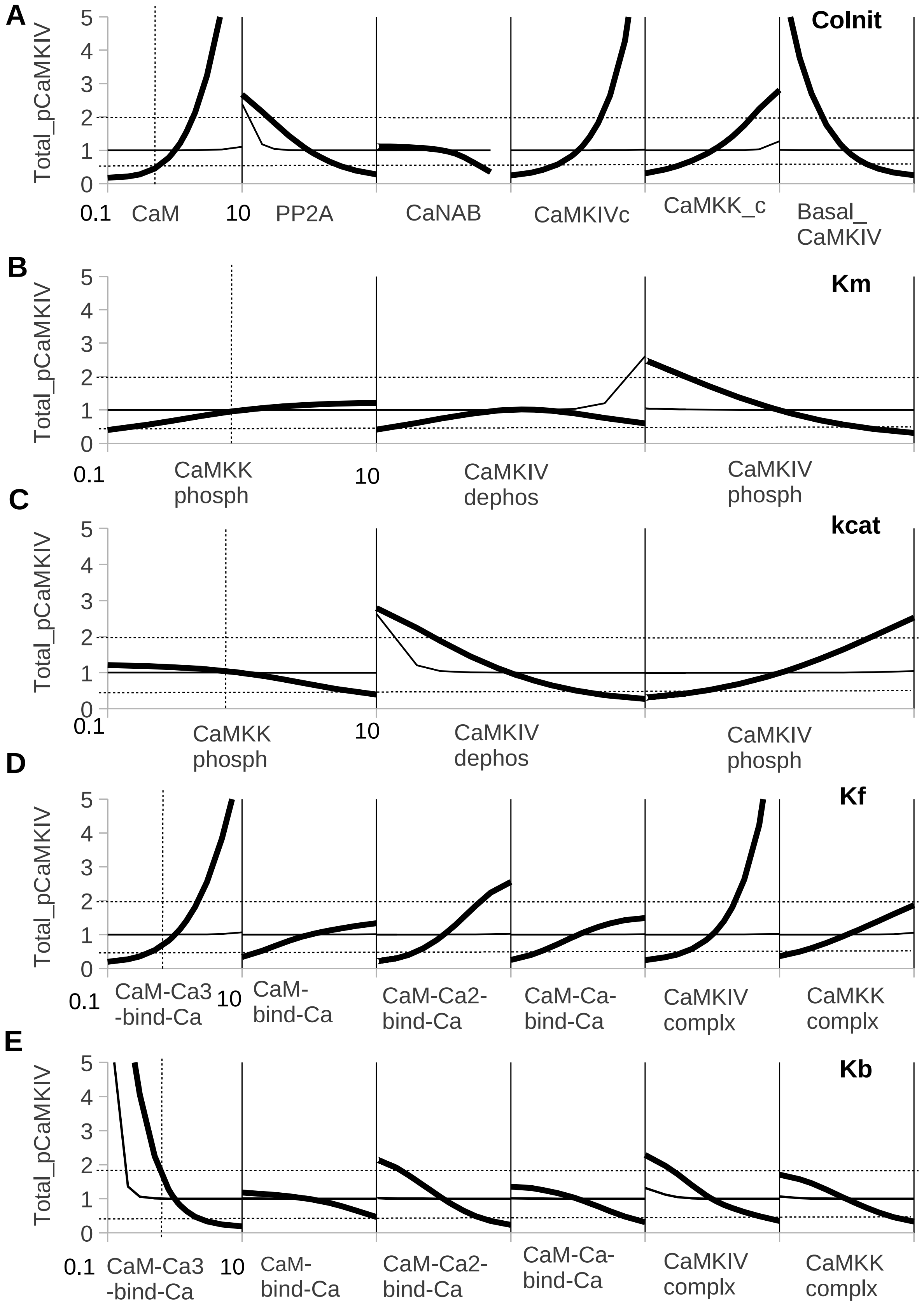

Figure S4. Parameter sensitivity analysis for Total_pCaMKIV. We systematically varied the CoInit, Km, kcat, kf and kb from 0.1 to 10 fold the original model value.

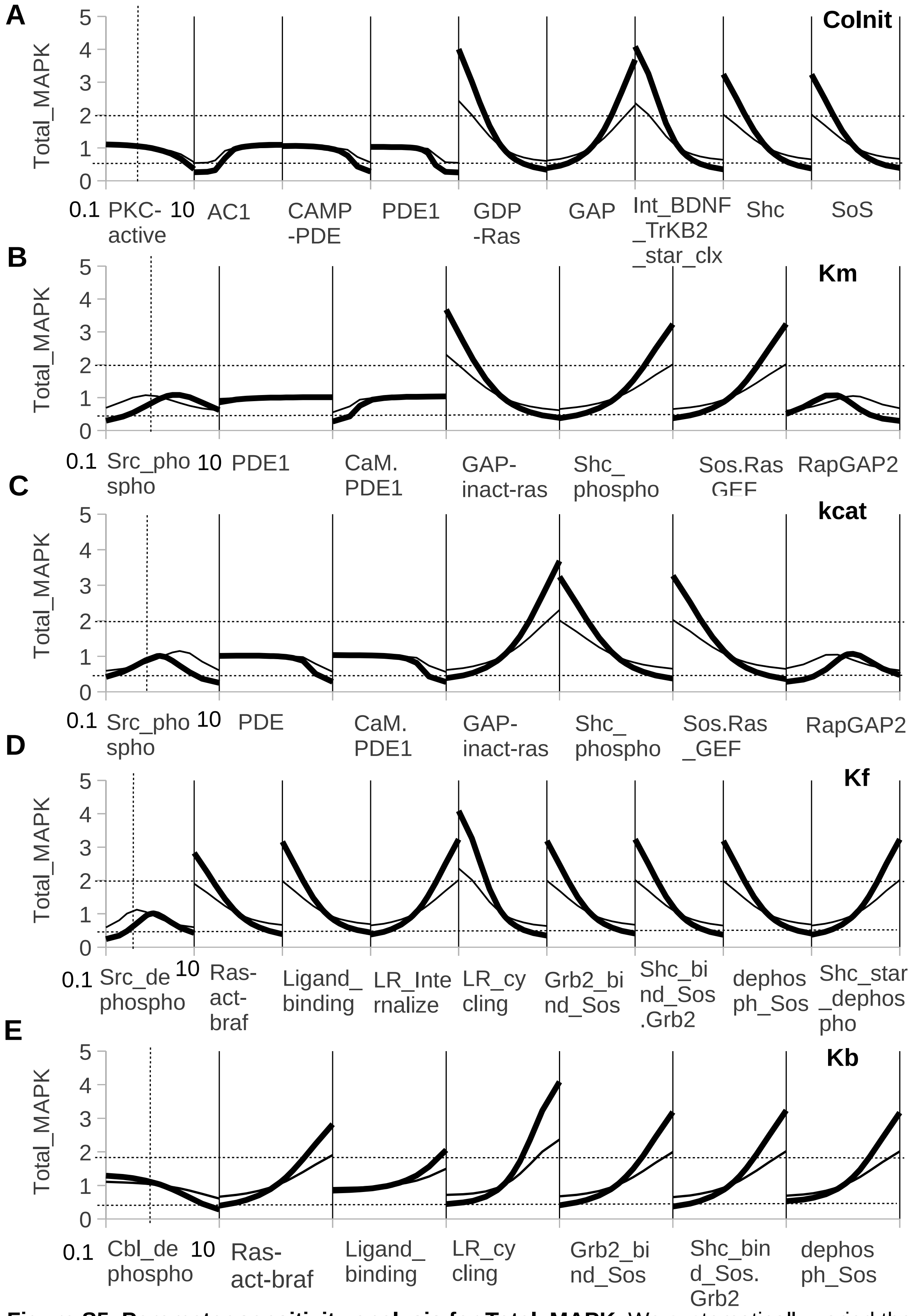

Figure S5. Parameter sensitivity analysis for Total_MAPK. We systematically varied the CoInit, Km, kcat, kf and kb from 0.1 to 10 fold the original model value.

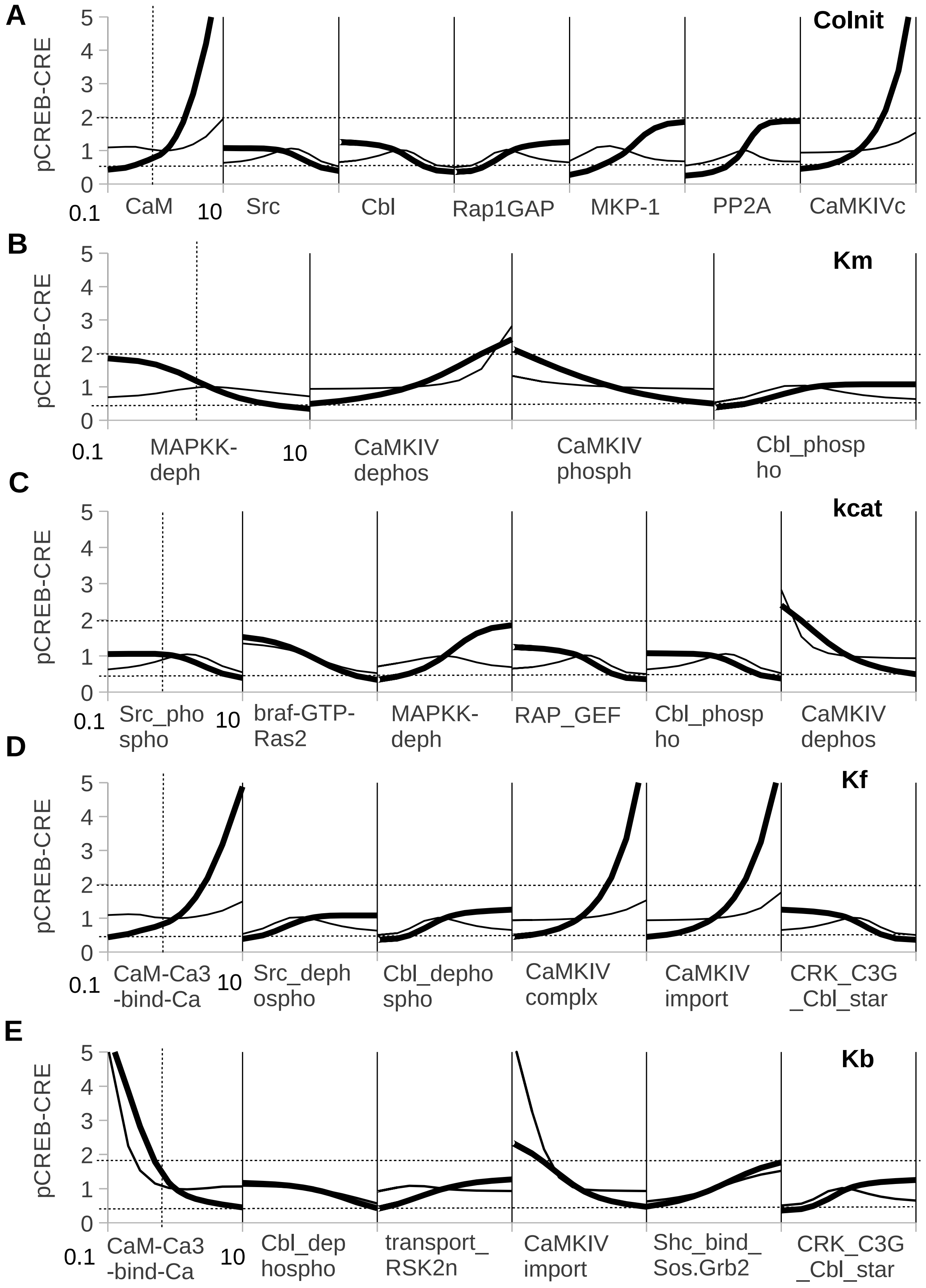

Figure S6. Parameter sensitivity analysis for pCREB_CRE. We systematically varied the CoInit, Km, kcat, kf and kb from 0.1 to 10 fold the original model value.

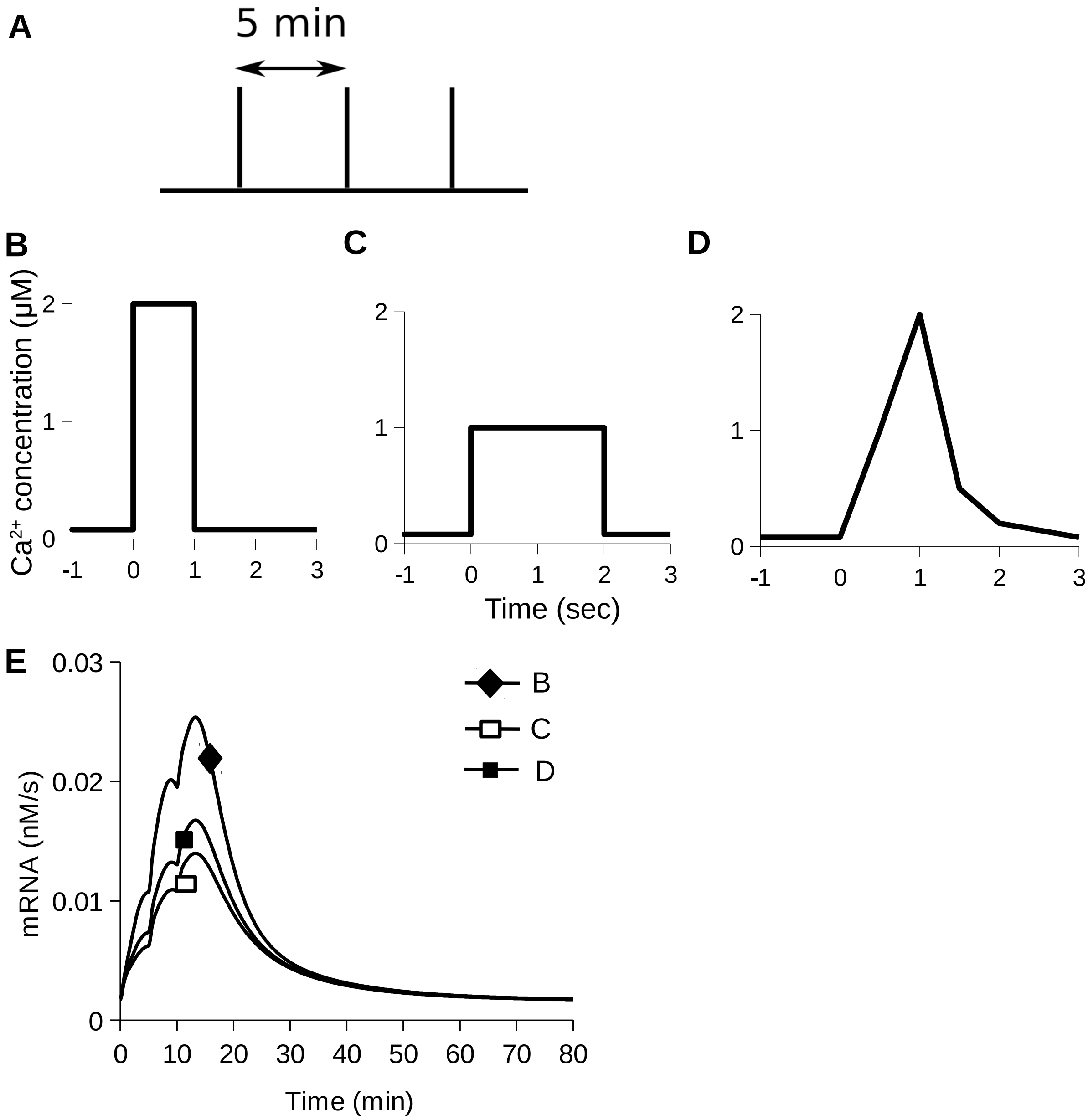

Figure S7. Sensitivity of mRNA synthesis rate for various calcium-input patterns. The stimuli presented were (A) three pulses of Ca^{2+} presented with a 5 min spacing. (B) Each pulse was of 2 μM amplitude and 1 sec wide. (C) Each pulse was of 1 μM amplitude and 2 sec wide. (D) Each pulse reached a peak of 2 μM amplitude near the end of the pulse and then decayed with a half-time of ~5 sec to the baseline level. (E) mRNA synthesis was moderately sensitive to the peak Ca^{2+} levels.

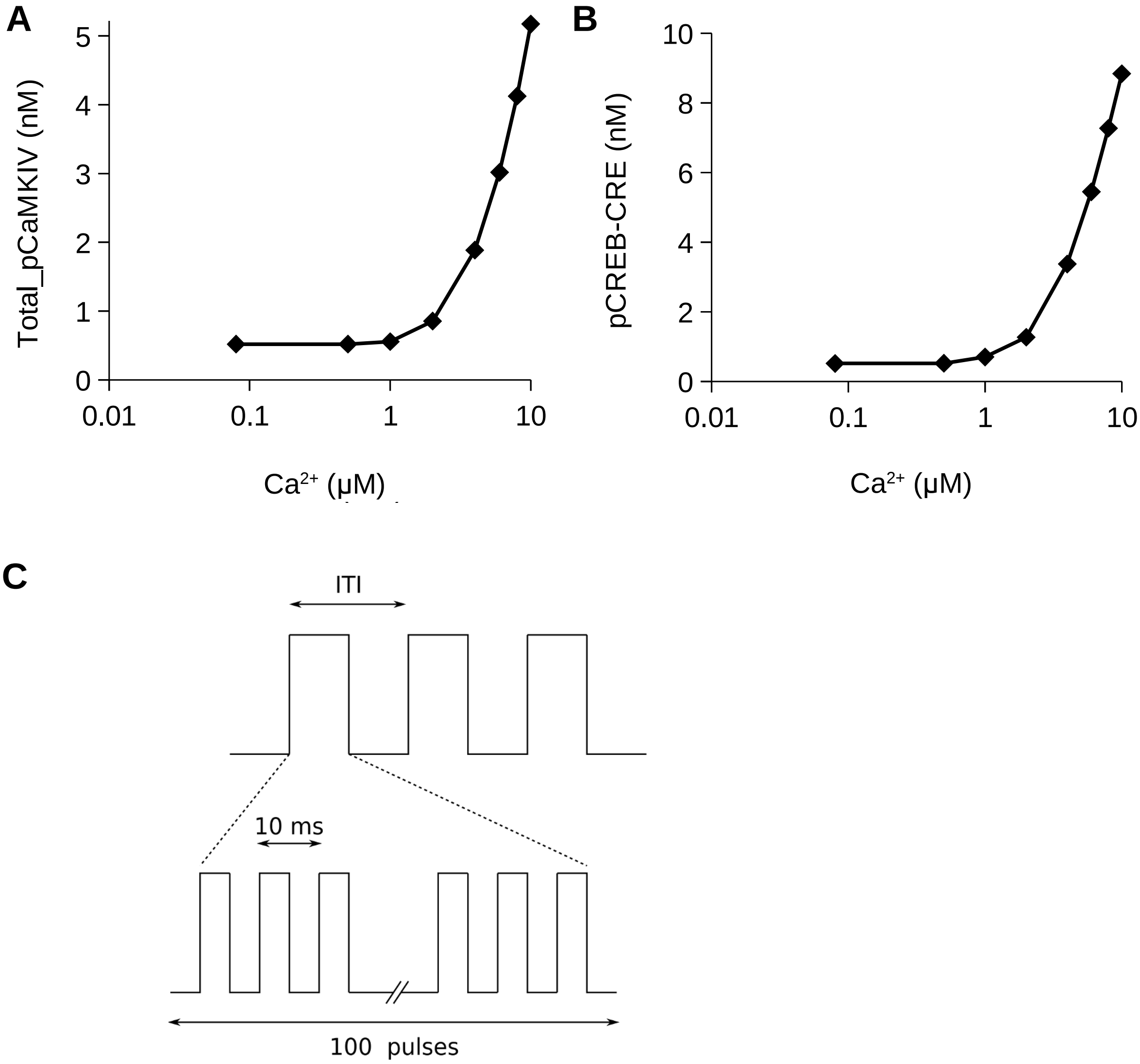

Figure S8. Total_pCaMKIV and pCREB-CRE as a function of Ca^{2+} level.
(A) Total_pCaMKIV and (B) pCREB-CRE as a function of Ca^{2+} level.
(C) Stimulus pattern to examine tuning of model to strong inputs. Three tetani of 100 Hz for 1 sec were delievered seperated by an inter tetanus intervals (ITI). The Ca^{2+} stimulus duration and amplitude were held constant for 1 sec and 2 μM respectiviely.

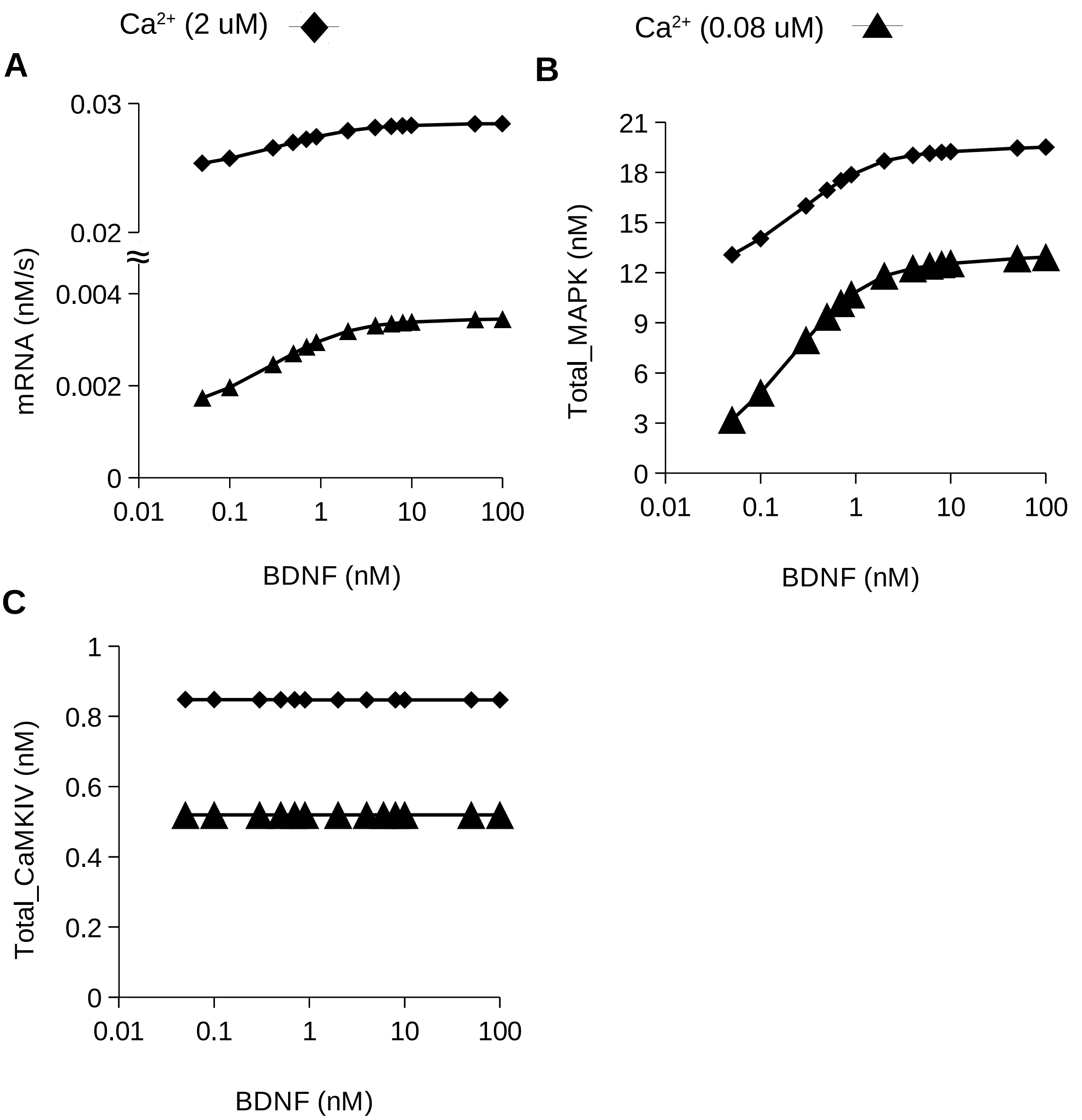

Figure S9. Response of model to combinations of Ca^{2+} and BDNF stimulation, measured 18 minutes after onset of stimulus. Triangles: Ca^{2+} held at baseline (0.08 uM), diamonds: three Ca^{2+} pulses of 2 μM for 1 sec each, separated by 5 min. In both cases, the steady stimulus of BDNF was applied at indicated concentration. (A) Protein synthesis. At baseline Ca^{2+} the mRNA synthesis level responds to BDNF, but at high Ca^{2+} the response is saturated and BDNF causes very little change in the mRNA synthesis rate. (B) Total MAPK activation. MAPK underwent some activation by BDNF both with and without the Ca^{2+} stimulus. (C) Total CaMKIV activation. Here there was no response to BDNF.
